# Forecasting the prevalence of epilepsy in low- and middle-income countries to 2050 using a hybrid deep neural network-transformer modeling framework: insights from the Global Burden of Disease Study 2023

**DOI:** 10.1016/j.mmr.2026.100055

**Published:** 2026-07-23

**Authors:** Zhi-Jin Zhang, Hao-Feng Wang, Yu-Sha Cui, Shi-Nuan Lin, Han Jiao, Fan-Gang Meng, Tao Feng

**Affiliations:** a Department of Neurology, Beijing Tiantan Hospital, Capital Medical University, Beijing 100070, China; b China National Clinical Research Center for Neurological Diseases, Beijing 100070, China; c National Engineering Research Center of Visual Technology, School of Computer Science, Peking University, Beijing 100089, China; d HUST-GYENNO CNS Intelligent Digital Medicine Technology Center, School of Artificial Intelligence and Automation, Huazhong University of Science and Technology, Wuhan 430074, China; e Guangdong Provincial Engineering Technology Research Center for Medical Artificial Intelligence in Neurological Diseases, Shenzhen 518000, Guangdong, China; f Beijing Neurosurgical Institute, Beijing Tiantan Hospital, Capital Medical University, Beijing 100070, China

**Keywords:** Epilepsy, Low- and middle-income countries (LMICs), Prevalence, Forecast, Deep learning, Deep neural network (DNN)

## Abstract

**Background:**

Epilepsy is a major public health challenge affecting individuals of all ages, especially in low- and middle-income countries (LMICs). Reliable prevalence projections are critical for healthcare planning and resource allocation. This study aimed to forecast the prevalence of epilepsy and its trends in LMICs by age, sex, year, and income level by 2050.

**Methods:**

Using data from the Global Burden of Disease Study (GBD) 2023, we projected the prevalence and number of idiopathic and secondary epilepsy cases in LMICs from 2024 to 2050. We developed a hybrid deep neural network (DNN)-Transformer framework that integrates Poisson regression and Autoregressive Integrated Moving Average (ARIMA) models for prevalence projection. Decomposition analysis was applied to quantify the contributions of population growth, aging, and prevalence change to the increase in epilepsy cases. Dementia-attributable epilepsy was independently projected to address secondary causes not included in GBD 2023.

**Results:**

By 2050, the age-standardized prevalence rate (ASPR) of epilepsy in LMICs was projected to reach 907.22 per 100,000 [95% uncertainty interval (UI) 731.01−1083.56], a 33.32% increase from 2023, with cases rising to 72.04 million (95% UI 57.86−86.23), a 58.68% increase. The ASPRs of idiopathic and secondary epilepsy were estimated at 323.11 and 584.10 per 100,000 in 2050, respectively, with the increase in secondary epilepsy being more than 7-fold that of idiopathic epilepsy since 2023. The ASPR of secondary epilepsy due to neonatal disorders was projected to rise by 65.76%. Model validation demonstrated good predictive performance (root mean squared error <0.001). From 2023 to 2050, the increases in idiopathic and secondary epilepsy cases were forecast to be highest in low-income countries (LICs; 76.12% and 241.50%, respectively), with growth declining as income levels increased. Population growth (21.40%) primarily drove the increase in idiopathic epilepsy cases, whereas changes in prevalence (59.89%) predominantly drove the rise in secondary epilepsy cases. Dementia-attributable secondary epilepsy was projected to reach 3.40 million cases by 2050.

**Conclusions:**

We forecast a continuous increase in the prevalence and number of epilepsy cases in LMICs through 2050, with secondary epilepsy increasing more rapidly than idiopathic epilepsy. LICs may exhibit the greatest increases over the next three decades, necessitating targeted interventions and further investigation.

## 1 Background

Epilepsy is one of the most common neurological disorders and affects individuals of all ages, and is associated with an increased risk of premature mortality, making it a major public health challenge [1], [2]. Approximately 80% of people with epilepsy live in low- and middle-income countries (LMICs) [3]. According to the Global Burden of Disease (GBD) 2023 study, the burden of epilepsy increased across LMICs between 1990 and 2023, with low-income countries (LICs) exhibiting the highest age-standardized prevalence rate (ASPR) in 2023 [4], [5]. Persistent gaps in the diagnosis and treatment of epilepsy in LMICs lead to a deterioration in their quality of life [3]. In 2022, the World Health Organization launched the Intersectoral Global Action Plan on Epilepsy and Other Neurological Disorders (2022−2031), aiming to raise awareness, improve education, reduce stigma, and close gaps in diagnosis and treatment [6]. Accurate forecasting of epilepsy prevalence in LMICs is crucial for optimizing healthcare resource allocation, informing public health policies, and improving long-term disease management.

Previous research has demonstrated variations in the prevalence of epilepsy across different locations, age groups, sexes, and time periods [4]. Moreover, historical prevalence trends differ between idiopathic and secondary epilepsy. Secondary epilepsy, defined as cases due to neglected tropical diseases and malaria, other infectious diseases, and neonatal disorders, has increased markedly, whereas idiopathic epilepsy has remained relatively stable [4]. These differences highlight the need to project the future prevalence of both idiopathic and secondary epilepsy and to examine disparities across populations with different socioeconomic status, age, and sex.

Poisson regression models incorporating the Socio-Demographic Index (SDI), a comprehensive measure of socioeconomic development, have been widely used for forecasting non-fatal disease prevalence [7], [8], [9], [10]. Given the close association between epilepsy burden and socioeconomic disparities [11], [12], SDI represents a suitable indicator for epilepsy prevalence modeling. In our previous work, we developed a Poisson regression-based framework incorporating random walk components to project age-, sex-, and location-specific prevalence of non-fatal disease through optimal model weighting [7]. The deep neural network (DNN) can dynamically optimize model weights in a data-driven manner, capturing nonlinear relationships and compensating for potential model deficiencies through complex weight structures [13]. In addition, the Transformer module models the dynamic relationships among annual trends in the historical time series via self-attention mechanisms [14]. Together, these approaches enable more accurate and robust prevalence projections.

Using GBD 2023 data, this study aimed to develop a hybrid DNN-Transformer framework integrated with Poisson regression and Autoregressive Integrated Moving Average (ARIMA) models to project the prevalence of idiopathic and secondary epilepsy in 129 LMICs from 2024 to 2050, stratified by age, sex, year, and income level. Decomposition analysis was used to quantify the contributions of population growth, population aging, and changes in prevalence to the projected increase in epilepsy cases. To account for secondary causes not included in GBD 2023, we independently projected the prevalence of dementia-attributable secondary epilepsy.

## 2 Methods

### 2.1 Data sources

Data for this study were obtained from the GBD 2023 study, which estimates the burden of 371 diseases across 204 countries and territories. We extracted location-, age-, sex-, and year-specific prevalence data for epilepsy from 1990 to 2023 for 129 LMICs. Data were collected for both sexes across 20 age groups. These data were cleaned and validated following the GBD protocol, ensuring consistency, completeness, and adherence to established standards (https://www.healthdata.org/research-analysis/about-gbd/protocol). No additional data cleaning was performed by the authors.

We also acquired data on the three components of the SDI, including lag-distributed income per capita, average years of education among individuals aged ≥15 years, and total fertility under 25 years from the GBD 2019. To project SDI values from 2020 to 2050, we first fitted linear regression models to capture the long-term temporal trends of each component and then modeled the residual variation using an ARIMA (0,1,0) process, a parsimonious random-walk model suitable for non-stationary time-series data. Projected SDI values were subsequently calculated according to the standard GBD methodology, whereby each component was rescaled to a 0−1 index, and SDI was derived from their geometric mean [7], [15]. Age-, sex-, and year-specific population projections for 129 LMICs were obtained from GBD 2017, which provides demographic estimates from 2018 to 2100 [16].

### 2.2 Case definitions

The definition of epilepsy in GBD 2023 follows the criteria established by the International League Against Epilepsy (ILAE), defining an epilepsy case as someone with an active, recurring condition of epileptic seizures (≥2) that were unprovoked by any immediate cause and had experienced at least one epileptic seizure in the past 5 years, regardless of antiepileptic drug treatment [17], [18]. In GBD 2023, epilepsy was classified into two main categories: idiopathic epilepsy and secondary epilepsy. Idiopathic epilepsy refers to cases with unknown or genetic causes, according to the 1985 ILAE classification of epilepsies and epileptic syndromes [19]. Secondary epilepsy refers to cases with identifiable causes, such as structural abnormalities, metabolic disorders, or infections. In GBD 2023, the prevalence of secondary epilepsy resulting from causes including neglected tropical diseases and malaria (including malaria, cysticercosis, cystic echinococcosis, food-borne trematodiases, and Zika virus), other infectious diseases (including meningitis, encephalitis, and tetanus), and neonatal disorders (including neonatal preterm birth, neonatal encephalopathy due to birth asphyxia and trauma, neonatal sepsis and other neonatal infections, and hemolytic disease and other neonatal jaundice) were estimated. The International Classification of Diseases (ICD) coding system was also used to identify epilepsy, classified under code 345 in ICD-9 and codes G40–G41 in ICD-10.

### 2.3 Forecasting of epilepsy prevalence to 2050

#### 2.3.1 Forecasting epilepsy prevalence using a hybrid DNN-Transformer model

**Fig. 1:**
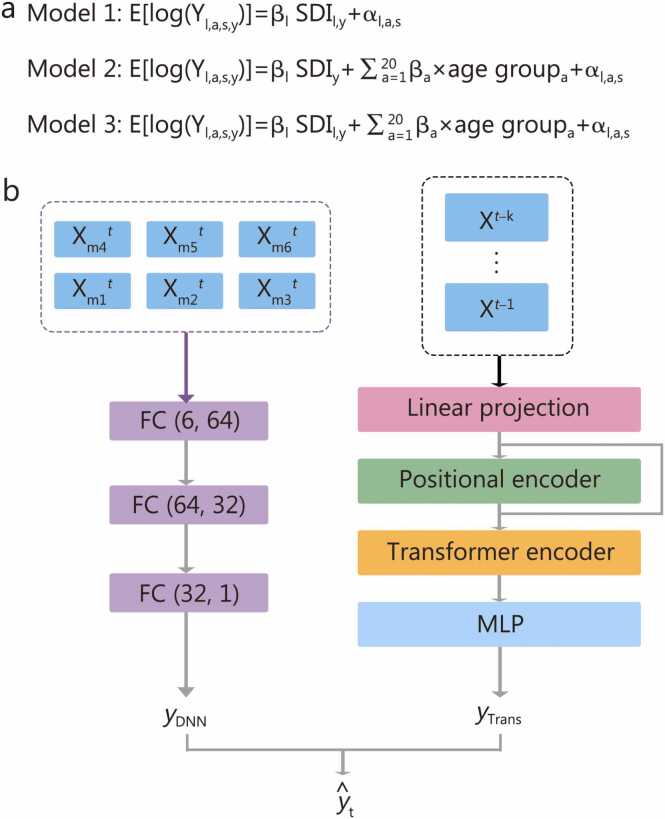
Equations and architecture of the prevalence forecasting framework. **a** Poisson regression models. Epilepsy prevalence was modeled by location, age, sex, and year, incorporating SDI, age effects, and location-, age-, sex-specific random intercepts. **b** Hybrid DNN-Transformer framework. Predictions from six models were integrated through a DNN with fully connected layers and a Transformer module capturing temporal dependencies from prior years to generate the final prevalence estimate (${\hat{y}}_{t}$). The linearly projected input embeddings were combined with positional encoding before being passed to the Transformer encoder. SDI. Socio-Demographic Index; FC. Fully connected; MLP. Multi-layer perceptron; DNN. Deep neural network; Trans. Transformer.

We developed a hybrid DNN-Transformer framework integrating Poisson regression and ARIMA to project the age-, sex-, and year-specific prevalence of idiopathic epilepsy and secondary epilepsy attributable to 11 specific causes (malaria, cysticercosis, cystic echinococcosis, food-borne trematodiases, meningitis, encephalitis, tetanus, neonatal preterm birth, neonatal encephalopathy due to birth asphyxia and trauma, neonatal sepsis and other neonatal infections, and hemolytic disease and other neonatal jaundice) across 129 LMICs from 2024 to 2050. Epilepsy due to the Zika virus was excluded from the projections due to limited historical data. We incorporated 6 prevalence projection models from our previous study, comprising Poisson regression models using SDI as a key predictor, with three models further augmented by a random walk component [ARIMA (0,1,0)] to account for residual temporal trends [7]. We then applied a deep learning approach to integrate the outputs of the six models and incorporated historical time-series information to improve prediction accuracy (Fig. 1). The model was trained using data from 1990 to 2010 and validated on data from 2011 to 2023. Training was performed for 30,000 epochs with a batch size of 4, using root mean squared error (RMSE) as the loss function. Model parameters were optimized using the AdamW optimizer with an initial learning rate of 1×10^-4^ and a weight decay of 1×10^-5^. The learning rate was adjusted using a plateau-based scheduler, whereby the learning rate was reduced by half if the validation RMSE did not improve for 1000 consecutive epochs, with a minimum learning rate of 1×10^-6^. The final model checkpoint was selected according to the lowest RMSE on the validation set, and no separate early-stopping criterion was applied. The Transformer encoder comprised 4 layers with 8 attention heads each, with a model dimension of 512. The dimensionality of the feed-forward network was set to 2048 (4× the model dimension). We employed sinusoidal positional encodings and a dropout rate of 0.1. Uncertainty was propagated using 1000 Monte Carlo draws throughout the modeling process, and 95% uncertainty intervals (UIs) were defined as the 2.5th and 97.5th percentiles of the resulting draws [8]. Detailed modeling procedures are provided in **Additional file 1: Methods**.

#### 2.3.2 Estimation of dementia-attributable secondary epilepsy prevalence

The prevalence of dementia-attributable secondary epilepsy was estimated by multiplying the pooled prevalence of epilepsy among individuals with dementia [3.17%, 95% confidence interval (CI) 0.15−6.19, *I*^2^ = 99.9%], which was recalculated by pooling estimates from two published meta-analyses (**Additional file 1:** Fig. S1) [20], [21], [22], [23], [24], [25], [26], by projected dementia prevalence derived from a prior regression-based forecasting model that incorporated future trends in education and major dementia risk factors, including smoking, high body-mass index, and high fasting plasma glucose [8]. We assumed that the prevalence of epilepsy among individuals with dementia remained constant across regions and over time due to the lack of robust region-specific and time-specific data; however, this simplifying assumption, together with the substantial between study heterogeneity observed in the meta-analysis, introduces uncertainty into the estimated pooled prevalence, and the resulting estimates should therefore be interpreted with caution.

### 2.4 Statistical analysis

Temporal trends in epilepsy prevalence were assessed using Joinpoint regression analysis, with the average annual percent change (AAPC) and corresponding 95% CI calculated using the parametric method implemented in the Joinpoint Regression Program version 5.0.1 (US National Cancer Institute, Bethesda, MD, USA) [27]. Das Gupta decomposition analysis was used to estimate the contributions of population growth, population aging, and changes in prevalence rates to changes in epilepsy cases from 2023 to 2050 [28]. Statistical analyses were performed using Python version 3.12.10 and R version 4.5.1.

## 3 Results

### 3.1 Projection of epilepsy prevalence in LMICs from 2024 to 2050

#### 3.1.1 Overall and etiological projections of epilepsy prevalence in LMICs

**Fig. 2:**
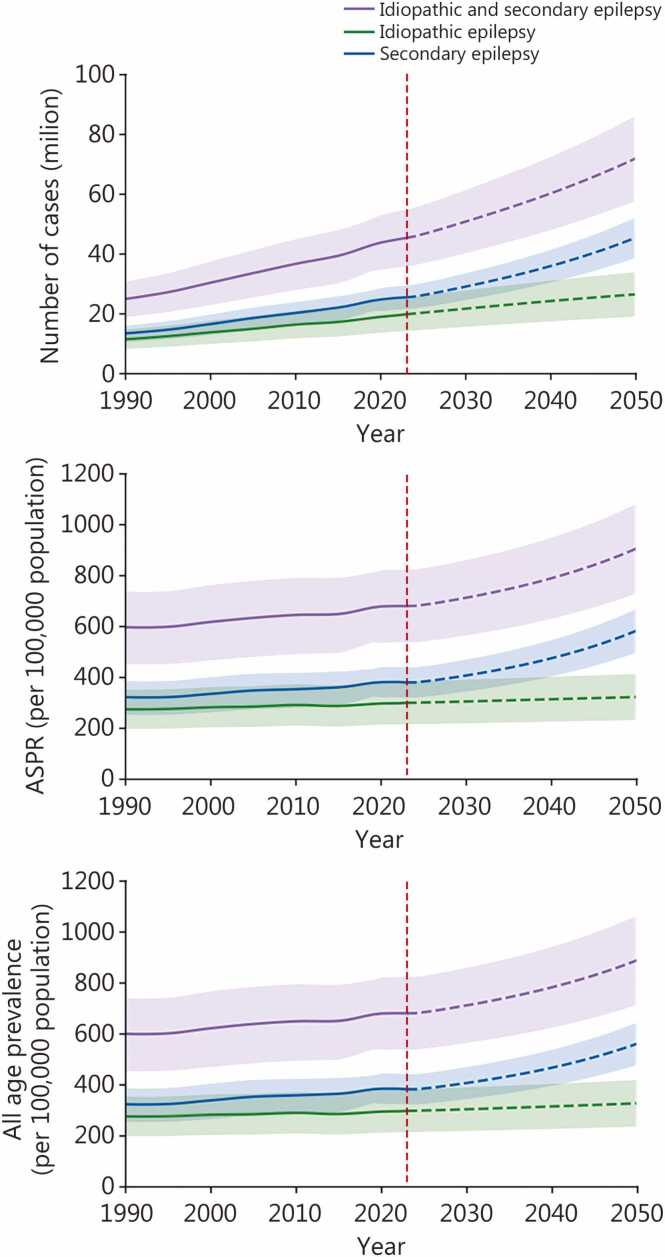
Projected trends in number of cases, ASPR, and all age prevalence with 95% uncertainty intervals of epilepsy (idiopathic and secondary), idiopathic epilepsy, and secondary epilepsy in low- and middle-income countries (LMICs), both sexes, 1990−2050. The vertical dashed line indicates the transition from the observed values (1990−2023) to the projected values (2024-2050). ASPR. Age-standardized prevalence rate.

The number of cases of epilepsy (idiopathic and secondary) and ASPR in LMICs were projected to increase by 58.68% and 33.32%, from 2023 to 2050, respectively (Table 1). By 2050, the number of all epilepsy cases in LMICs was estimated to reach 72.04 million (95% UI 57.86−86.23), comprising 26.53 million (95% UI 19.11−33.96) idiopathic epilepsy cases and 45.51 million (95% UI 38.75−52.28) secondary epilepsy cases. The ASPR of epilepsy in LMICs was projected to reach 907.22 per 100,000 (95% UI 731.01−1083.56), including 323.11 per 100,000 (95% UI 232.73−413.54) for idiopathic epilepsy and 584.10 per 100,000 (95% UI 498.27−670.02) for secondary epilepsy (Table 1). From 2023 to 2050, the prevalence of secondary epilepsy was projected to increase more rapidly than idiopathic epilepsy, with increases in prevalent numbers of 78.23% (95% UI 43.88−121.46) and 33.56% (95% UI −12.15 to 102.04), respectively, alongside the ASPR rising by 53.59% (95% UI 23.99−90.38) for secondary epilepsy and 7.64% (95% UI −28.69 to 62.14) for idiopathic epilepsy (Table 1**;** Fig. 2). Compared with historical trends for 1990–2023, projections for 2023–2050 revealed divergent ASPR trajectories for idiopathic and secondary epilepsy. The growth rate of idiopathic epilepsy remained relatively stable [AAPC 0.27 (95% CI 0.27−0.28) vs. 0.26 (95% CI 0.25−0.28)], whereas secondary epilepsy showed accelerated growth [AAPC 1.60 (95% CI 1.59−1.62) vs. 0.51 (95% CI 0.49−0.53)] (**Additional file 1:** Table S1).

**Table 1:** Projected number of cases, age-standardized prevalence rate (ASPR), and all age prevalence of epilepsy (idiopathic and secondary), idiopathic epilepsy, and secondary epilepsy in low- and middle-income countries (LMICs) in 2023 and 2050, along with percent changes from 2023 to 2050, both sexes (95% UI).

**Income level**	**Number of cases**			**ASPR**				**All age prevalence**		
	**2023 (million)**	**2050 (million)**	**Percent change (%)**	**2023 (per 100,000)**	**2050 (per 100,000)**		**Percent change (%)**	**2023 (per 100,000)**	**2050 (per 100,000)**	**Percent change (%)**
**Epilepsy (idiopathic and secondary)**										
LMICs	45.40 (35.90−54.90)	72.04 (57.86−86.23)	58.68 (18.67−113.52)	680.47 (537.40−823.61)	907.22 (731.01−1083.56)		33.32 (−0.29 to 79.52)	681.22 (538.69−823.82)	890.45 (715.17−1065.87)	30.71 (−2.32 to 75.17)
U-MICs	17.74 (13.94−21.53)	21.29 (16.77−25.82)	20.07 (−12.93 to 53.33)	628.76 (494.75−762.84)	744.47 (591.29−897.68)		18.40 (−13.76 to 50.78)	621.94 (488.89−755.07)	735.06 (578.87−891.29)	18.19 (−14.56 to 51.15)
L-MICs	22.48 (17.81−27.15)	36.83 (29.61−44.05)	63.83 (25.97−102.23)	718.10 (567.72−868.59)	950.77 (765.04−1136.79)		32.40 (−0.60 to 65.81)	725.26 (574.74−875.89)	935.58 (752.25−1119.19)	29.00 (−3.44 to 61.77)
LICs	5.18 (4.15−6.22)	13.92 (11.48−16.36)	168.46 (118.09−219.78)	703.53 (559.60−847.29)	1083.59 (891.34−1275.69)		54.02 (20.54−88.29)	726.84 (581.13−872.39)	1107.26 (913.16−1301.23)	52.34 (19.59−85.86)
**Idiopathic epilepsy**										
LMICs	19.86 (14.30−25.43)	26.53 (19.11−33.96)	33.56 (−12.15 to 102.04)	300.18 (216.12−384.22)	323.11 (232.73−413.54)	7.64 (−28.69 to 62.14)		298.07 (214.60−381.53)	327.94 (236.21−419.71)	10.02 (−26.90 to 65.31)
U-MICs	8.38 (6.03−10.72)	9.80 (7.05−12.55)	17.01 (−25.78 to 60.12)	297.78 (214.41−381.14)	321.65 (231.44−411.88)	8.02 (−33.08 to 49.25)		293.78 (211.53−376.03)	338.37 (243.48−433.25)	15.18 (−27.30 to 57.90)
L-MICs	9.20 (6.62−11.77)	12.70 (9.15−16.24)	38.04 (−8.68 to 85.88)	297.88 (214.41−381.36)	325.07 (234.29−415.97)	9.13 (−32.09 to 50.52)		296.74 (213.59−379.88)	322.53 (232.47−412.69)	8.69 (−32.45 to 49.99)
LICs	2.29 (1.65−2.93)	4.03 (2.91−5.16)	76.12 (20.21−132.70)	318.66 (229.55−407.60)	323.09 (232.74−413.31)	1.39 (−38.25 to 41.02)		321.04 (231.30−410.62)	320.86 (231.15−410.47)	−0.06 (−39.38 to 39.22)
**Secondary epilepsy**										
LMICs	25.53 (21.60−29.48)	45.51 (38.75−52.28)	78.23 (43.88−121.46)	380.29 (321.28−439.39)	584.10 (498.27−670.02)	53.59 (23.99−90.38)		383.15 (324.09−442.29)	562.51 (478.96−646.16)	46.81 (17.92−82.65)
U-MICs	9.36 (7.91−10.81)	11.49 (9.72−13.27)	22.81 (−1.48 to 47.35)	330.98 (280.34−381.70)	422.82 (359.85−485.80)	27.75 (3.55−52.27)		328.16 (277.36−379.04)	396.70 (335.39−458.04)	20.89 (−3.16 to 45.19)
L-MICs	13.28 (11.19−15.37)	24.13 (20.46−27.81)	81.69 (50.26−113.53)	420.22 (353.31−487.24)	625.71 (530.76−720.82)	48.90 (21.69−76.64)		428.53 (361.15−496.00)	613.05 (519.78−706.49)	43.06 (16.71−70.02)
LICs	2.89 (2.50−3.29)	9.89 (8.57−11.20)	241.50 (194.54−289.31)	384.87 (330.05−439.69)	760.50 (658.59−862.38)	97.60 (67.93−127.84)		405.80 (349.83−461.77)	786.40 (682.00−890.76)	93.79 (64.98−123.15)

U-MICs. Upper-middle-income countries; L-MICs. Low-middle-income countries; LICs. Low-income countries; UI. Uncertainty interval

The number of secondary epilepsies due to different causes was estimated at 6625.33 thousand (95% UI 5441.67−7810.03) for neglected tropical diseases and malaria, 416.68 thousand (95% UI 343.76−489.56) for other infectious diseases, and 38,467.23 thousand (95% UI 32,964.03−43,976.69) for neonatal disorders in 2050. The ASPRs of secondary epilepsy attributable to different causes were forecast at 69.52 per 100,000 (95% UI 57.59−81.45) for neglected tropical diseases and malaria, 5.00 per 100,000 (95% UI 4.14−5.86) for other infectious diseases, and 509.59 per 100,000 (95%UI 436.55−582.71) for neonatal disorders in LMICs. Neonatal disorders were projected to remain the leading cause of secondary epilepsy and to show the most pronounced growth of ASPR [65.76% (95% UI 34.85−104.29)] from 2023. In contrast, increases associated with neglected tropical diseases and malaria [2.10% (95% UI −21.09 to 32.89)] and other infectious diseases [4.49% (95% UI −18.65 to 34.92)] were modest (**Additional file 1:** Table S2**,** Fig. S2).

#### 3.1.2 Projections of epilepsy prevalence by income level and country

**Fig. 3:**
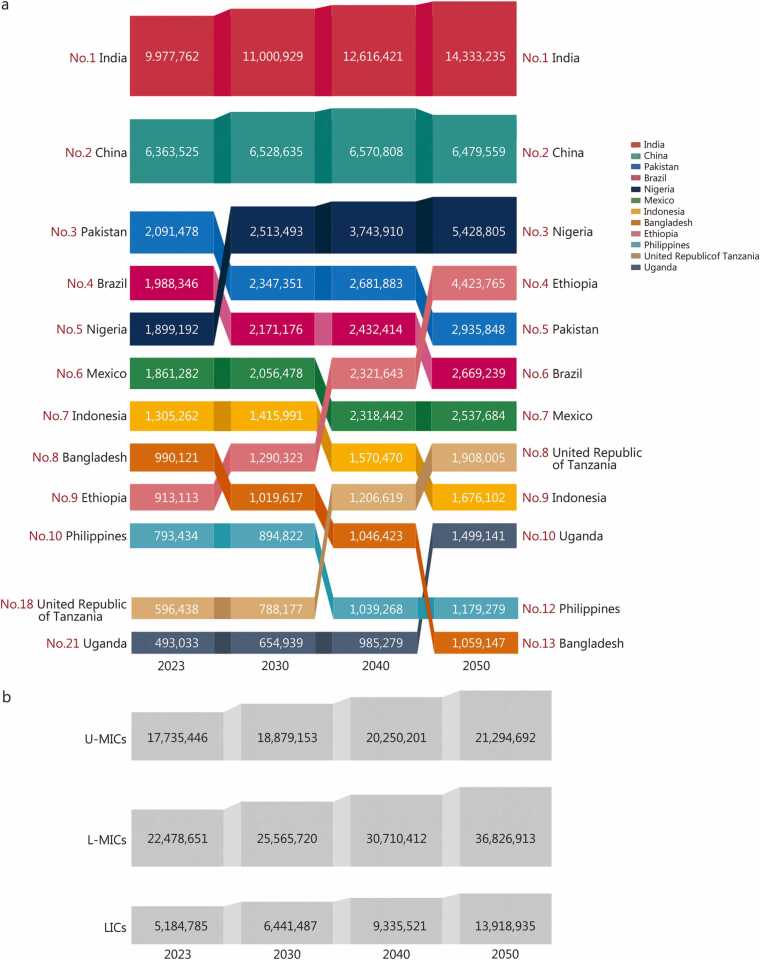
Projected top 10 low- and middle-income countries (LMICs) with the highest number of epilepsy (idiopathic and secondary) cases (**a**) and case counts (**b**) by income level in 2023, 2030, 2040, and 2050. U-MICs. Upper-middle-income countries; L-MICs. Low-middle-income countries; LICs. Low-income countries; UI. Uncertainty interval.

From 2023 to 2050, both the number of epilepsy cases and the ASPRs in LMICs were forecast to increase across income levels. The magnitude of the increase became greater as the income level declined. LICs were projected to experience the greatest rises, with the cases rising by 168.46% (95% UI 118.09−219.78) to 13.92 million (95% UI 11.48−16.36) and ASPR increasing by 54.02% (95% UI 20.54−88.29) to 1083.59 per 100,000 (95% UI 891.34−1275.69). Corresponding increases in upper-middle-income countries (U-MICs) and lower-middle-income countries (L-MICs) were 20.07% (95% UI −12.93 to 53.33) and 63.83% (95% UI 25.97−102.23) for the number of cases and 18.40% (95% UI −13.76 to 50.78) and 32.40% (95% UI −0.60 to 65.81) for ASPRs, respectively (Table 1**; Additional file 1:** Fig. S3). Among the 129 LMICs, 114 (88.37%) were estimated to show significant increases in ASPR, and 113 (87.59%) in case numbers from 2023 to 2050 (AAPC>0, *P*<0.001), with Ethiopia recording the highest AAPC for both (**Additional file 1:** Tables S1, S3). India was projected to have the largest number of epilepsy cases in 2050 [14,333.23 thousand (95% UI 11,450.65−17,227.23), followed by China [6479.56 thousand (95% UI 5127.36−7834.25). Nigeria was projected to rise from 5th in 2023 to 3rd in 2050, reaching 5428.80 thousand (95% UI 4424.38−6434.00), representing a 185.85% (95% UI 130.19−242.63) increase. Ethiopia was projected to move from 9th in 2023 to 4th in 2050, reaching 4423.76 thousand (95% UI 3720.04−5126.59), a 384.47% (95% UI 305.51−464.49) increase (Fig. 3**; Additional file 1:** Table S4**)**. By 2050, the highest ASPRs were projected in Ethiopia [2104.02 per 100,000 (95% UI 1766.61−2440.89)], followed by Uganda [1665.05 per 100,000 (95% UI 1371.27−1958.53)] and Angola [1646.13 per 100,000 (95% UI 1325.98−1966.74)], and the lowest in the Democratic People’s Republic of Korea [470.94 per 100,000 (95% UI 379.68−562.21)], the Syrian Arab Republic [497.89 per 100,000 (95% UI 395.74−600.22)], and Ukraine [507.20 per 100,000 (95% UI 408.42−605.93)]. Between 2023 and 2050, the greatest increases in ASPRs were also projected in LICs, including Ethiopia [184.94% (95% UI 135.78−235.04)], Rwanda [97.01% (95% UI 60.43−134.13)], and Mali [84.95% (95% UI 49.65−120.78)] (**Additional file 1:** Table S4**,** Fig. S4).

For idiopathic epilepsy, the projected increases in case numbers from 2023 to 2050 were considerably greater across all three income levels, ranging from 17.01% to 76.12%, than the corresponding increases in ASPRs, which ranged from 1.39% to 9.13%. Notably, LICs showed the largest increase in case numbers [76.12% (95% UI 20.21−132.70)] but the smallest increase in ASPR [1.39% (95% UI −38.25 to 41.02)] (Table 1**; Additional file 1:** Fig. S3). Overall, 116 (89.92%) LMICs were forecast to show significant rises in case numbers (AAPC>0, *P*<0.001), with the largest increase observed in Chad (AAPC=3.85, 95% CI 3.83−3.86) (**Additional file 1:** Tables S1, S3**)**. By 2050, India was projected to have the highest number of idiopathic epilepsy cases, at 4251.63 thousand (95% UI 3069.66−5438.20) (Fig. 3**, Additional file 1:** Table S4). The ASPRs were estimated to range from 170.59 per 100,000 (95% UI 123.14−218.09) in Yemen to 860.18 per 100,000 (95% UI 617.78−1101.86) in Mauritius (**Additional file 1:** Table S4**,** Fig. S4).

For secondary epilepsy, LICs were projected to experience the largest increases in both case numbers and ASPRs from 2023 to 2050, followed by L-MICs and U-MICs, indicating a progressive decline in growth with rising income levels. In LICs, the number of cases in LICs was estimated to reach 9.89 million (95% UI 8.57−11.20), reflecting a 241.50% (95% UI 194.54−289.31) increase, compared with 81.69% (95% UI 50.26−113.53) in L-MICs and 22.81% (95% UI −1.48 to 47.35) in U-MICs. The ASPR was forecast to reach 760.50 per 100,000 (95% UI 658.59−862.38) by 2050, representing a 97.60% (95% UI 67.93−127.84) increase from 2023, compared with 48.90% (95% UI 21.69−76.64) in L-MICs and 27.75% (95% UI 3.55−52.27) in U-MICs (Table 1**; Additional file 1:** Fig. S3). LICs were projected to experience the greatest increases in the ASPRs for all three secondary epilepsy causes. The ASPR of epilepsy attributable to neonatal disorders in LICs was projected to increase by 135.82% (95% UI 102.69−169.67), which was more than 33 times the increase associated with neglected tropical diseases and malaria [4.06% (95% UI −18.29 to 26.35)] and over 45 times that associated with other infectious diseases [3.03% (95% UI −19.18 to 25.18)] (**Additional file 1:** Table S2**,** Fig. S5). In addition, in both L-MICs and LICs, the ASPR of epilepsy due to neonatal sepsis and other neonatal infections was projected to surpass that due to cysticercosis by 2050 (**Additional file 1:** Fig. S6**)**. Among the 129 LMICs, 112 (86.82%) were projected to show significant increases in case numbers and 115 (89.15%) in ASPR from 2023 to 2050 (AAPC>0, *P*<0.001), with Ethiopia exhibiting the highest growth [AAPC of case number 7.38 (95% CI 7.35−7.41); AAPC of ASPR 5.37 (95% CI 5.29−5.44)] (**Additional file 1:** Tables S1, S3**)**. By 2050, India was forecast to have the largest number of cases [10081.60 thousand (95% UI 8380.99−11,789.04)], while the ASPRs were estimated to range from 258.30 per 100,000 (95% UI 220.69−295.89) in Libya to 1800.59 per 100,000 (95% UI 1547.54−2053.48) in Ethiopia (Fig. 3**; Additional file 1:** Table S4**,** Fig. S4).

#### 3.1.3 Demographic patterns of projected epilepsy prevalence

Age-specific prevalence patterns from 1990 to 2023 showed distinct distributions of epilepsy across age groups in LMICs (**Additional file 1:** Fig. S7). Our forecasts indicated an increased prevalence of idiopathic epilepsy in LMICs across all age groups by 2050 compared with 2023, with increases ranging from 3.01% to 9.81%. The prevalence of epilepsy attributable to the included secondary causes was projected to increase among individuals younger than 75 years, with particularly substantial growth (exceeding 50%) in those under 55, but to decline in individuals aged 75 years and older. Specifically, the prevalence of secondary epilepsy attributable to neglected tropical diseases and malaria was projected to rise in individuals under 50 but decline in those 50 and older. Epilepsy caused by other infectious diseases was expected to increase below age 70 and decrease in older populations. Epilepsy due to neonatal disorders was projected to increase across all ages, with the increase exceeding 80% in those aged 80 and above. By 2050, epilepsy attributable to the included secondary causes was projected to predominate in individuals younger than 70 years, whereas idiopathic epilepsy was expected to predominate in those aged 70 years and older in LMICs (**Additional file 1:** Table S5**,** Fig. S8).

In 2050, females were projected to account for 47.68% of all epilepsy cases (idiopathic and secondary) in LMICs, corresponding to 34,348.70 thousand cases (95% UI 27,518.74−41,187.26). Among these, 12,654.80 thousand (95% UI 9113.98−16,203.26) were projected to be idiopathic epilepsy, and 21,693.90 thousand (95% UI 18,404.76−24,984.00) were projected to be secondary epilepsy (**Additional file 1:** Table S6**,** Fig. S9**)**. The projected male-to-female ratio of the ASPR was 1.09 in 2023 and 1.10 in 2050 for idiopathic epilepsy. The corresponding projected ratios were 1.07 in 2023 and 1.10 in 2050 for secondary epilepsy overall, 1.14 in both 2023 and 2050 for epilepsy attributable to neonatal disorders, 0.80 in 2023 and 0.86 in 2050 for epilepsy attributable to neglected tropical diseases and malaria, and 1.02 in 2023 and 1.00 in 2050 for epilepsy attributable to other infectious diseases (**Additional file 1:** Figs. S1, S10).

### 3.2 Model validation

To evaluate model performance, we compared the predicted prevalence of epilepsy from 2011 to 2023 with the GBD 2023 reference data. The age- and sex-specific predictions maintained consistent accuracy across LMICs, as evidenced by RMSEs and MAEs below 0.001, MAPEs below 2.5%, and *R*^2^ values exceeding 0.9999, indicating excellent model fit. Further hindcasting using three temporal intervals demonstrated that our proposed model consistently outperformed the six original models, achieving markedly lower RMSEs across all intervals (**Additional file 1:** Tables S7, S8). In addition, the predicted 95% UIs consistently overlapped with the GBD 2023 reference estimates across 2011-2023, indicating good model calibration (**Additional file 1:** Fig. S11).

### 3.3 Decomposition analysis

**Fig. 4:**
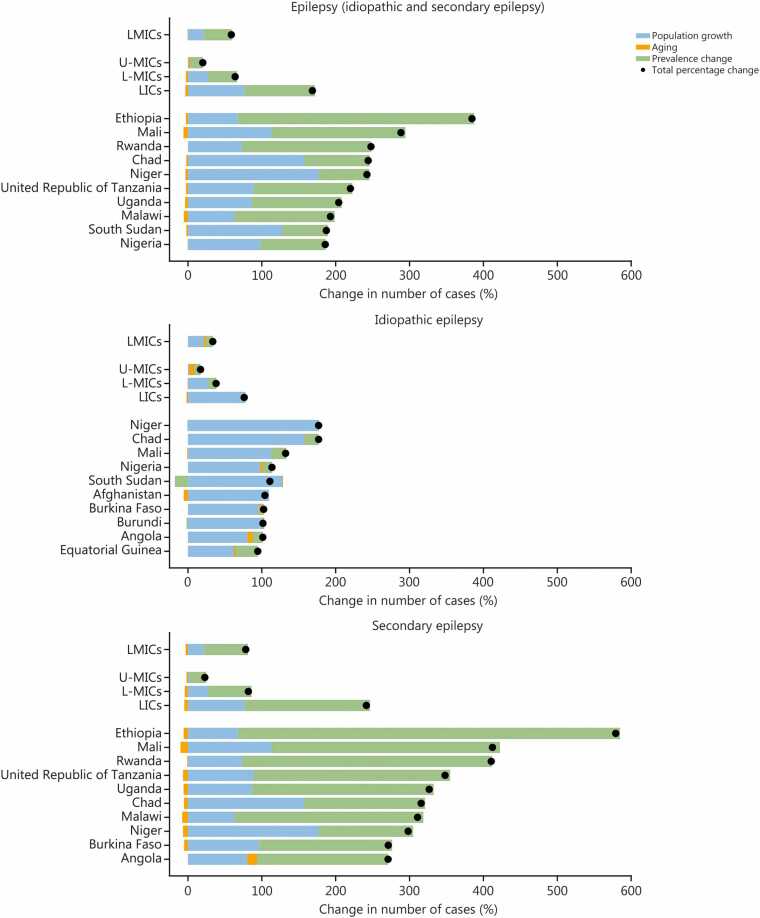
Decomposition of projected percentage changes in the number of cases with epilepsy (idiopathic and secondary), idiopathic epilepsy, and secondary epilepsy between 2023 and 2050 in low- and middle-income countries (LMICs), by income level and for the top 10 countries, both sexes. U-MICs. Upper-middle-income countries; L-MICs. Low-middle-income countries; LICs. Low-income countries.

In LMICs, prevalence change, population growth, and aging were projected to contribute 37.79%, 21.40%, and –0.51%, respectively, to the increase in epilepsy (idiopathic and secondary) cases from 2023 to 2050. For idiopathic epilepsy, the projected increase was driven primarily by population growth (21.40%), followed by prevalence change (9.39%) and aging (2.78%). In contrast, the rise in secondary epilepsy cases was driven predominantly by prevalence change (59.89%), with population growth contributing 21.40% and aging showing a negative effect (–3.06%) (Fig. 4**; Additional file 1:** Table S9).

The contributions of population growth, aging, and prevalence changes to increases in idiopathic epilepsy cases varied across income levels. In LICs and L-MICs, population growth was the predominant contributor (76.22% and 27.00%), followed by prevalence change (1.69% and 11.44%), whereas aging had a slight negative effect (–1.79% and –0.41%), consistent with the overall pattern observed across LMICs. However, in U-MICs, prevalence change and aging were the major contributors (8.56% and 6.86%), with a limited impact of population growth (1.59%). For secondary epilepsy, prevalence change remained the dominant driver across all three income levels, followed by population growth, whereas aging contributed negatively at all income levels. The contribution of prevalence change was highest in LICs (170.23%) and lowest in U-MICs (23.13%) (Fig. 4**; Additional file 1:** Table S9).

Among the 10 LMICs with the largest projected increases in idiopathic epilepsy cases, population growth was the main driver, ranging from 61.91% in Equatorial Guinea to 176.96% in Niger. In contrast, among the 10 countries with the largest projected increases in secondary epilepsy cases, prevalence change was the dominant contributor, ranging from 127.75% in Niger to 517.33% in Ethiopia (Fig. 4**; Additional file 1:** Table S9).

### 3.4 Projection of dementia-attributable secondary epilepsy prevalence in LMICs by 2050

The number of people living with dementia-attributable secondary epilepsy in LMICs was projected to reach 3.40 million (95% CI 0.16−6.64) by 2050, corresponding to an all-age prevalence of 42.06 per 100,000 (95% CI 1.99−82.13). Across income levels, U-MICs were expected to have the highest all-age prevalence at 81.69 per 100,000 (95% CI 3.87−159.51) and the largest number of cases, reaching 2.37 million (95% CI 0.11−4.62). L-MICs were projected to have 0.87 million (95% CI 0.04−1.69) cases with an all-age prevalence of 22.04 per 100,000 (95% CI 1.04−43.03), while LICs were estimated to have 0.17 million (95% CI 0.01−0.33) cases and an all-age prevalence of 13.45 per 100,000 (95% CI 0.64−26.26).

## 4 Discussion

This study provides comprehensive projections of the prevalence and number of epilepsy cases across 129 LMICs through 2050. We estimated an ASPR of 907.22 per 100,000 for epilepsy (idiopathic and secondary) in LMICs by 2050, representing a 33.32% increase from 2023, with prevalent cases reaching 72.04 million (58.68%). Among secondary epilepsy etiologies, the ASPR attributable to neonatal disorders was projected to increase by 65.76%, whereas ASPRs for other infectious diseases, neglected tropical diseases, and malaria were projected to increase by less than 5%. Decomposition analysis indicated that prevalence change, population growth, and aging contributed 37.79, 21.40, and −0.51%, respectively, to the projected increase in epilepsy cases in LMICs, with population growth primarily driving idiopathic epilepsy and prevalence change predominating in secondary epilepsy. Dementia-attributable secondary epilepsy cases were estimated to reach 3.40 million by 2050. These findings highlight the growing influence of preventable causes and demographic transitions on the future epilepsy burden in LMICs and underscore the need for prevention-oriented strategies, health system planning, and targeted resource allocation.

We projected a continued increase in both the prevalence and number of epilepsy cases in LMICs from 2023 to 2050, with the rise in secondary epilepsy markedly exceeding that of idiopathic epilepsy. Furthermore, the rise in the ASPR of secondary epilepsy was projected to exceed that observed between 1990 and 2023, whereas idiopathic epilepsy was expected to follow a similar trajectory to past trends. These divergent trends may reflect the increasing contribution of acquired causes of epilepsy and support the importance of preventive strategies targeting modifiable risk factors. Indeed, up to 25% of secondary epilepsy cases may be preventable through addressing their underlying causes, including traumatic brain injury (TBI), perinatal brain injury, stroke, and central nervous system infections [3]. In our projections, the increase in secondary epilepsy was primarily driven by neonatal disorders, which remained the leading and fastest-growing cause, while epilepsy associated with neglected tropical diseases and malaria, as well as other infectious diseases, were projected to exhibit modest increases.

For idiopathic epilepsy, despite minimal ASPR increase, LICs were projected to experience the largest rise in case numbers (76.12%), more than four times that in U-MICs (17.01%). Decomposition analysis revealed a distinct pattern compared with secondary epilepsy, with population growth as the dominant driver of case growth, while prevalence change accounted for only 1.69% of the increase. The greater rise in ASPR observed in middle-income countries may reflect better case identification facilitated by superior healthcare systems and diagnostic tools such as magnetic resonance imaging (MRI), electroencephalograms, and genetic testing [29], [30]. Modifiable risk factors of idiopathic epilepsy, including air pollution (e.g., fine particulate matter, ozone, nitrogen dioxide, and carbon monoxide) and alcohol use, which tend to be more common in middle-income countries than in LICs, may also play a role [31], [32], [33], [34], [35]. In LICs, socioeconomic development may exacerbate these factors and contribute to the rising prevalence of idiopathic epilepsy. However, due to limited data, the population attributable fraction for alcohol could not be estimated, highlighting a research gap [36]. These findings emphasize the need for region-specific prevention strategies, particularly in LICs.

Our projections revealed marked disparities in secondary epilepsy across income levels. Both prevalence and case numbers were projected to increase most sharply in LICs, with growth decreasing as income levels increase. The percentage increase of ASPR in LICs was estimated to be more than three times, and case numbers over ten times, those of U-MICs, respectively. Decomposition analysis indicated that prevalence change contributed substantially more to case growth in LICs, with prevalence change and population growth accounting for 170.23% and 76.22% of the increase, respectively, highlighting the combined impact of rising disease risk and demographic expansion. The increase in secondary epilepsy in LICs was primarily driven by neonatal disorders. Poverty, inadequate prenatal and perinatal care, and under-resourced health systems contribute to higher rates of preterm birth and hypoxic-ischemic injury in LMICs [37], [38]. A previous study reported a 39% lower risk of neonatal mortality among women in Sub-Saharan Africa who attended at least one antenatal assessment [39]. Strengthening maternal and neonatal care, including improved antenatal care, facility-based delivery, and timely interventions such as therapeutic hypothermia and comprehensive neonatal support, will be critical to mitigating this trend [40].

Our estimates indicated a continued rise in idiopathic epilepsy prevalence across all age groups in LMICs from 2023 to 2050. Secondary epilepsy prevalence was also projected to increase across most age groups, although a decline was anticipated in individuals aged 75 and above. However, this age-related decline should be interpreted cautiously, as major age-related causes of secondary epilepsy, including stroke, neurodegenerative diseases, and brain trauma, are not captured in the GBD 2023 estimates. In older adults, common etiologies of secondary epilepsy include stroke and other cerebrovascular diseases (accounting for 30% to 50% of cases), and neurodegenerative disorders (accounting for approximately 10% to 20% of cases) [41], [42]. In addition, older adults face a higher risk of TBI due to falls [43]. Reliance on GBD 2023 data alone is therefore likely to underestimate the future burden of secondary epilepsy in aging populations. Given accelerating global population aging, these findings highlight the need for more comprehensive evaluation and forward planning for epilepsy care, particularly for epilepsy attributable to stroke, TBI, and neurodegenerative diseases.

Meanwhile, we projected the male-to-female ratio of age-standardized idiopathic epilepsy prevalence to remain stable through 2050, whereas for secondary epilepsy, it was expected to rise mildly from 2023 to 2050. The sex differences in epilepsy susceptibility may be attributed to differences in body weight, steroid hormones, neurotransmitter systems, and neuronal networks [44]. However, studies on sex differences in secondary epilepsy remain limited and have yielded inconsistent results [45]. Further population-based studies are needed to clarify sex differences in epilepsy etiology and develop sex-specific prevention strategies.

Our study provides a systematic forecast of epilepsy prevalence by etiology in LMICs, addressing a significant gap in the literature, as previous projections have been scarce and limited to specific populations [46]. We developed a hybrid modeling framework integrating DNN and Transformer architectures with Poisson regression and ARIMA models. The DNN component enables flexible weighting of model outputs to capture nonlinear relationships, while the Transformer module models temporal dependencies using self-attention mechanisms. This integrative approach, combining established statistical methods with advanced deep learning techniques, yielded an excellent model fit during validation, suggesting its potential for robust and reliable epilepsy prevalence forecasting.

The current GBD 2023 framework captures only a subset of secondary epilepsy etiologies, while several major causes, including TBI, stroke, neurodegenerative diseases, and brain tumors, are not represented in the prevalence estimates [4]. Accurately forecasting secondary epilepsy related to these causes remains challenging. For instance, the prevalence of post-traumatic epilepsy varies markedly by injury severity, ranging from approximately 4% after mild TBI to 16% after severe TBI [47]. Moreover, cumulative incidence varies widely by injury severity and follow-up duration, with estimates of about 4% within 10 years after any TBI, and up to 25% at 5 years and 32% at 15 years following severe TBI [48], [49]. Similarly, although the prevalence of stroke can be projected from the GBD 2023 data, the incidence of post-stroke epilepsy shows considerable variation, ranging from 2% to 14% following ischemic stroke and 10% to 20% following hemorrhagic stroke [50], making post-stroke epilepsy prevalence difficult to estimate. Meanwhile, projected increases in neurodegenerative diseases, particularly in low SDI quintile, suggest a growing contribution of dementia and Parkinson’s disease to epilepsy burden by 2050 [7], [8]. To partially address this gap, we conducted an independent projection of dementia-attributable secondary epilepsy and estimated 3.40 million cases in LMICs by 2050, although projections for other major etiologies remain limited by data availability. These findings underscore the need for more comprehensive etiological modelling to better inform targeted prevention strategies in LMICs.

While our projections were based on GBD 2023 data, future trends may be influenced by the evolution of diagnostic approaches, alterations in diagnostic criteria, and advancements in therapeutic interventions. Overdiagnosis has been reported in 20.2% of epilepsy cases, with diagnostic delays ranging from 16% to 77% [51], [52]. In LICs, studies have indicated a diagnostic gap of 38.0% to 61.7% [53]. Emerging technologies, including diagnostic video tools, telehealth services, mobile EEG with remote interpretation, and online training, have been implemented to improve diagnostic accessibility and accuracy [54]. Scaling up these diagnostic innovations can reduce underdiagnosis, improve etiologic classification, and should be incorporated into long-term infrastructure planning. Meanwhile, the revised epilepsy definition proposed by the ILAE in 2014 may broaden the diagnostic threshold, potentially influencing diagnostic rates, treatment decisions, and, consequently, prevalence estimates in epidemiological studies [55], [56]. Additionally, recent advances in epilepsy therapeutics include over 200 pharmacological agents in preclinical or clinical development, alongside ongoing research into surgical and brain stimulation approaches [57], [58], [59]. These developments underscore the need for continuous updates to prevalence estimates for accurate public health planning.

Our study has certain limitations. First, the projection model may inherit limitations related to the quality and availability of existing data. Although the DNN framework partially mitigates data sparsity, epilepsy prevalence in LICs may remain underestimated due to stigma and underdiagnosis [4], [60]. As these challenges are generally more pronounced in LICs than in middle-income countries, the projected burden in LICs may be conservatively estimated, potentially leading to an underestimation of the true disparity in epilepsy burden between income levels. Second, external validation was not feasible, as no epilepsy prevalence projection studies based on non-GBD data are currently available, and existing non-GBD studies provide only cross-sectional estimates or cumulative cohort projections rather than annual prevalence data in high-income countries [61], [62]. Third, the lack of data on the etiologies of secondary epilepsy, such as stroke, neurodegenerative disorders, TBI, and brain tumors, limits comprehensive assessment of secondary epilepsy, particularly in older adults. Consequently, our estimates represent a partial etiologic subset within the GBD 2023 framework, and the reported growth rates, proportions, and geographic rankings should not be interpreted as comprehensive estimates of all-cause secondary epilepsy. Although we estimated dementia-attributable epilepsy, projections for other causes were not feasible due to insufficient epidemiological data on their future prevalence and prevalence of epilepsy in these conditions. In addition, the dementia-attributable epilepsy estimates relied on a pooled prevalence obtained from a limited evidence base with high heterogeneity (*I*^2^=99.9%). A simplifying assumption of constant prevalence across regions and over time was applied due to limited region-specific data, which may not fully capture geographic variation and introduces uncertainty into the estimated burden. Fourth, restricted access to MRI in LMICs may lead to misclassification of secondary epilepsy as idiopathic [63], and regional heterogeneity in diagnostic and reporting capacity may introduce detection bias, warranting cautious interpretation of stratified results. Systematic etiological screening with epilepsy-specific MRI sequences is essential to identify causes and reduce epilepsy prevalence through targeted interventions [64]. Fifth, incidence, remission, and mortality were not explicitly incorporated into our projection model. GBD 2021 estimates identified inconsistencies between epilepsy mortality and prevalence and highlighted uncertainty and variability in incidence, remission, and mortality estimates, particularly in LICs where data remain limited [4]. Sixth, while high alcohol use is a recognized risk factor for idiopathic epilepsy, population attributable fractions and potential impact fractions could not be estimated due to limited data [36]. Finally, estimates of prevalent cases may vary across population projection sources. Although estimates of the number of epilepsy (idiopathic and secondary) cases in LMICs in 2050 based on the 2024 Revision of the United Nations World Population Prospects (https://population.un.org/wpp/) [74.29 million (95% CI 59.70−88.90)] were highly consistent with those derived from GBD 2017 [72.04 million (95% CI 57.86−86.23)], the absence of explicit demographic scenario ranges suggests that the reported 95% UIs may not fully capture structural uncertainty related to future population dynamics. Furthermore, for long-term projections to 2050, assumptions regarding population growth, age structure, and epidemiological transitions constitute major sources of uncertainty and may partly account for the large projected increases observed in certain regions, particularly in LICs.

## 5 Conclusions

The prevalence of epilepsy was projected to increase in LMICs by 2050, with the most substantial growth in LICs. Secondary epilepsy was estimated to increase more rapidly than idiopathic epilepsy, with larger increases at lower income levels. From 2023 to 2050, population growth was projected to be the main driver of increased idiopathic epilepsy cases, whereas prevalence changes predominantly drove the rise in secondary epilepsy. These findings underscore the need for improved public awareness and region-specific interventions to address persistent diagnostic and treatment gaps, particularly in resource-limited settings. In the context of population aging and the growing contribution of neurodegenerative diseases, more comprehensive modeling of additional secondary epilepsy causes will be essential for accurately characterizing future disease burden and guiding prevention and management strategies.

## Abbreviations

AAPC: Average annual percentage change

ARIMA: Autoregressive Integrated Moving Average

ASPR: Age-standardized prevalence rate

CI: Confidence interval

DNN: Deep neural network

GBD: Global Burden of Disease

ICD: International Classification of Diseases

ILAE: International League Against Epilepsy

LICs: Low-income countries

L-MICs: Lower-middle-income countries

LMIC: Low- and middle-income countries

MRI: Magnetic resonance imaging

RMSE: Root mean squared error

SDI: Socio-Demographic Index

TBI: Traumatic brain injury

UI: Uncertainty interval

U-MICs: Upper-middle-income countries

## Ethics approval and consent to participate

Not applicable.

## Funding

This work was supported by the National Natural Science Foundation of China (82271459; 82571426) and the Beijing High-level Innovative and Entrepreneurial Talent Support Program (G202512038).

## Data Availability

The data used in this study are available via the Global Health Data Exchange query tool (https://ghdx.healthdata.org/).

## Footnotes

Appendix A Supplementary data associated with this article can be found in the online version at doi:10.1016/j.mmr.2026.100055.
