# Supplementary material for: Forecasting the prevalence of epilepsy in low- and middle-income countries to 2050 using a hybrid deep neural network-transformer modeling framework: insights from the Global Burden of Disease Study 2023
Source: Mil Med Res. 2026 Jul 23;13(1):100055. doi: 10.1016/j.mmr.2026.100055 (PMC13427421; doi:10.1016/j.mmr.2026.100055)
Supplement: Supplementary file 1 — Supplementary material [file mmc1.pdf]

Methods

Forecasting of epilepsy prevalence to 2050

In step 1, we constructed three original prevalence projection models using Poisson regression.

Model 1 accounted for differences across locations:

$$E[\log(Y_{l,a,s,y})]=\beta_1\ SDI_{l,y}+\alpha_{l,a,s}$$

Model 2 addressed variations among age groups:

$$E[\log(Y_{l,a,s,y})]=\beta_1\ SDI_y+\sum_{a=1}^{20}\beta_a\times\text{age group}_a+\alpha_{l,a,s}$$

Model 3 incorporated both locations and age groups differences:

$$E[\log(Y_{l,a,s,y})]=\beta_1\ SDI_{l,y}+\sum_{a=1}^{20}\beta_a\times\text{age group}_a+\alpha_{l,a,s}$$

For each combination of location (l), age group (a), sex (s), and year (y), we log-transformed the projected epilepsy prevalence as log ($Y_{l,a,s,y}$). β_1 was the fixed coefficient for the projected SDI, β_a referred to the fixed coefficient for different age groups, and $\alpha_{l,\ a,s}$ was the location-age-sex-specific random intercept.

In step 2, we generated three new models by fitting a random walk model [ARIMA (0, 1, 0)] to the residuals of the three original Poisson regression models to address the residual trends not elucidated by the primary covariates. ARIMA (0, 1, 0) was selected as a first-order random walk to capture smooth, non-stationary long-term trends in disease prevalence while minimizing overfitting in relatively short time series. This approach assumes gradual temporal evolution of unexplained residuals, which is consistent with long-term epidemiological dynamics. Models 4–6 shared the same mean structures as Models 1–3, respectively, but incorporated ARIMA-based residual forecasts to adjust the original projections.

Model 4 accounted for differences across locations:

$$E[\log(Y_{l,a,s,y})]=\beta_1\ SDI_{l,y}+\alpha_{l,a,s}$$

Model 5 addressed variations among age groups:

$$E[\log(Y_{l,a,s,y})]=\beta_1\ SDI_y+\sum_{a=1}^{20}\beta_a\times\text{age group}_a+\alpha_{l,a,s}$$

Model 6 incorporated both locations and age groups differences:

$$E[\log(Y_{l,a,s,y})] = \beta_1 \ SDI_{l,y} + \sum_{a=1}^{20} \beta_a \times \text{age group}_a + \alpha_{l,a,s}$$

In step 3, we adopted a deep learning approach to learn data-driven, time-varying weights for the six original models from steps 1 and 2. The DNN-Transformer architecture jointly determines the contribution of each model in the final prediction by integrating nonlinear fusion of model outputs with attention-based modeling of historical temporal patterns, enabling adaptive model weighting over time.

The DNN learned the nonlinear relationships between the prediction results of different models, thereby more accurately fusing the outputs of multiple models. To explicitly incorporate the temporal relationships between the prediction results and historical results, the Transformer module modeled the dynamic relationships among the performances of different years in the historical time series using self-attention mechanisms. This allowed it to capture long-range temporal dependencies and improve the temporal rationality of weight allocation. Finally, the outputs from the DNN and Transformer were summed to obtain the final prediction result. We denoted the prediction results of six models for year t as $X_1^t, X_2^t, \ldots, X_6^t$. In the DNN, these six models were first concatenated, which could be represented as:

$$\mathbf{X}_{\text{concat}}^t = [\mathbf{X}_1^t, \mathbf{X}_2^t, \ldots, \mathbf{X}_6^t]$$

Subsequently, the concatenated vector was passed through three fully connected (FC) layers to produce the final output y_{DNN}^t, which represented the prevalence rate for year t , denoted as:

$$\mathbf{Z}_1^t = \sigma(\mathbf{W}_1 \cdot \mathbf{X}_{\text{concat}}^t + \mathbf{b}_1) \in \mathbb{R}^{B \times 64}$$
$$\mathbf{Z}_2^t = \sigma(\mathbf{W}_2 \cdot \mathbf{Z}_1^t + \mathbf{b}_2) \in \mathbb{R}^{B \times 32}$$
$$y_{\text{DNN}}^t = \mathbf{W}_3 \cdot \mathbf{Z}_2^t + \mathbf{b}_3 \in \mathbb{R}^{B \times 1}$$

here, σ denoted the Rectified Linear Unit (ReLU) activation function, while $\mathbf{W}$ and $\mathbf{b}$ represented the weight and bias matrices, respectively.

In the Transformer module, the actual predicted values from the past k years were denoted as $X^{t-k}, X^{t-k+1}, \ldots, X^{t-1}$. These values were concatenated and passed through a linear projection layer, which mapped the input to a higher-dimensional space, formulated as:

$$\mathbf{X}_{\text{input}} = [X^{t-k}, X^{t-k+1}, \ldots, X^{t-1}]$$

$$\mathbf{Z}_0 = \mathbf{W}_p \cdot \mathbf{X}_{\text{input}} + \mathbf{b}_p$$

in which $\mathbf{W}$ and $\mathbf{b}$ denoted the weight and bias matrices, respectively. The positional encoding was then added to $\mathbf{Z}_0$, followed by a LayerNorm operation for normalization:

$$\mathbf{Z}_1 = \text{LayerNorm}(\mathbf{Z}_0 + \mathbf{P}_{\text{expanded}})$$

through the Transformer encoder, the final token was extracted as the predictive feature:

$$\mathbf{Z}_2 = \text{TransformerEncoder}(\mathbf{Z}_1)$$

$$\mathbf{Z}_3 = \mathbf{Z}_2[:, -1, :]$$

next, a multi-layer perceptron (MLP) was applied to obtain the final prediction result y^t_{Trans}:

$$\begin{gathered} \mathbf{Z}_4 = \sigma(\mathbf{W}_1 \cdot \mathbf{Z}_3 + \mathbf{b}_1) \\ \mathbf{Z}_5 = \sigma(\mathbf{W}_2 \cdot \mathbf{Z}_4 + \mathbf{b}_2) \\ y^t_{Trans} = \mathbf{W}_3 \cdot \mathbf{Z}_5 + \mathbf{b}_3 \in \mathbb{R}^{B \times 1} \end{gathered}$$

The final predicted prevalence in year *t*, denoted as $\hat{y}_t$, could be expressed as:

$$\hat{y}_t = y^t_{\text{DNN}} + \lambda \cdot y^t_{Trans}$$

λ was a hyperparameter that represents the weight of the subcomponent prediction results.

The loss function of the model was defined as the Root Mean Squared Error (RMSE) between the predicted prevalence and the ground-truth prevalence, formulated as:

$$\mathcal{L}_{\text{RMSE}} = \sqrt{\frac{1}{N}\sum_{i=1}^{N} (\hat{y}_t - y_t)^2}$$

We performed inference using the trained models to obtain epilepsy prevalence rates stratified by geographic location, age group, sex, and year.

We conducted the training on an Nvidia 4090 GPU, where the model was trained for 30,000 epochs with a batch size of 4. When t=1990, the hyperparameter λ was set to 0, and the input to the Transformer was noise data. For t >1990, λ was set to 0.2. The model was trained using historical data from 1990 to 2010 and validated on data from 2011 to 2023. To further assess the predictive performance of our model, we calculated the RMSE between the predicted prevalence and the GBD 2023 reference data for the six original models and our proposed model. In addition, we performed

multi-decade hindcasts using several training and validation intervals to evaluate the model's robustness under different historical periods. Specifically, three temporal configurations were used for hindcasting: [1, 1990–2006 (training) vs. 2007–2023 (validation); 2, 1990–2010 (training) vs. 2011–2023 (validation); and 3, 1990–2014 (training) vs. 2015–2023 (validation)].

Compilation of results

We systematically projected the prevalence rates of idiopathic epilepsy and 11 causes of secondary epilepsy for both sexes, across 20 age groups, and in 129 LMICs from 2024 to 2050. Uncertainty was propagated throughout all stages of the analysis by generating 1000 Monte Carlo draws for each prevalence estimate. These draws were derived from the intrinsic distributional assumptions of the underlying models. They were then carried forward through all intermediate modeling steps, including adjustments for measurement error, residual time-series modeling, and estimation of residual non-sampling error. All calculations were performed in a draw-by-draw manner, allowing uncertainty from multiple sources to be retained throughout the modeling pipeline. The 95% uncertainty intervals (UI) were defined as the 2.5th and 97.5th percentiles of the ordered 1000 draws for each projected prevalence estimate [1]. Interval calibration was evaluated by calculating the empirical coverage of predicted 95% UIs against GBD 2023 reference estimates for 2011–2023 (**Additional file 1: Fig. S11**). Empirical coverage was defined based on the degree of overlap between predicted and observed intervals and was examined across different income groups. To calculate the age-standardized prevalence, we employed a standard population derived from the non-weighted average across all countries of the percentage of the population in each 5-year age group from 2010 to 2035 [1]. The projected prevalence rates were multiplied by the projected population counts to estimate the number of cases. Countries were categorized into three World Bank income groups (upper-middle-income countries, lower-middle-income countries, and low-income countries) based on their Gross National Income per capita in 2023 [2].

Table S1 Average annual percent changes (AAPC) of age-standardized prevalence rate (ASPR) of epilepsy (idiopathic and secondary), idiopathic epilepsy, and secondary epilepsy from 1990 to 2023 and from 2023 to 2050 in low- and middle-income countries (LMICs), both sexes (95% CI)

Location	Epilepsy (idiopathic and secondary)				Idiopathic epilepsy				Secondary epilepsy			
	1990–2023	*P*-value	2023–2050	*P*-value	1990–2023	*P*-value	2023–2050	*P*-value	1990–2023	*P*-value	2023–2050	*P*-value
**LMICs**	0.40 (0.38−0.42)	<0.001	1.07 (1.06−1.08)	<0.001	0.26 (0.25−0.28)	<0.001	0.27 (0.27−0.28)	<0.001	0.51 (0.49−0.53)	<0.001	1.60 (1.59−1.62)	<0.001
**U-MICs**	0.37 (0.34−0.39)	<0.001	0.63 (0.63−0.63)	<0.001	0.22 (0.17−0.27)	<0.001	0.29 (0.28−0.29)	<0.001	0.49 (0.47−0.52)	<0.001	0.92 (0.91−0.92)	<0.001
Albania	0.04 (0.00−0.08)	0.078	0.16 (0.16−0.16)	<0.001	0.03 (−0.01 to 0.06)	0.188	0.11 (0.11−0.11)	<0.001	0.05 (0.02−0.09)	0.003	0.22 (0.22−0.22)	<0.001
Algeria	–0.04 (–0.07 to 0.00)	0.056	0.42 (0.41−0.42)	<0.001	–0.32 (–0.40 to –0.25)	<0.001	–0.07 (–0.07 to –0.07)	<0.001	0.34 (0.26−0.41)	<0.001	0.91 (0.90−0.91)	<0.001
Argentina	0.23 (0.21−0.25)	<0.001	0.29 (0.29−0.29)	<0.001	0.42 (0.40−0.43)	<0.001	0.27 (0.27−0.27)	<0.001	0.10 (0.08−0.11)	<0.001	0.30 (0.30−0.30)	<0.001
Armenia	0.02 (–0.06 to 0.11)	0.614	0.31 (0.31−0.31)	<0.001	–0.03 (–0.10 to 0.05)	0.478	0.07 (0.07−0.07)	<0.001	0.06 (–0.02 to 0.14)	0.139	0.46 (0.46−0.46)	<0.001
Azerbaijan	0.15 (0.09−0.21)	<0.001	0.25 (0.24−0.25)	<0.001	–0.27 (–0.36 to –0.19)	<0.001	0.07 (0.07−0.07)	<0.001	0.79 (0.74−0.84)	<0.001	0.45 (0.44−0.45)	<0.001
Belarus	–0.68 (–0.72 to –0.63)	<0.001	–0.13 (–0.13 to –0.13)	<0.001	–0.68 (–0.73 to –0.63)	<0.001	–0.21 (–0.22 to –0.21)	<0.001	–0.68 (–0.69 to –0.66)	<0.001	–0.06 (–0.07 to –0.06)	<0.001
Belize	0.09 (0.07−0.12)	<0.001	0.16 (0.16−0.16)	<0.001	0.11 (0.06−0.17)	<0.001	0.03 (0.03−0.04)	<0.001	0.07 (0.04−0.10)	<0.001	0.25 (0.24−0.25)	<0.001
Bosnia and Herzegovina	0.23 (0.15−0.30)	<0.001	0.28 (0.28−0.29)	<0.001	0.52 (0.42−0.62)	<0.001	0.30 (0.30−0.31)	<0.001	0.03 (–0.04 to 0.10)	0.407	0.27 (0.27−0.27)	<0.001
Botswana	–0.02 (–0.04 to 0.00)	0.069	0.54 (0.53−0.55)	<0.001	–0.13 (–0.18 to –0.09)	<0.001	–0.07 (–0.07 to –0.07)	<0.001	0.05 (0.01−0.09)	0.017	0.87 (0.87−0.88)	<0.001
Brazil	0.42 (0.37−0.46)	<0.001	0.74 (0.73−0.75)	<0.001	0.07 (0.05−0.10)	<0.001	0.05 (0.05−0.05)	<0.001	0.75 (0.63−0.87)	<0.001	1.24 (1.23−1.25)	<0.001
China	0.14 (0.05−0.24)	0.005	0.51 (0.50−0.51)	<0.001	–0.02 (–0.20 to 0.16)	0.798	0.02 (0.02−0.02)	<0.001	0.29 (0.24−0.33)	<0.001	0.86 (0.86−0.87)	<0.001
Colombia	0.18 (0.17−0.19)	<0.001	0.39 (0.39−0.40)	<0.001	0.23 (0.20−0.26)	<0.001	0.17 (0.17−0.17)	<0.001	0.12 (0.07−0.17)	<0.001	0.68 (0.67−0.68)	<0.001
Costa Rica	0.10 (0.03−0.17)	0.005	0.15 (0.15−0.15)	<0.001	0.43 (0.39−0.46)	<0.001	0.18 (0.18−0.18)	<0.001	–0.27 (–0.29 to –0.24)	<0.001	0.11 (0.11−0.12)	<0.001
Cuba	–0.07 (–0.09 to –0.05)	<0.001	0.32 (0.32−0.33)	<0.001	0.29 (0.25−0.33)	<0.001	0.52 (0.52−0.53)	<0.001	–0.41 (–0.42 to –0.39)	<0.001	0.11 (0.11−0.11)	<0.001
Dominica	0.06 (0.02−0.11)	0.004	0.07 (0.07−0.07)	<0.001	0.38 (0.28−0.48)	<0.001	0.13 (0.13−0.13)	<0.001	–0.24 (–0.26 to –0.21)	<0.001	0.01 (0.01−0.01)	<0.001
Dominican Republic	0.56 (0.52−0.60)	<0.001	0.30 (0.30−0.30)	<0.001	0.67 (0.62−0.72)	<0.001	0.38 (0.38−0.39)	<0.001	0.49 (0.46−0.51)	<0.001	0.25 (0.24−0.25)	<0.001

Location	Epilepsy (idiopathic and secondary)				Idiopathic epilepsy				Secondary epilepsy			
	**1990–2023**	***P*-value**	**2023–2050**	***P*-value**	**1990–2023**	***P*-value**	**2023–2050**	***P*-value**	**1990–2023**	***P*-value**	**2023–2050**	***P*-value**
Ecuador	–0.05 (–0.17 to 0.07)	0.446	0.16 (0.15−0.16)	<0.001	0.11 (−0.03 to 0.25)	0.126	0.11 (0.11−0.11)	<0.001	–0.30 (–0.39 to –0.21)	<0.001	0.23 (0.22−0.23)	<0.001
El Salvador	0.33 (0.26−0.40)	<0.001	0.19 (0.19−0.19)	<0.001	0.55 (0.50−0.59)	<0.001	0.21 (0.21−0.21)	<0.001	0.15 (0.13−0.18)	<0.001	0.17 (0.16−0.17)	<0.001
Equatorial Guinea	1.05 (0.92−1.18)	<0.001	0.82 (0.81−0.82)	<0.001	1.59 (1.36−1.82)	<0.001	0.62 (0.61−0.62)	<0.001	0.53 (0.43−0.63)	<0.001	1.05 (1.04−1.06)	<0.001
Fiji	0.10 (0.07−0.13)	<0.001	0.18 (0.18−0.18)	<0.001	0.20 (0.14−0.26)	<0.001	0.07 (0.07−0.07)	<0.001	−0.02 (−0.04 to 0.01)	0.156	0.31 (0.30−0.31)	<0.001
Gabon	−0.16 (−0.18 to −0.13)	<0.001	0.27 (0.27−0.27)	<0.001	−0.27 (−0.38 to −0.16)	<0.001	−0.06 (−0.06 to −0.06)	<0.001	−0.07 (−0.13 to −0.01)	0.035	0.60 (0.59−0.60)	<0.001
Georgia	0.17 (0.11−0.23)	<0.001	0.52 (0.52−0.53)	<0.001	0.13 (0.08−0.17)	<0.001	0.27 (0.27−0.28)	<0.001	0.22 (0.15−0.29)	<0.001	0.75 (0.74−0.75)	<0.001
Grenada	0.20 (0.17−0.23)	<0.001	0.10 (0.10−0.11)	<0.001	0.60 (0.52−0.69)	<0.001	0.20 (0.19−0.20)	<0.001	−0.12 (−0.14 to −0.11)	<0.001	0.02 (0.02−0.02)	<0.001
Guatemala	−0.01 (−0.03 to 0.01)	0.24	0.23 (0.23−0.24)	<0.001	0.17 (0.15−0.19)	<0.001	0.07 (0.07−0.07)	<0.001	−0.15 (−0.19 to −0.12)	<0.001	0.36 (0.35−0.36)	<0.001
Indonesia	1.24 (1.19−1.28)	<0.001	0.78 (0.78−0.79)	<0.001	0.39 (0.28−0.50)	<0.001	0.17 (0.17−0.17)	<0.001	2.21 (2.16−2.25)	<0.001	1.25 (1.24−1.27)	<0.001
Iran (Islamic Republic of)	0.78 (0.75−0.82)	<0.001	0.68 (0.67−0.69)	<0.001	0.13 (0.09−0.18)	<0.001	0.05 (0.05−0.05)	<0.001	1.78 (1.74−1.83)	<0.001	1.31 (1.31−1.32)	<0.001
Iraq	0.19 (0.14−0.25)	<0.001	0.13 (0.13−0.13)	<0.001	0.26 (0.21−0.32)	<0.001	0.11 (0.10−0.11)	<0.001	0.16 (0.11−0.20)	<0.001	0.14 (0.14−0.14)	<0.001
Jamaica	0.03 (−0.01 to 0.06)	0.116	0.07 (0.07−0.07)	<0.001	0.23 (0.19−0.28)	<0.001	0.07 (0.07−0.07)	<0.001	−0.12 (−0.15 to −0.09)	<0.001	0.08 (0.08−0.08)	<0.001
Kazakhstan	−0.06 (−0.11 to 0.00)	0.053	0.30 (0.30−0.30)	<0.001	−0.15 (−0.24 to −0.05)	0.003	0.04 (0.04−0.04)	<0.001	0.03 (0.00−0.06)	0.074	0.57 (0.57−0.58)	<0.001
Libya	0.17 (0.14−0.19)	<0.001	−0.01 (−0.02 to −0.01)	<0.001	0.30 (0.15−0.45)	<0.001	−0.18 (−0.22 to −0.13)	<0.001	0.05 (0.03−0.07)	<0.001	0.15 (0.14−0.15)	<0.001
Malaysia	0.14 (0.12−0.16)	<0.001	0.20 (0.20−0.20)	<0.001	0.40 (0.38−0.42)	<0.001	0.15 (0.15−0.16)	<0.001	−0.09 (−0.13 to −0.06)	<0.001	0.24 (0.24−0.24)	<0.001
Maldives	0.08 (0.02−0.13)	0.013	0.32 (0.32−0.33)	<0.001	0.31 (0.28−0.34)	<0.001	0.13 (0.13−0.13)	<0.001	−0.12 (−0.19 to −0.05)	0.001	0.50 (0.50−0.50)	<0.001
Marshall Islands	−0.13 (−0.16 to −0.10)	<0.001	0.07 (0.07−0.07)	<0.001	0.01 (−0.04 to 0.05)	0.711	−0.02 (−0.02 to −0.02)	<0.001	−0.24 (−0.25 to −0.22)	<0.001	0.14 (0.13−0.14)	<0.001
Mauritius	0.89 (0.84−0.94)	<0.001	0.56 (0.55−0.57)	<0.001	1.24 (1.13−1.35)	<0.001	0.46 (0.46−0.47)	<0.001	0.48 (0.45−0.51)	<0.001	0.68 (0.68−0.68)	<0.001
Mexico	0.02 (−0.01 to 0.05)	0.149	0.17 (0.17−0.17)	<0.001	−0.19 (−0.23 to −0.14)	<0.001	−0.01 (−0.02 to −0.01)	<0.001	0.27 (0.24−0.31)	<0.001	0.36 (0.36−0.37)	<0.001
Mongolia	0.54 (0.48−0.60)	<0.001	1.10 (1.09−1.11)	<0.001	0.32 (0.22−0.41)	<0.001	0.31 (0.31−0.31)	<0.001	0.70 (0.64−0.76)	<0.001	1.59 (1.58−1.61)	<0.001

Location	Epilepsy (idiopathic and secondary)				Idiopathic epilepsy				Secondary epilepsy			
	1990–2023	*P*-value	2023–2050	*P*-value	1990–2023	*P*-value	2023–2050	*P*-value	1990–2023	*P*-value	2023–2050	*P*-value
Montenegro	−0.19 (−0.21 to −0.17)	<0.001	−0.03 (−0.03 to −0.03)	<0.001	−0.11 (−0.15 to −0.07)	<0.001	0.04 (0.04−0.04)	<0.001	−0.25 (−0.28 to −0.23)	<0.001	−0.08 (−0.09 to −0.08)	<0.001
Namibia	0.39 (0.33−0.44)	<0.001	0.68 (0.67−0.69)	<0.001	0.33 (0.14−0.53)	0.001	0.20 (0.20−0.20)	<0.001	0.40 (0.36−0.44)	<0.001	0.98 (0.97−0.99)	<0.001
North Macedonia	−0.02 (−0.11 to 0.08)	0.717	0.05 (0.05−0.05)	<0.001	0.01 (−0.19 to 0.21)	0.903	0.06 (0.06−0.06)	<0.001	−0.07 (−0.08 to −0.05)	<0.001	0.04 (0.03−0.04)	<0.001
Paraguay	0.21 (0.20−0.23)	<0.001	0.15 (0.15−0.15)	<0.001	0.72 (0.69−0.76)	<0.001	0.32 (0.32−0.33)	<0.001	−0.18 (−0.21 to −0.16)	<0.001	−0.01 (−0.02 to −0.01)	<0.001
Peru	−0.36 (−0.43 to −0.29)	<0.001	0.18 (0.18−0.19)	<0.001	−0.43 (−0.51 to −0.34)	<0.001	−0.15 (−0.16 to −0.15)	<0.001	−0.32 (−0.35 to −0.28)	<0.001	0.51 (0.51−0.52)	<0.001
Republic of Moldova	−0.76 (−0.88 to −0.64)	<0.001	0.07 (0.06−0.07)	<0.001	−1.09 (−1.25 to −0.93)	<0.001	0.19 (0.17−0.20)	<0.001	−0.50 (−0.58 to −0.42)	<0.001	−0.02 (−0.03 to −0.02)	<0.001
Saint Lucia	−0.07 (−0.10 to −0.04)	<0.001	0.28 (0.28−0.28)	<0.001	0.30 (0.26−0.34)	<0.001	0.03 (0.03−0.03)	<0.001	−0.32 (−0.33 to −0.31)	<0.001	0.46 (0.46−0.47)	<0.001
Saint Vincent and the Grenadines	0.14 (0.12−0.16)	<0.001	0.18 (0.18−0.18)	<0.001	0.30 (0.23−0.36)	<0.001	0.10 (0.10−0.10)	<0.001	0.01 (0.00−0.02)	0.036	0.26 (0.25−0.26)	<0.001
Serbia	−0.23 (−0.24 to −0.21)	<0.001	0.00 (0.00−0.00)	<0.001	−0.22 (−0.26 to −0.17)	<0.001	−0.04 (−0.04 to −0.04)	<0.001	−0.24 (−0.25 to −0.23)	<0.001	0.03 (0.03−0.03)	<0.001
South Africa	0.17 (0.09−0.25)	<0.001	0.23 (0.23−0.23)	<0.001	−0.15 (−0.28 to −0.01)	0.031	−0.11 (−0.11 to −0.11)	<0.001	0.47 (0.40−0.55)	<0.001	0.46 (0.45−0.46)	<0.001
Suriname	0.14 (0.08−0.21)	<0.001	0.26 (0.26−0.26)	<0.001	0.24 (0.12−0.36)	<0.001	0.22 (0.22−0.22)	<0.001	0.03 (0.01−0.06)	0.005	0.29 (0.29−0.30)	<0.001
Thailand	0.34 (0.27−0.42)	<0.001	0.46 (0.46−0.47)	<0.001	0.58 (0.55−0.61)	<0.001	0.25 (0.25−0.26)	<0.001	0.13 (0.07−0.19)	<0.001	0.66 (0.66−0.66)	<0.001
Tonga	−0.04 (−0.06 to −0.02)	<0.001	0.07 (0.07−0.07)	<0.001	0.06 (0.04−0.07)	<0.001	−0.01 (−0.01 to −0.01)	<0.001	−0.12 (−0.14 to −0.11)	<0.001	0.15 (0.15−0.15)	<0.001
Turkey	0.22 (0.12−0.31)	<0.001	0.29 (0.29−0.30)	<0.001	0.54 (0.47−0.61)	<0.001	0.26 (0.25−0.26)	<0.001	−0.11 (−0.15 to −0.07)	<0.001	0.34 (0.34−0.34)	<0.001
Turkmenistan	0.82 (0.76−0.87)	<0.001	0.76 (0.75−0.77)	<0.001	0.78 (0.72−0.84)	<0.001	0.51 (0.50−0.51)	<0.001	0.93 (0.90−0.96)	<0.001	1.18 (1.17−1.19)	<0.001
Ukraine	−1.03 (−1.06 to −1.00)	<0.001	−0.05 (−0.05 to −0.05)	<0.001	−1.31 (−1.37 to −1.25)	<0.001	−0.19 (−0.20 to −0.18)	<0.001	−0.84 (−0.89 to −0.79)	<0.001	0.04 (0.04−0.04)	<0.001
**L-MICs**	0.34 (0.33−0.36)	<0.001	1.05 (1.03−1.06)	<0.001	0.36 (0.35−0.36)	<0.001	0.33 (0.32−0.33)	<0.001	0.33 (0.30−0.36)	<0.001	1.49 (1.47−1.51)	<0.001
Angola	0.33 (0.25−0.41)	<0.001	1.29 (1.27−1.31)	<0.001	0.30 (0.21−0.40)	<0.001	0.26 (0.25−0.26)	<0.001	0.34 (0.30−0.39)	<0.001	2.12 (2.09−2.15)	<0.001
Bangladesh	−0.30 (−0.35 to −0.25)	<0.001	0.47 (0.46−0.48)	<0.001	0.08 (0.02−0.14)	0.008	0.00 (0.00−0.00)	<0.001	−0.49 (−0.54 to −0.43)	<0.001	0.73 (0.72−0.74)	<0.001
Benin	0.21 (0.14−0.28)	<0.001	0.98 (0.97−1.00)	<0.001	0.04 (−0.09 to 0.18)	0.555	−0.07 (−0.07 to −0.07)	<0.001	0.38 (0.25−0.51)	<0.001	1.62 (1.59−1.64)	<0.001

Location	Epilepsy (idiopathic and secondary)				Idiopathic epilepsy				Secondary epilepsy			
	**1990–2023**	***P*-value**	**2023–2050**	***P*-value**	**1990–2023**	***P*-value**	**2023–2050**	***P*-value**	**1990–2023**	***P*-value**	**2023–2050**	***P*-value**
Bhutan	−0.05 (−0.07 to −0.03)	<0.001	0.43 (0.43−0.44)	<0.001	0.34 (0.30−0.37)	<0.001	0.14 (0.14−0.14)	<0.001	−0.26 (−0.28 to −0.25)	<0.001	0.60 (0.60−0.61)	<0.001
Bolivia (Plurinational State of)	−0.26 (−0.35 to −0.18)	<0.001	0.60 (0.58−0.61)	<0.001	−0.24 (−0.36 to −0.12)	<0.001	−0.20 (−0.20 to −0.19)	<0.001	−0.15 (−0.19 to −0.10)	<0.001	1.36 (1.34−1.38)	<0.001
Cabo Verde	0.33 (0.23−0.44)	<0.001	0.37 (0.36−0.37)	<0.001	0.54 (0.45−0.64)	<0.001	0.20 (0.20−0.20)	<0.001	0.18 (0.11−0.25)	<0.001	0.48 (0.48−0.49)	<0.001
Cambodia	0.29 (0.23−0.34)	<0.001	0.77 (0.76−0.78)	<0.001	0.47 (0.43−0.51)	<0.001	0.23 (0.23−0.23)	<0.001	0.17 (0.13−0.21)	<0.001	1.12 (1.11−1.13)	<0.001
Cameroon	0.16 (0.08−0.24)	<0.001	0.70 (0.70−0.71)	<0.001	0.08 (0.00−0.17)	0.057	0.00 (0.00−0.00)	<0.001	0.24 (0.15−0.33)	<0.001	1.24 (1.23−1.26)	<0.001
Comoros	0.71 (0.63−0.80)	<0.001	0.81 (0.80−0.82)	<0.001	−0.08 (−0.21 to 0.04)	0.181	−0.05 (−0.05 to −0.05)	<0.001	1.70 (1.62−1.78)	<0.001	1.46 (1.45−1.47)	<0.001
Congo	−0.07 (−0.10 to −0.04)	<0.001	1.00 (0.99−1.01)	<0.001	−0.30 (−0.35 to −0.25)	<0.001	−0.03 (−0.03 to −0.03)	<0.001	0.17 (0.10−0.23)	<0.001	1.74 (1.72−1.77)	<0.001
Côte d'Ivoire	0.31 (0.22−0.40)	<0.001	0.59 (0.59−0.60)	<0.001	0.14 (0.12−0.17)	<0.001	0.00 (0.00−0.00)	<0.001	0.43 (0.36−0.50)	<0.001	1.00 (0.98−1.02)	<0.001
Djibouti	0.74 (0.69−0.79)	<0.001	0.31 (0.30−0.32)	<0.001	−0.04 (−0.08 to 0.01)	0.099	−0.01 (−0.01 to −0.01)	<0.001	2.14 (2.08−2.21)	<0.001	0.69 (0.67−0.72)	<0.001
Egypt	0.35 (0.31−0.39)	<0.001	0.33 (0.33−0.33)	<0.001	0.58 (0.49−0.67)	<0.001	0.28 (0.28−0.28)	<0.001	0.20 (0.17−0.22)	<0.001	0.37 (0.36−0.37)	<0.001
Eswatini	0.33 (0.27−0.39)	<0.001	0.14 (0.14−0.15)	<0.001	0.28 (0.21−0.35)	<0.001	0.09 (0.09−0.09)	<0.001	0.36 (0.30−0.43)	<0.001	0.19 (0.19−0.19)	<0.001
Ghana	0.62 (0.59−0.64)	<0.001	0.76 (0.75−0.77)	<0.001	0.65 (0.61−0.68)	<0.001	0.23 (0.23−0.24)	<0.001	0.60 (0.54−0.67)	<0.001	1.12 (1.10−1.14)	<0.001
Guinea	0.70 (0.63−0.76)	<0.001	1.27 (1.25−1.29)	<0.001	0.31 (0.24−0.38)	<0.001	0.01 (0.01−0.01)	<0.001	1.29 (1.22−1.36)	<0.001	2.45 (2.43−2.48)	<0.001
Haiti	−0.43 (−0.47 to −0.39)	<0.001	0.05 (0.05−0.05)	<0.001	−0.21 (−0.24 to −0.19)	<0.001	−0.07 (−0.07 to −0.07)	<0.001	−0.57 (−0.64 to −0.51)	<0.001	0.12 (0.12−0.13)	<0.001
Honduras	−0.19 (−0.20 to −0.18)	<0.001	−0.04 (−0.04 to −0.03)	<0.001	−0.28 (−0.34 to −0.23)	<0.001	−0.07 (−0.08 to −0.07)	<0.001	−0.10 (−0.12 to −0.08)	<0.001	0.00 (−0.01 to 0.00)	0.067
India	0.29 (0.24−0.34)	<0.001	1.09 (1.08−1.11)	<0.001	0.43 (0.38−0.47)	<0.001	0.19 (0.19−0.19)	<0.001	0.22 (0.18−0.26)	<0.001	1.54 (1.52−1.55)	<0.001
Jordan	−0.27 (−0.29 to −0.25)	<0.001	0.39 (0.39−0.40)	<0.001	−0.40 (−0.45 to −0.35)	<0.001	−0.12 (−0.12 to −0.12)	<0.001	−0.17 (−0.20 to −0.14)	<0.001	0.74 (0.73−0.75)	<0.001
Kenya	0.44 (0.41−0.47)	<0.001	0.81 (0.80−0.82)	<0.001	0.12 (0.09−0.16)	<0.001	0.04 (0.04−0.04)	<0.001	0.73 (0.70−0.77)	<0.001	1.33 (1.31−1.35)	<0.001
Kiribati	−0.20 (−0.27 to −0.13)	<0.001	−0.02 (−0.02 to −0.01)	<0.001	−0.43 (−0.55 to −0.31)	<0.001	0.15 (0.14−0.16)	<0.001	0.02 (−0.02 to 0.04)	0.342	−0.17 (−0.18 to −0.16)	<0.001
Kyrgyzstan	−0.35 (−0.41 to −0.30)	<0.001	−0.09 (−0.10 to −0.08)	<0.001	−1.10 (−1.15 to −1.04)	<0.001	0.30 (0.29−0.32)	<0.001	0.65 (0.64−0.67)	<0.001	−0.51 (−0.54 to −0.49)	<0.001

Location	Epilepsy (idiopathic and secondary)				Idiopathic epilepsy				Secondary epilepsy			
	**1990–2023**	***P*-value**	**2023–2050**	***P*-value**	**1990–2023**	***P*-value**	**2023–2050**	***P*-value**	**1990–2023**	***P*-value**	**2023–2050**	***P*-value**
Lao People's Democratic Republic	0.47 (0.41–0.52)	<0.001	1.27 (1.25–1.29)	<0.001	0.73 (0.67–0.80)	<0.001	0.38 (0.37–0.38)	<0.001	0.27 (0.24–0.30)	<0.001	1.84 (1.82–1.86)	<0.001
Lebanon	−0.11 (−0.14 to −0.09)	<0.001	0.19 (0.19–0.20)	<0.001	0.03 (0.00–0.06)	0.037	0.04 (0.04–0.05)	<0.001	−0.29 (−0.31 to −0.28)	<0.001	0.37 (0.37–0.37)	<0.001
Lesotho	0.60 (0.56–0.64)	<0.001	0.17 (0.17–0.17)	<0.001	0.72 (0.69–0.75)	<0.001	0.31 (0.30–0.31)	<0.001	0.51 (0.47–0.56)	<0.001	0.07 (0.06–0.07)	<0.001
Mauritania	0.38 (0.37–0.40)	<0.001	1.00 (0.98–1.02)	<0.001	−0.10 (−0.15 to −0.06)	<0.001	−0.14 (−0.14 to −0.14)	<0.001	0.78 (0.70–0.85)	<0.001	1.62 (1.61–1.64)	<0.001
Micronesia (Federated States of)	−0.06 (−0.11 to −0.01)	0.022	0.12 (0.12–0.12)	<0.001	0.03 (−0.09 to 0.15)	0.655	0.01 (0.01–0.01)	<0.001	−0.14 (−0.16 to −0.13)	<0.001	0.21 (0.21–0.21)	<0.001
Morocco	0.03 (0.00–0.06)	0.045	0.18 (0.18–0.19)	<0.001	−0.04 (−0.07 to −0.02)	0.003	−0.01 (−0.01 to −0.01)	<0.001	0.08 (0.04–0.12)	<0.001	0.33 (0.33–0.33)	<0.001
Myanmar	0.71 (0.62–0.80)	<0.001	1.08 (1.06–1.09)	<0.001	0.79 (0.74–0.83)	<0.001	0.40 (0.40–0.41)	<0.001	0.63 (0.57–0.68)	<0.001	1.59 (1.56–1.61)	<0.001
Nepal	0.05 (0.02–0.09)	0.007	0.41 (0.40–0.41)	<0.001	0.55 (0.48–0.62)	<0.001	0.27 (0.26–0.27)	<0.001	−0.24 (−0.27 to −0.21)	<0.001	0.49 (0.49–0.50)	<0.001
Nicaragua	−0.09 (−0.14 to −0.03)	0.001	0.08 (0.08–0.08)	<0.001	0.07 (−0.02 to 0.16)	0.124	0.08 (0.08–0.08)	<0.001	−0.20 (−0.27 to −0.14)	<0.001	0.09 (0.09–0.09)	<0.001
Nigeria	0.89 (0.86–0.93)	<0.001	1.28 (1.27–1.30)	<0.001	0.42 (0.36–0.48)	<0.001	0.21 (0.21–0.21)	<0.001	1.45 (1.40–1.50)	<0.001	2.14 (2.12–2.17)	<0.001
Pakistan	0.31 (0.29–0.33)	<0.001	0.60 (0.59–0.61)	<0.001	−0.25 (−0.32 to −0.19)	<0.001	−0.08 (−0.08 to −0.08)	<0.001	0.85 (0.80–0.90)	<0.001	1.06 (1.05–1.07)	<0.001
Palestine	−0.03 (−0.06 to 0.01)	0.134	0.10 (0.10–0.10)	<0.001	0.05 (−0.01 to 0.10)	0.105	−0.07 (−0.07 to −0.07)	<0.001	−0.10 (−0.12 to −0.07)	<0.001	0.25 (0.25–0.25)	<0.001
Papua New Guinea	−0.16 (−0.18 to −0.15)	<0.001	0.24 (0.24–0.25)	<0.001	−0.11 (−0.15 to −0.06)	<0.001	−0.08 (−0.08 to −0.08)	<0.001	−0.19 (−0.22 to −0.16)	<0.001	0.45 (0.45–0.46)	<0.001
Philippines	0.61 (0.60–0.63)	<0.001	0.45 (0.45–0.45)	<0.001	0.18 (0.17–0.19)	<0.001	0.09 (0.09–0.09)	<0.001	1.03 (0.99–1.08)	<0.001	0.73 (0.72–0.74)	<0.001
Samoa	−0.08 (−0.12 to −0.03)	0.001	0.35 (0.34–0.35)	<0.001	0.15 (0.07–0.23)	<0.001	0.10 (0.10–0.10)	<0.001	−0.25 (−0.27 to −0.23)	<0.001	0.53 (0.52–0.53)	<0.001
São Tomé and Príncipe	0.71 (0.67–0.74)	<0.001	0.61 (0.60–0.62)	<0.001	0.54 (0.45–0.64)	<0.001	0.16 (0.16–0.16)	<0.001	0.82 (0.78–0.86)	<0.001	0.90 (0.89–0.91)	<0.001
Senegal	1.21 (1.18–1.23)	<0.001	1.69 (1.67–1.71)	<0.001	0.61 (0.49–0.73)	<0.001	0.04 (0.04–0.04)	<0.001	1.92 (1.79–2.05)	<0.001	2.82 (2.79–2.84)	<0.001
Solomon Islands	−0.06 (−0.07 to −0.05)	<0.001	0.00 (0.00–0.00)	0.76	0.20 (0.16–0.23)	<0.001	0.05 (0.05–0.05)	<0.001	−0.22 (−0.25 to −0.20)	<0.001	−0.04 (−0.04 to −0.03)	<0.001
Sri Lanka	−0.39 (−0.43 to −0.35)	<0.001	0.12 (0.12–0.12)	<0.001	−0.28 (−0.35 to −0.22)	<0.001	0.01 (0.01–0.01)	<0.001	−0.48 (−0.50 to −0.46)	<0.001	0.22 (0.22–0.22)	<0.001
Tajikistan	0.16 (0.07–0.25)	<0.001	−0.57 (−0.60 to −0.54)	<0.001	−0.26 (−0.32 to −0.19)	<0.001	−0.49 (−0.54 to −0.44)	<0.001	0.85 (0.78–0.91)	<0.001	−0.70 (−0.70 to −0.69)	<0.001

Location	Epilepsy (idiopathic and secondary)				Idiopathic epilepsy				Secondary epilepsy			
	1990–2023	*P*-value	2023–2050	*P*-value	1990–2023	*P*-value	2023–2050	*P*-value	1990–2023	*P*-value	2023–2050	*P*-value
Timor-Leste	0.15 (0.07−0.23)	<0.001	0.56 (0.56−0.57)	<0.001	0.31 (0.16−0.45)	<0.001	0.04 (0.03−0.04)	<0.001	0.01 (−0.02 to 0.05)	0.455	0.85 (0.84−0.86)	<0.001
Tunisia	−0.06 (−0.10 to −0.02)	0.003	0.16 (0.16−0.16)	<0.001	−0.17 (−0.30 to −0.05)	0.006	−0.06 (−0.06 to −0.06)	<0.001	0.09 (0.08−0.11)	<0.001	0.39 (0.39−0.40)	<0.001
United Republic of Tanzania	0.57 (0.49−0.65)	<0.001	1.95 (1.93−1.98)	<0.001	−0.05 (−0.20 to 0.11)	0.548	−0.01 (−0.01 to −0.01)	<0.001	1.48 (1.43−1.53)	<0.001	3.36 (3.32−3.39)	<0.001
Uzbekistan	0.38 (0.35−0.41)	<0.001	0.17 (0.17−0.17)	<0.001	0.05 (−0.01 to 0.10)	0.075	0.00 (0.00−0.00)	<0.001	0.91 (0.87−0.95)	<0.001	0.40 (0.40−0.41)	<0.001
Vanuatu	−0.05 (−0.06 to −0.04)	<0.001	0.15 (0.15−0.15)	<0.001	0.10 (0.09−0.11)	<0.001	0.02 (0.02−0.02)	<0.001	−0.16 (−0.19 to −0.12)	<0.001	0.24 (0.24−0.24)	<0.001
Viet Nam	0.23 (0.18−0.27)	<0.001	0.15 (0.15−0.15)	<0.001	0.64 (0.59−0.69)	<0.001	0.28 (0.28−0.28)	<0.001	−0.05 (−0.07 to −0.02)	0.001	0.06 (0.06−0.06)	<0.001
Zambia	0.21 (0.17−0.26)	<0.001	1.34 (1.32−1.36)	<0.001	−0.05 (−0.06 to −0.04)	<0.001	0.04 (0.04−0.04)	<0.001	0.61 (0.54−0.67)	<0.001	2.61 (2.58−2.65)	<0.001
Zimbabwe	0.23 (0.15−0.30)	<0.001	0.00 (−0.02 to 0.01)	0.672	0.23 (0.09−0.37)	0.001	0.00 (0.00−0.00)	0.731	0.25 (0.19−0.31)	<0.001	−0.01 (−0.03 to 0.02)	0.669
**LICs**	0.38 (0.36−0.40)	<0.001	1.61 (1.59−1.63)	<0.001	−0.05 (−0.07 to −0.03)	<0.001	0.05 (0.04−0.05)	<0.001	0.82 (0.77−0.86)	<0.001	2.56 (2.52−2.59)	<0.001
Afghanistan	−0.25 (−0.33 to −0.18)	<0.001	0.04 (0.04−0.04)	<0.001	−0.27 (−0.31 to −0.23)	<0.001	0.01 (0.01−0.01)	<0.001	−0.24 (−0.27 to −0.20)	<0.001	0.06 (0.06−0.07)	<0.001
Burkina Faso	0.26 (0.20−0.32)	<0.001	1.22 (1.20−1.24)	<0.001	0.32 (0.18−0.47)	<0.001	0.06 (0.06−0.06)	<0.001	0.21 (0.13−0.29)	<0.001	2.34 (2.31−2.37)	<0.001
Burundi	−0.28 (−0.35 to −0.21)	<0.001	0.08 (0.06−0.10)	<0.001	−1.13 (−1.19 to −1.07)	<0.001	−0.03 (−0.04 to −0.02)	<0.001	0.62 (0.56−0.69)	<0.001	0.17 (0.11−0.22)	<0.001
Central African Republic	−0.28 (−0.30 to −0.26)	<0.001	−0.01 (−0.02 to −0.01)	<0.001	−0.32 (−0.38 to −0.27)	<0.001	0.05 (0.04−0.05)	<0.001	−0.25 (−0.29 to −0.21)	<0.001	−0.07 (−0.07 to −0.06)	<0.001
Chad	0.49 (0.42−0.55)	<0.001	0.96 (0.95−0.97)	<0.001	0.35 (0.24−0.46)	<0.001	0.23 (0.23−0.23)	<0.001	0.69 (0.65−0.74)	<0.001	1.67 (1.65−1.68)	<0.001
Democratic People’s Republic of Korea	−0.46 (−0.58 to −0.35)	<0.001	0.04 (0.03−0.05)	<0.001	−0.81 (−0.84 to −0.79)	<0.001	−0.02 (−0.03 to −0.02)	<0.001	−0.21 (−0.27 to −0.16)	<0.001	0.08 (0.05−0.10)	<0.001
Democratic Republic of the Congo	0.14 (0.05−0.23)	0.003	−0.45 (−0.54 to −0.37)	<0.001	−0.13 (−0.16 to −0.09)	<0.001	−0.18 (−0.20 to −0.15)	<0.001	0.37 (0.29−0.45)	<0.001	−0.67 (−0.82 to −0.52)	<0.001
Eritrea	0.60 (0.51−0.68)	<0.001	1.40 (1.38−1.42)	<0.001	0.19 (0.05−0.32)	0.008	−0.01 (−0.01 to −0.01)	<0.001	1.06 (1.01−1.11)	<0.001	2.32 (2.29−2.36)	<0.001
Ethiopia	1.19 (1.14−1.24)	<0.001	3.96 (3.91−4.01)	<0.001	0.31 (0.23−0.39)	<0.001	0.06 (0.06−0.06)	<0.001	1.97 (1.85−2.08)	<0.001	5.37 (5.29−5.44)	<0.001
Gambia	0.78 (0.74−0.81)	<0.001	0.62 (0.61−0.63)	<0.001	0.17 (0.12−0.22)	<0.001	0.00 (0.00−0.00)	<0.001	1.25 (1.12−1.38)	<0.001	0.99 (0.99−1.00)	<0.001
Guinea-Bissau	0.35 (0.32−0.39)	<0.001	1.46 (1.44−1.48)	<0.001	−0.01 (−0.08 to 0.06)	0.835	−0.10 (−0.10 to −0.10)	<0.001	0.75 (0.71−0.79)	<0.001	2.52 (2.49−2.55)	<0.001

Location	Epilepsy (idiopathic and secondary)				Idiopathic epilepsy				Secondary epilepsy			
	**1990–2023**	***P*-value**	**2023–2050**	***P*-value**	**1990–2023**	***P*-value**	**2023–2050**	***P*-value**	**1990–2023**	***P*-value**	**2023–2050**	***P*-value**
Liberia	−0.30 (−0.42 to −0.17)	<0.001	−0.19 (−0.28 to −0.11)	<0.001	−1.10 (−1.28 to −0.92)	<0.001	0.20 (0.19−0.21)	<0.001	0.38 (0.32−0.43)	<0.001	−0.42 (−0.43 to −0.41)	<0.001
Madagascar	0.26 (0.22−0.31)	<0.001	0.47 (0.47−0.48)	<0.001	−0.53 (−0.55 to −0.51)	<0.001	−0.09 (−0.10 to −0.09)	<0.001	1.05 (0.92−1.19)	<0.001	0.85 (0.83−0.86)	<0.001
Malawi	0.50 (0.47−0.53)	<0.001	2.20 (2.17−2.24)	<0.001	−0.02 (−0.06 to 0.01)	0.207	−0.01 (−0.01 to −0.01)	<0.001	1.10 (1.02−1.18)	<0.001	3.55 (3.51−3.60)	<0.001
Mali	1.20 (1.11−1.29)	<0.001	2.31 (2.28−2.34)	<0.001	1.03 (0.88−1.17)	<0.001	0.33 (0.33−0.33)	<0.001	1.41 (1.27−1.54)	<0.001	3.55 (3.50−3.59)	<0.001
Mozambique	0.58 (0.48−0.69)	<0.001	0.85 (0.84−0.86)	<0.001	0.27 (0.07−0.46)	0.007	−0.03 (−0.03 to −0.03)	<0.001	0.94 (0.82−1.06)	<0.001	1.60 (1.58−1.62)	<0.001
Niger	0.62 (0.56−0.67)	<0.001	0.79 (0.78−0.80)	<0.001	−0.02 (−0.05 to 0.01)	0.155	−0.02 (−0.02 to −0.02)	<0.001	1.43 (1.36−1.50)	<0.001	1.47 (1.44−1.50)	<0.001
Rwanda	0.05 (−0.05 to 0.14)	0.325	2.55 (2.51−2.59)	<0.001	−0.52 (−0.66 to −0.39)	<0.001	−0.14 (−0.14 to −0.14)	<0.001	0.68 (0.66−0.71)	<0.001	4.04 (3.99−4.10)	<0.001
Sierra Leone	0.30 (0.23−0.38)	<0.001	0.71 (0.70−0.72)	<0.001	−0.06 (−0.16 to 0.04)	0.235	−0.02 (−0.02 to −0.02)	<0.001	1.02 (0.93−1.11)	<0.001	1.66 (1.64−1.67)	<0.001
Somalia	0.78 (0.68−0.88)	<0.001	1.35 (1.33−1.37)	<0.001	−0.09 (−0.14 to −0.04)	0.001	−0.06 (−0.06 to −0.06)	<0.001	2.01 (1.92−2.10)	<0.001	2.38 (2.35−2.41)	<0.001
South Sudan	−0.12 (−0.20 to −0.04)	0.004	0.84 (0.83−0.85)	<0.001	−0.91 (−0.96 to −0.87)	<0.001	−0.28 (−0.28 to −0.28)	<0.001	1.28 (1.13−1.43)	<0.001	1.89 (1.87−1.91)	<0.001
Sudan	0.43 (0.39−0.47)	<0.001	0.81 (0.80−0.83)	<0.001	0.08 (0.06−0.10)	<0.001	0.05 (0.05−0.05)	<0.001	0.73 (0.67−0.80)	<0.001	1.33 (1.32−1.35)	<0.001
Syrian Arab Republic	−0.31 (−0.35 to −0.27)	<0.001	−0.09 (−0.09 to −0.09)	<0.001	−0.33 (−0.37 to −0.29)	<0.001	−0.11 (−0.11 to −0.11)	<0.001	−0.28 (−0.37 to −0.19)	<0.001	−0.08 (−0.08 to −0.08)	<0.001
Togo	−0.39 (−0.47 to −0.30)	<0.001	0.26 (0.26−0.27)	<0.001	−0.74 (−0.88 to −0.61)	<0.001	−0.22 (−0.23 to −0.22)	<0.001	−0.14 (−0.22 to −0.05)	0.001	0.57 (0.57−0.58)	<0.001
Uganda	0.73 (0.63−0.83)	<0.001	1.81 (1.79−1.83)	<0.001	0.41 (0.26−0.57)	<0.001	0.15 (0.15−0.15)	<0.001	1.15 (1.05−1.25)	<0.001	3.12 (3.09−3.16)	<0.001
Yemen	−0.43 (−0.50 to −0.37)	<0.001	0.29 (0.28−0.29)	<0.001	−0.34 (−0.52 to −0.16)	<0.001	−0.07 (−0.07 to −0.07)	<0.001	−0.48 (−0.53 to −0.43)	<0.001	0.48 (0.47−0.48)	<0.001

U-MICs. Upper-middle-income countries; L-MICs. Low-middle-income countries; LICs. Low-income countries

Table S2 Projected number of cases, age-standardized prevalence rate (ASPR), and all age prevalence of secondary epilepsy caused by neglected tropical diseases and malaria, other infectious diseases, and neonatal disorders in low- and middle-income countries (LMICs) in 2023 and 2050, along with percent changes from 2023 to 2050, both sexes (95% UI)

Income level	Number of cases			ASPR			All age prevalence		
	2023 (thousand)	2050 (thousand)	Percent change (%)	2023 (per 100,000)	2050 (per 100,000)	Percent change (%)	2023 (per 100,000	2050 (per 100,000	Percent change (%)
**Neglected tropical diseases and malaria**									
LMICs	4123.25 (3350.96−4896.45)	6625.33 (5441.67−7810.03)	60.68 (23.86−109.08)	68.09 (55.17−81.01)	69.52 (57.59−81.45)	2.10 (−21.09 to 32.89)	61.87 (50.28−73.47)	81.89 (67.26−96.54)	32.36 (1.61−73.02)
U-MICs	2077.06 (1628.20−2526.71)	2691.36 (2124.44−3258.75)	29.58 (−4.84 to 64.59)	64.18 (50.30−78.08)	60.50 (47.68−73.33)	−5.73 (−35.06 to 23.73)	72.84 (57.10−88.61)	92.90 (73.33−112.49)	27.55 (−6.63 to 62.19)
L-MICs	1557.94 (1299.60−1816.35)	2838.16 (2377.52−3299.46)	82.17 (48.74−116.17)	65.99 (54.35−77.62)	67.36 (56.68−78.05)	2.08 (−21.72 to 26.06)	50.27 (41.93−58.60)	72.10 (60.40−83.82)	43.44 (15.35−72.11)
LICs	488.24 (423.16−553.38)	1095.80 (939.71−1251.83)	124.44 (90.34−159.21)	98.91 (83.41−114.41)	102.92 (87.06−118.78)	4.06 (−18.29 to 26.35)	68.45 (59.32−77.58)	87.17 (74.75−99.58)	27.36 (5.28−49.93)
**Other infectious diseases**									
LMICs	311.79 (256.59−367.07)	416.68 (343.76−489.56)	33.64 (3.85−71.80)	4.78 (3.93−5.64)	5.00 (4.14−5.86)	4.49 (−18.65 to 34.92)	4.68 (3.85−5.51)	5.15 (4.25−6.05)	10.09 (−14.96 to 41.94)
U-MICs	51.40 (41.40−61.39)	49.57 (39.96−59.17)	−3.56 (−30.45 to 23.38)	1.85 (1.49−2.21)	1.48 (1.19−1.77)	−20.27 (−44.95 to 4.59)	1.80 (1.45−2.15)	1.71 (1.38−2.04)	−5.07 (−31.72 to 21.69)
L-MICs	180.03 (147.26−212.89)	218.65 (178.41−258.87)	21.45 (−7.14 to 50.34)	6.48 (5.28−7.69)	5.54 (4.54−6.55)	−14.48 (−38.61 to 9.85)	5.81 (4.75−6.87)	5.55 (4.53−6.58)	−4.37 (−29.66 to 20.95)
LICs	80.36 (67.93−92.79)	148.46 (125.39−171.52)	84.75 (52.56−117.55)	12.64 (10.68−14.60)	13.02 (11.00−15.04)	3.03 (−19.18 to 25.18)	11.27 (9.52−13.01)	11.81 (9.97−13.64)	4.84 (−17.53 to 27.23)
**Neonatal disorders**									
LMICs	21099.23 (17990.72−24212.27)	38467.23 (32964.03−43976.69)	82.32 (48.35−124.88)	307.42 (262.18−352.74)	509.59 (436.55−582.71)	65.76 (34.85−104.29)	316.60 (269.96−363.31)	475.47 (407.45−543.57)	50.18 (22.16−84.76)
U-MICs	7229.38 (6239.70−8220.75)	8751.31 (7551.83−9951.53)	21.05 (−0.27 to 42.59)	264.94 (228.55−301.41)	360.83 (310.98−410.71)	36.19 (13.34−59.58)	253.52 (218.81−288.28)	302.08 (260.68−343.51)	19.16 (−1.97 to 40.46)
L-MICs	11543.74 (9746.62−13343.72)	21074.63 (17904.09−24251.05)	82.56 (51.35−114.17)	347.76 (293.69−401.93)	552.80 (469.54−636.22)	58.96 (30.72−87.62)	372.45 (314.47−430.53)	535.40 (454.85−616.09)	43.75 (17.60−70.50)
LICs	2326.11 (2004.39−2647.79)	8641.30 (7508.11−9774.11)	271.49 (221.35−322.45)	273.33 (235.97−310.68)	644.56 (560.54−728.56)	135.82 (102.69−169.67)	326.09 (280.99−371.18)	687.42 (597.27−777.53)	110.81 (80.28−141.84)

U-MICs. Upper-middle-income countries; L-MICs. Low-middle-income countries; LICs. Low-income countries

Table S3 Average annual percent changes (AAPC) of case number of epilepsy (idiopathic and secondary), idiopathic epilepsy, and secondary epilepsy from 1990 to 2023 and 2023 to 2050 in low- and middle-income countries (LMICs), both sexes (95% CI)

Location	Epilepsy (idiopathic and secondary)				Idiopathic epilepsy				Secondary epilepsy			
	1990–2023	*P*-value	2023–2050	*P*-value	1990–2023	*P*-value	2023–2050	*P*-value	1990–2023	*P*-value	2023–2050	*P*-value
**LMICs**	1.83 (1.82−1.84)	<0.001	1.73 (1.72−1.74)	<0.001	1.67 (1.65−1.68)	<0.001	1.08 (1.07−1.09)	<0.001	1.96 (1.94−1.98)	<0.001	2.17 (2.16−2.17)	<0.001
**U-MICs**	1.21 (1.19−1.22)	<0.001	0.69 (0.67−0.70)	<0.001	1.00 (0.95−1.05)	<0.001	0.58 (0.58−0.59)	<0.001	1.40 (1.37−1.43)	<0.001	0.77 (0.75−0.80)	<0.001
Albania	−0.63 (−0.99 to −0.26)	0.001	−0.04 (−0.05 to −0.03)	<0.001	−0.53 (−0.64 to −0.42)	<0.001	0.06 (0.05−0.08)	<0.001	−0.82 (−1.12 to −0.51)	<0.001	−0.18 (−0.19 to −0.17)	<0.001
Algeria	1.38 (1.35−1.41)	<0.001	1.39 (1.38−1.40)	<0.001	1.07 (1.01−1.12)	<0.001	1.09 (1.08−1.10)	<0.001	1.76 (1.68−1.83)	<0.001	1.70 (1.67−1.73)	<0.001
Argentina	1.30 (1.28−1.31)	<0.001	0.78 (0.77−0.80)	<0.001	1.51 (1.50−1.53)	<0.001	0.84 (0.82−0.85)	<0.001	1.15 (1.13−1.17)	<0.001	0.75 (0.73−0.76)	<0.001
Armenia	−0.35 (−0.43 to −0.27)	<0.001	−0.01 (−0.02 to 0.00)	0.196	−0.40 (−0.52 to −0.28)	<0.001	−0.08 (−0.09 to −0.08)	<0.001	−0.32 (−0.41 to −0.24)	<0.001	0.04 (0.03−0.06)	<0.001
Azerbaijan	1.18 (1.12−1.24)	<0.001	0.30 (0.29−0.31)	<0.001	0.81 (0.76−0.86)	<0.001	0.37 (0.36−0.37)	<0.001	1.70 (1.66−1.74)	<0.001	0.23 (0.22−0.24)	<0.001
Belarus	−0.93 (−0.97 to −0.89)	<0.001	−0.65 (−0.65 to −0.64)	<0.001	−0.84 (−0.88 to −0.80)	<0.001	−0.53 (−0.53 to −0.53)	<0.001	−0.98 (−1.01 to −0.96)	<0.001	−0.74 (−0.74 to −0.74)	<0.001
Belize	2.81 (2.75−2.86)	<0.001	1.29 (1.27−1.30)	<0.001	2.76 (2.68−2.84)	<0.001	1.23 (1.22−1.24)	<0.001	2.83 (2.81−2.85)	<0.001	1.32 (1.31−1.34)	<0.001
Bosnia and Herzegovina	−0.75 (−0.92 to −0.57)	<0.001	−0.53 (−0.54 to −0.51)	<0.001	−0.38 (−0.60 to −0.17)	0.001	−0.37 (−0.39 to −0.35)	<0.001	−0.98 (−1.15 to −0.81)	<0.001	−0.64 (−0.65 to −0.62)	<0.001
Botswana	2.02 (1.99−2.05)	<0.001	1.67 (1.66−1.68)	<0.001	1.89 (1.81−1.97)	<0.001	1.17 (1.17−1.18)	<0.001	2.10 (2.04−2.15)	<0.001	1.95 (1.94−1.96)	<0.001
Brazil	1.80 (1.75−1.85)	<0.001	1.10 (1.09−1.11)	<0.001	1.32 (1.29−1.35)	<0.001	0.73 (0.72−0.73)	<0.001	2.28 (2.18−2.37)	<0.001	1.39 (1.37−1.41)	<0.001
China	0.78 (0.69−0.87)	<0.001	0.07 (0.06−0.09)	<0.001	0.57 (0.39−0.75)	<0.001	−0.01 (−0.01 to 0.00)	0.003	0.95 (0.90−1.00)	<0.001	0.14 (0.11−0.16)	<0.001
Colombia	1.72 (1.70−1.74)	<0.001	0.99 (0.97−1.00)	<0.001	1.70 (1.68−1.72)	<0.001	0.93 (0.92−0.94)	<0.001	1.75 (1.71−1.80)	<0.001	1.07 (1.05−1.09)	<0.001
Costa Rica	1.82 (1.74−1.90)	<0.001	0.85 (0.84−0.86)	<0.001	2.00 (1.92−2.07)	<0.001	0.88 (0.87−0.89)	<0.001	1.56 (1.52−1.60)	<0.001	0.80 (0.80−0.81)	<0.001
Cuba	−0.10 (−0.14 to −0.07)	<0.001	−0.32 (−0.33 to −0.31)	<0.001	0.47 (0.42−0.51)	<0.001	0.27 (0.25−0.29)	<0.001	−0.66 (−0.67 to −0.65)	<0.001	−1.14 (−1.14 to −1.13)	<0.001
Dominica	0.04 (−0.02 to 0.10)	0.197	0.00 (0.00−0.01)	0.523	0.33 (0.23−0.44)	<0.001	0.05 (0.05−0.06)	<0.001	−0.23 (−0.25 to −0.21)	<0.001	−0.05 (−0.06 to −0.04)	<0.001
Dominican Republic	1.90 (1.86−1.94)	<0.001	0.75 (0.73−0.77)	<0.001	1.97 (1.91−2.03)	<0.001	0.90 (0.88−0.91)	<0.001	1.85 (1.82−1.87)	<0.001	0.65 (0.63−0.66)	<0.001

Location	Epilepsy (idiopathic and secondary)				Idiopathic epilepsy				Secondary epilepsy			
	**1990–2023**	***P*-value**	**2023–2050**	***P*-value**	**1990–2023**	***P*-value**	**2023–2050**	***P*-value**	**1990–2023**	***P*-value**	**2023–2050**	***P*-value**
Ecuador	1.98 (1.83−2.13)	<0.001	1.05 (1.04−1.05)	<0.001	2.03 (1.81−2.25)	<0.001	0.98 (0.97−0.99)	<0.001	1.90 (1.82−1.98)	<0.001	1.15 (1.15−1.16)	<0.001
El Salvador	0.93 (0.86−1.01)	<0.001	−0.09 (−0.10 to −0.08)	<0.001	1.03 (0.98−1.08)	<0.001	−0.10 (−0.11 to −0.08)	<0.001	0.83 (0.81−0.86)	<0.001	−0.08 (−0.10 to −0.07)	<0.001
Equatorial Guinea	5.41 (5.29−5.53)	<0.001	2.85 (2.82−2.88)	<0.001	5.90 (5.53−6.27)	<0.001	2.50 (2.47−2.53)	<0.001	4.93 (4.82−5.03)	<0.001	3.26 (3.25−3.28)	<0.001
Fiji	0.75 (0.72−0.78)	<0.001	0.68 (0.67−0.69)	<0.001	0.91 (0.86−0.96)	<0.001	0.67 (0.67−0.68)	<0.001	0.58 (0.55−0.61)	<0.001	0.69 (0.68−0.69)	<0.001
Gabon	2.06 (2.03−2.09)	<0.001	1.38 (1.36−1.40)	<0.001	1.86 (1.76−1.96)	<0.001	1.07 (1.06−1.08)	<0.001	2.28 (2.22−2.35)	<0.001	1.70 (1.67−1.72)	<0.001
Georgia	−1.02 (−1.08 to −0.96)	<0.001	0.23 (0.22−0.23)	<0.001	−1.06 (−1.12 to −1.00)	<0.001	0.09 (0.09−0.10)	<0.001	−0.98 (−1.02 to −0.95)	<0.001	0.35 (0.34−0.36)	<0.001
Grenada	1.20 (1.13−1.27)	<0.001	0.18 (0.17−0.19)	<0.001	1.56 (1.47−1.66)	<0.001	0.27 (0.26−0.27)	<0.001	0.89 (0.86−0.92)	<0.001	0.10 (0.10−0.10)	<0.001
Guatemala	2.73 (2.67−2.78)	<0.001	1.32 (1.31−1.33)	<0.001	2.86 (2.83−2.89)	<0.001	1.12 (1.11−1.13)	<0.001	2.62 (2.58−2.66)	<0.001	1.48 (1.46−1.50)	<0.001
Indonesia	2.17 (2.12−2.22)	<0.001	0.94 (0.92−0.95)	<0.001	1.39 (1.28−1.50)	<0.001	0.66 (0.64−0.68)	<0.001	2.99 (2.92−3.06)	<0.001	1.15 (1.13−1.18)	<0.001
Iran (Islamic Republic of)	1.57 (1.49−1.65)	<0.001	0.82 (0.80−0.83)	<0.001	0.87 (0.80−0.94)	<0.001	0.53 (0.53−0.54)	<0.001	2.59 (2.51−2.67)	<0.001	1.10 (1.08−1.12)	<0.001
Iraq	3.30 (3.05−3.55)	<0.001	1.62 (1.61−1.63)	<0.001	3.41 (3.15−3.66)	<0.001	1.82 (1.81−1.82)	<0.001	3.18 (2.98−3.37)	<0.001	1.49 (1.48−1.50)	<0.001
Jamaica	0.60 (0.56−0.64)	<0.001	−0.33 (−0.34 to −0.33)	<0.001	0.79 (0.75−0.82)	<0.001	−0.23 (−0.23 to −0.22)	<0.001	0.46 (0.42−0.50)	<0.001	−0.42 (−0.42 to −0.41)	<0.001
Kazakhstan	0.32 (0.23−0.42)	<0.001	1.18 (1.17−1.19)	<0.001	0.24 (0.17−0.32)	<0.001	1.03 (1.03−1.03)	<0.001	0.39 (0.35−0.43)	<0.001	1.34 (1.33−1.35)	<0.001
Libya	1.63 (1.60−1.67)	<0.001	0.42 (0.40−0.44)	<0.001	1.73 (1.67−1.79)	<0.001	0.47 (0.44−0.50)	<0.001	1.54 (1.49−1.59)	<0.001	0.34 (0.29−0.40)	<0.001
Malaysia	1.95 (1.88−2.02)	<0.001	1.03 (1.01−1.04)	<0.001	2.36 (2.32−2.40)	<0.001	1.33 (1.32−1.34)	<0.001	1.60 (1.56−1.64)	<0.001	0.73 (0.71−0.75)	<0.001
Maldives	2.42 (2.34−2.50)	<0.001	1.28 (1.27−1.29)	<0.001	2.82 (2.76−2.87)	<0.001	1.68 (1.67−1.69)	<0.001	2.14 (2.03−2.26)	<0.001	0.90 (0.88−0.92)	<0.001
Marshall Islands	0.79 (0.74−0.84)	<0.001	0.90 (0.90−0.91)	<0.001	1.01 (0.94−1.09)	<0.001	0.94 (0.93−0.95)	<0.001	0.63 (0.59−0.67)	<0.001	0.88 (0.87−0.88)	<0.001
Mauritius	1.36 (1.29−1.42)	<0.001	0.30 (0.28−0.32)	<0.001	1.89 (1.79−1.98)	<0.001	0.45 (0.43−0.47)	<0.001	0.73 (0.67−0.78)	<0.001	0.06 (0.05−0.07)	<0.001
Mexico	1.62 (1.59−1.64)	<0.001	1.16 (1.15−1.17)	<0.001	1.17 (1.12−1.22)	<0.001	0.96 (0.95−0.96)	<0.001	2.18 (2.15−2.22)	<0.001	1.37 (1.35−1.38)	<0.001
Mongolia	2.15 (2.10−2.21)	<0.001	2.04 (2.02−2.05)	<0.001	1.76 (1.67−1.85)	<0.001	1.32 (1.30−1.34)	<0.001	2.45 (2.42−2.48)	<0.001	2.47 (2.46−2.49)	<0.001

Location	Epilepsy (idiopathic and secondary)				Idiopathic epilepsy				Secondary epilepsy			
	**1990–2023**	***P*-value**	**2023–2050**	***P*-value**	**1990–2023**	***P*-value**	**2023–2050**	***P*-value**	**1990–2023**	***P*-value**	**2023–2050**	***P*-value**
Montenegro	−0.23 (−0.27 to −0.20)	<0.001	−0.30 (−0.30 to −0.30)	<0.001	−0.11 (−0.14 to −0.08)	<0.001	−0.15 (−0.16 to −0.15)	<0.001	−0.31 (−0.35 to −0.28)	<0.001	−0.41 (−0.41 to −0.40)	<0.001
Namibia	2.35 (2.28−2.42)	<0.001	2.17 (2.16−2.18)	<0.001	2.30 (2.15−2.45)	<0.001	1.71 (1.70−1.72)	<0.001	2.40 (2.36−2.45)	<0.001	2.45 (2.44−2.46)	<0.001
North Macedonia	0.23 (0.17−0.28)	<0.001	−0.36 (−0.37 to −0.36)	<0.001	0.31 (0.11−0.50)	0.002	−0.20 (−0.21 to −0.20)	<0.001	0.09 (0.03−0.14)	0.004	−0.48 (−0.49 to −0.47)	<0.001
Paraguay	2.17 (2.13−2.21)	<0.001	1.03 (1.02−1.04)	<0.001	2.65 (2.60−2.70)	<0.001	1.26 (1.25−1.28)	<0.001	1.81 (1.78−1.84)	<0.001	0.81 (0.80−0.82)	<0.001
Peru	1.39 (1.33−1.44)	<0.001	1.47 (1.46−1.47)	<0.001	1.16 (1.07−1.25)	<0.001	1.12 (1.11−1.13)	<0.001	1.67 (1.63−1.72)	<0.001	1.82 (1.81−1.83)	<0.001
Republic of Moldova	−1.33 (−1.45 to −1.20)	<0.001	−0.70 (−0.70 to −0.69)	<0.001	−1.57 (−1.72 to −1.41)	<0.001	−0.44 (−0.44 to −0.43)	<0.001	−1.14 (−1.21 to −1.07)	<0.001	−0.90 (−0.90 to −0.89)	<0.001
Saint Lucia	0.96 (0.94−0.99)	<0.001	0.22 (0.21−0.23)	<0.001	1.36 (1.32−1.40)	<0.001	0.07 (0.06−0.07)	<0.001	0.69 (0.68−0.71)	<0.001	0.34 (0.32−0.36)	<0.001
Saint Vincent and the Grenadines	0.41 (0.38−0.45)	<0.001	−0.25 (−0.26 to −0.24)	<0.001	0.50 (0.39−0.61)	<0.001	−0.28 (−0.29 to −0.27)	<0.001	0.33 (0.31−0.36)	<0.001	−0.23 (−0.24 to −0.21)	<0.001
Serbia	−0.45 (−0.66 to −0.23)	<0.001	−0.60 (−0.60 to −0.60)	<0.001	−0.40 (−0.64 to −0.15)	0.001	−0.58 (−0.58 to −0.57)	<0.001	−0.50 (−0.71 to −0.30)	<0.001	−0.62 (−0.63 to −0.62)	<0.001
South Africa	1.79 (1.64−1.94)	<0.001	1.16 (1.15−1.17)	<0.001	1.31 (1.17−1.44)	<0.001	0.92 (0.91−0.92)	<0.001	2.19 (2.13−2.25)	<0.001	1.32 (1.32−1.33)	<0.001
Suriname	1.57 (1.46−1.69)	<0.001	0.72 (0.70−0.74)	<0.001	1.65 (1.42−1.88)	<0.001	0.73 (0.72−0.74)	<0.001	1.50 (1.44−1.57)	<0.001	0.71 (0.70−0.73)	<0.001
Thailand	0.91 (0.84−0.99)	<0.001	0.29 (0.27−0.32)	<0.001	1.36 (1.30−1.42)	<0.001	0.56 (0.54−0.58)	<0.001	0.49 (0.45−0.54)	<0.001	−0.01 (−0.03 to 0.00)	0.031
Tonga	0.52 (0.46−0.59)	<0.001	1.68 (1.67−1.69)	<0.001	0.62 (0.57−0.67)	<0.001	1.60 (1.59−1.60)	<0.001	0.44 (0.41−0.47)	<0.001	1.75 (1.74−1.76)	<0.001
Turkey	1.23 (1.17−1.28)	<0.001	1.06 (1.05−1.07)	<0.001	1.63 (1.57−1.69)	<0.001	1.29 (1.28−1.30)	<0.001	0.78 (0.73−0.84)	<0.001	0.76 (0.75−0.78)	<0.001
Turkmenistan	1.85 (1.80−1.90)	<0.001	1.61 (1.60−1.62)	<0.001	1.87 (1.79−1.96)	<0.001	1.47 (1.46−1.48)	<0.001	1.83 (1.80−1.85)	<0.001	1.83 (1.82−1.85)	<0.001
Ukraine	−1.63 (−1.67 to −1.60)	<0.001	−1.09 (−1.10 to −1.08)	<0.001	−1.87 (−1.94 to −1.81)	<0.001	−1.15 (−1.15 to −1.15)	<0.001	−1.46 (−1.53 to −1.40)	<0.001	−1.06 (−1.06 to −1.05)	<0.001
**L-MICs**	2.12 (2.11−2.14)	<0.001	1.85 (1.85−1.86)	<0.001	2.16 (2.15−2.17)	<0.001	1.20 (1.19−1.21)	<0.001	2.10 (2.07−2.13)	<0.001	2.24 (2.22−2.26)	<0.001
Angola	4.23 (4.13−4.32)	<0.001	3.92 (3.91−3.93)	<0.001	3.99 (3.91−4.07)	<0.001	2.63 (2.61−2.64)	<0.001	4.52 (4.43−4.61)	<0.001	4.98 (4.97−5.00)	<0.001
Bangladesh	0.72 (0.66−0.78)	<0.001	0.25 (0.24−0.26)	<0.001	1.18 (1.13−1.23)	<0.001	0.01 (0.01−0.02)	<0.001	0.48 (0.41−0.56)	<0.001	0.39 (0.38−0.40)	<0.001
Benin	3.65 (3.59−3.71)	<0.001	3.31 (3.30−3.33)	<0.001	3.26 (3.22−3.30)	<0.001	2.01 (1.99−2.03)	<0.001	4.01 (3.90−4.11)	<0.001	4.13 (4.11−4.14)	<0.001

Location	Epilepsy (idiopathic and secondary)				Idiopathic epilepsy				Secondary epilepsy			
	**1990–2023**	***P*-value**	**2023–2050**	***P*-value**	**1990–2023**	***P*-value**	**2023–2050**	***P*-value**	**1990–2023**	***P*-value**	**2023–2050**	***P*-value**
Bhutan	1.65 (1.59−1.71)	<0.001	1.06 (1.06−1.07)	<0.001	1.98 (1.76−2.20)	<0.001	0.89 (0.89−0.90)	<0.001	1.45 (1.40−1.49)	<0.001	1.17 (1.16−1.17)	<0.001
Bolivia (Plurinational State of)	2.08 (1.95−2.21)	<0.001	2.15 (2.14−2.15)	<0.001	1.90 (1.80−2.00)	<0.001	1.32 (1.31−1.33)	<0.001	2.42 (2.36−2.47)	<0.001	2.95 (2.94−2.96)	<0.001
Cabo Verde	2.11 (1.97−2.24)	<0.001	1.31 (1.30−1.32)	<0.001	2.19 (2.07−2.30)	<0.001	1.07 (1.06−1.08)	<0.001	2.01 (1.87−2.15)	<0.001	1.47 (1.46−1.49)	<0.001
Cambodia	1.77 (1.74−1.80)	<0.001	1.31 (1.29−1.32)	<0.001	1.98 (1.95−2.00)	<0.001	0.95 (0.94−0.96)	<0.001	1.64 (1.60−1.68)	<0.001	1.54 (1.53−1.55)	<0.001
Cameroon	3.97 (3.90−4.04)	<0.001	2.18 (2.16−2.20)	<0.001	3.74 (3.68−3.80)	<0.001	1.31 (1.30−1.32)	<0.001	4.24 (4.16−4.33)	<0.001	2.85 (2.83−2.87)	<0.001
Comoros	2.20 (2.12−2.28)	<0.001	1.44 (1.41−1.47)	<0.001	1.55 (1.42−1.68)	<0.001	0.81 (0.80−0.82)	<0.001	2.87 (2.78−2.95)	<0.001	1.88 (1.85−1.91)	<0.001
Congo	2.67 (2.61−2.72)	<0.001	1.99 (1.98−2.00)	<0.001	2.30 (2.27−2.33)	<0.001	0.90 (0.88−0.91)	<0.001	3.06 (2.99−3.13)	<0.001	2.78 (2.77−2.78)	<0.001
Côte d'Ivoire	3.27 (3.14−3.40)	<0.001	2.65 (2.62−2.67)	<0.001	2.95 (2.92−2.98)	<0.001	1.94 (1.93−1.95)	<0.001	3.55 (3.46−3.64)	<0.001	3.13 (3.11−3.15)	<0.001
Djibouti	3.47 (3.34−3.60)	<0.001	0.95 (0.92−0.99)	<0.001	2.72 (2.56−2.88)	<0.001	0.99 (0.98−1.00)	<0.001	4.57 (4.45−4.69)	<0.001	0.92 (0.86−0.97)	<0.001
Egypt	2.21 (2.06−2.37)	<0.001	1.30 (1.29−1.31)	<0.001	2.47 (2.38−2.57)	<0.001	1.40 (1.39−1.41)	<0.001	2.07 (2.04−2.10)	<0.001	1.24 (1.23−1.25)	<0.001
Eswatini	1.85 (1.74−1.96)	<0.001	1.32 (1.31−1.33)	<0.001	1.64 (1.55−1.73)	<0.001	1.21 (1.20−1.22)	<0.001	2.01 (1.94−2.08)	<0.001	1.40 (1.39−1.42)	<0.001
Ghana	3.28 (3.24−3.31)	<0.001	2.24 (2.23−2.26)	<0.001	3.15 (3.09−3.21)	<0.001	1.67 (1.65−1.68)	<0.001	3.37 (3.32−3.42)	<0.001	2.62 (2.61−2.63)	<0.001
Guinea	3.35 (3.25−3.44)	<0.001	3.12 (3.11−3.14)	<0.001	2.92 (2.85−2.99)	<0.001	1.87 (1.85−1.88)	<0.001	3.94 (3.72−4.17)	<0.001	4.19 (4.16−4.21)	<0.001
Haiti	1.81 (1.77−1.86)	<0.001	0.90 (0.89−0.91)	<0.001	2.03 (2.00−2.07)	<0.001	0.79 (0.78−0.80)	<0.001	1.67 (1.59−1.76)	<0.001	0.96 (0.95−0.97)	<0.001
Honduras	2.36 (2.34−2.37)	<0.001	1.31 (1.30−1.32)	<0.001	2.19 (2.14−2.24)	<0.001	1.24 (1.23−1.25)	<0.001	2.50 (2.46−2.55)	<0.001	1.36 (1.36−1.37)	<0.001
India	1.78 (1.74−1.82)	<0.001	1.36 (1.35−1.37)	<0.001	1.91 (1.87−1.95)	<0.001	0.60 (0.59−0.60)	<0.001	1.71 (1.65−1.77)	<0.001	1.74 (1.73−1.74)	<0.001
Jordan	3.04 (2.92−3.16)	<0.001	1.61 (1.60−1.62)	<0.001	2.98 (2.82−3.13)	<0.001	1.29 (1.29−1.30)	<0.001	3.14 (3.04−3.24)	<0.001	1.81 (1.80−1.83)	<0.001
Kenya	3.27 (3.23−3.30)	<0.001	2.09 (2.07−2.10)	<0.001	2.70 (2.63−2.77)	<0.001	1.31 (1.30−1.32)	<0.001	3.78 (3.75−3.81)	<0.001	2.58 (2.56−2.60)	<0.001
Kiribati	1.66 (1.62−1.69)	<0.001	1.67 (1.67−1.68)	<0.001	1.42 (1.31−1.53)	<0.001	1.94 (1.94−1.94)	<0.001	1.86 (1.84−1.89)	<0.001	1.45 (1.44−1.46)	<0.001
Kyrgyzstan	0.97 (0.92−1.03)	<0.001	0.91 (0.90−0.92)	<0.001	0.25 (0.19−0.30)	<0.001	1.45 (1.43−1.46)	<0.001	1.87 (1.85−1.88)	<0.001	0.34 (0.32−0.37)	<0.001

Location	Epilepsy (idiopathic and secondary)				Idiopathic epilepsy				Secondary epilepsy			
	**1990–2023**	***P*-value**	**2023–2050**	***P*-value**	**1990–2023**	***P*-value**	**2023–2050**	***P*-value**	**1990–2023**	***P*-value**	**2023–2050**	***P*-value**
Lao People's Democratic Republic	2.41 (2.35−2.46)	<0.001	1.87 (1.87−1.88)	<0.001	2.58 (2.50−2.67)	<0.001	1.08 (1.06−1.10)	<0.001	2.30 (2.27−2.32)	<0.001	2.37 (2.37−2.38)	<0.001
Lebanon	2.19 (1.98−2.40)	<0.001	0.78 (0.77−0.79)	<0.001	2.36 (2.18−2.55)	<0.001	0.77 (0.77−0.78)	<0.001	2.01 (1.92−2.10)	<0.001	0.79 (0.77−0.82)	<0.001
Lesotho	1.06 (0.99−1.13)	<0.001	0.93 (0.93−0.93)	<0.001	1.18 (1.09−1.26)	<0.001	1.04 (1.02−1.05)	<0.001	0.99 (0.90−1.09)	<0.001	0.85 (0.85−0.85)	<0.001
Mauritania	2.83 (2.80−2.87)	<0.001	2.69 (2.68−2.70)	<0.001	2.48 (2.42−2.55)	<0.001	1.68 (1.66−1.69)	<0.001	3.07 (3.02−3.12)	<0.001	3.20 (3.18−3.22)	<0.001
Micronesia (Federated States of)	0.18 (0.14−0.23)	<0.001	1.11 (1.10−1.12)	<0.001	0.33 (0.21−0.45)	<0.001	1.09 (1.08−1.09)	<0.001	0.06 (0.04−0.08)	<0.001	1.13 (1.12−1.14)	<0.001
Morocco	0.94 (0.91−0.96)	<0.001	0.33 (0.32−0.35)	<0.001	0.92 (0.87−0.97)	<0.001	0.38 (0.38−0.39)	<0.001	0.96 (0.92−0.99)	<0.001	0.30 (0.28−0.32)	<0.001
Myanmar	1.65 (1.58−1.71)	<0.001	1.47 (1.46−1.48)	<0.001	1.70 (1.67−1.72)	<0.001	0.94 (0.92−0.96)	<0.001	1.59 (1.55−1.64)	<0.001	1.87 (1.86−1.89)	<0.001
Nepal	1.60 (1.57−1.63)	<0.001	0.87 (0.86−0.88)	<0.001	2.16 (2.10−2.22)	<0.001	0.88 (0.87−0.89)	<0.001	1.29 (1.27−1.31)	<0.001	0.86 (0.85−0.87)	<0.001
Nicaragua	1.80 (1.76−1.85)	<0.001	0.77 (0.76−0.78)	<0.001	1.78 (1.72−1.84)	<0.001	0.72 (0.72−0.73)	<0.001	1.84 (1.81−1.86)	<0.001	0.81 (0.80−0.82)	<0.001
Nigeria	4.32 (4.28−4.36)	<0.001	3.98 (3.96−4.00)	<0.001	3.63 (3.58−3.68)	<0.001	2.86 (2.84−2.87)	<0.001	5.12 (5.04−5.20)	<0.001	4.81 (4.78−4.85)	<0.001
Pakistan	2.66 (2.63−2.68)	<0.001	1.27 (1.25−1.30)	<0.001	2.24 (2.18−2.29)	<0.001	0.78 (0.77−0.79)	<0.001	3.00 (2.94−3.05)	<0.001	1.58 (1.56−1.60)	<0.001
Palestine	2.88 (2.83−2.93)	<0.001	1.39 (1.39−1.40)	<0.001	2.98 (2.91−3.06)	<0.001	1.28 (1.27−1.29)	<0.001	2.78 (2.75−2.81)	<0.001	1.50 (1.48−1.51)	<0.001
Papua New Guinea	2.86 (2.84−2.88)	<0.001	2.16 (2.15−2.18)	<0.001	2.85 (2.80−2.90)	<0.001	1.85 (1.85−1.86)	<0.001	2.87 (2.83−2.90)	<0.001	2.36 (2.35−2.36)	<0.001
Philippines	2.38 (2.36−2.41)	<0.001	1.49 (1.47−1.50)	<0.001	1.88 (1.85−1.92)	<0.001	1.21 (1.21−1.22)	<0.001	2.85 (2.78−2.91)	<0.001	1.69 (1.67−1.72)	<0.001
Samoa	0.85 (0.80−0.91)	<0.001	2.38 (2.37−2.38)	<0.001	1.01 (0.92−1.10)	<0.001	2.12 (2.12−2.12)	<0.001	0.75 (0.69−0.80)	<0.001	2.56 (2.55−2.57)	<0.001
São Tomé and Príncipe	2.67 (2.62−2.71)	<0.001	1.59 (1.57−1.62)	<0.001	2.39 (2.30−2.49)	<0.001	1.07 (1.06−1.08)	<0.001	2.89 (2.85−2.93)	<0.001	1.93 (1.91−1.95)	<0.001
Senegal	3.67 (3.63−3.70)	<0.001	3.48 (3.46−3.49)	<0.001	3.07 (2.94−3.20)	<0.001	1.90 (1.89−1.91)	<0.001	4.27 (4.15−4.40)	<0.001	4.45 (4.43−4.47)	<0.001
Solomon Islands	2.30 (2.29−2.32)	<0.001	1.38 (1.37−1.39)	<0.001	2.57 (2.53−2.61)	<0.001	1.53 (1.52−1.54)	<0.001	2.15 (2.12−2.17)	<0.001	1.28 (1.27−1.29)	<0.001
Sri Lanka	0.31 (0.24−0.38)	<0.001	0.09 (0.08−0.10)	<0.001	0.59 (0.52−0.66)	<0.001	0.41 (0.40−0.41)	<0.001	0.09 (0.06−0.11)	<0.001	−0.22 (−0.23 to −0.21)	<0.001
Tajikistan	2.16 (2.09−2.24)	<0.001	0.92 (0.89−0.95)	<0.001	1.77 (1.69−1.85)	<0.001	1.11 (1.04−1.19)	<0.001	2.71 (2.66−2.77)	<0.001	0.64 (0.60−0.69)	<0.001

Location	Epilepsy (idiopathic and secondary)				Idiopathic epilepsy				Secondary epilepsy			
	**1990–2023**	***P*-value**	**2023–2050**	***P*-value**	**1990–2023**	***P*-value**	**2023–2050**	***P*-value**	**1990–2023**	***P*-value**	**2023–2050**	***P*-value**
Timor-Leste	2.06 (2.00−2.13)	<0.001	2.01 (2.00−2.02)	<0.001	2.17 (2.06−2.27)	<0.001	1.48 (1.47−1.48)	<0.001	1.97 (1.91−2.03)	<0.001	2.29 (2.27−2.31)	<0.001
Tunisia	0.82 (0.79−0.86)	<0.001	0.53 (0.53−0.54)	<0.001	0.80 (0.73−0.86)	<0.001	0.66 (0.66−0.67)	<0.001	0.86 (0.82−0.89)	<0.001	0.39 (0.37−0.41)	<0.001
United Republic of Tanzania	3.50 (3.40−3.60)	<0.001	4.41 (4.39−4.42)	<0.001	2.75 (2.62−2.88)	<0.001	2.41 (2.39−2.42)	<0.001	4.54 (4.45−4.62)	<0.001	5.72 (5.69−5.76)	<0.001
Uzbekistan	1.87 (1.84−1.91)	<0.001	0.79 (0.78−0.80)	<0.001	1.61 (1.57−1.65)	<0.001	0.77 (0.76−0.78)	<0.001	2.27 (2.24−2.29)	<0.001	0.82 (0.81−0.83)	<0.001
Vanuatu	2.43 (2.41−2.45)	<0.001	1.87 (1.86−1.88)	<0.001	2.57 (2.55−2.59)	<0.001	1.77 (1.77−1.78)	<0.001	2.34 (2.31−2.37)	<0.001	1.93 (1.91−1.95)	<0.001
Viet Nam	1.16 (1.10−1.23)	<0.001	0.30 (0.29−0.31)	<0.001	1.59 (1.52−1.66)	<0.001	0.66 (0.64−0.67)	<0.001	0.90 (0.87−0.93)	<0.001	0.03 (0.02−0.04)	<0.001
Zambia	3.30 (3.23−3.37)	<0.001	3.51 (3.50−3.51)	<0.001	2.77 (2.68−2.85)	<0.001	2.13 (2.11−2.14)	<0.001	4.19 (4.12−4.26)	<0.001	4.79 (4.77−4.80)	<0.001
Zimbabwe	1.98 (1.90−2.06)	<0.001	1.81 (1.78−1.83)	<0.001	1.92 (1.77−2.08)	<0.001	1.79 (1.78−1.80)	<0.001	2.01 (1.93−2.09)	<0.001	1.82 (1.79−1.86)	<0.001
**LICs**	3.29 (3.25−3.32)	<0.001	3.72 (3.71−3.74)	<0.001	2.73 (2.70−2.77)	<0.001	2.12 (2.10−2.13)	<0.001	3.84 (3.74−3.94)	<0.001	4.65 (4.64−4.67)	<0.001
Afghanistan	4.26 (3.66−4.85)	<0.001	2.66 (2.64−2.68)	<0.001	4.21 (3.84−4.59)	<0.001	2.68 (2.66−2.70)	<0.001	4.27 (3.92−4.62)	<0.001	2.65 (2.63−2.67)	<0.001
Burkina Faso	3.39 (3.32−3.47)	<0.001	3.89 (3.87−3.90)	<0.001	3.35 (3.18−3.52)	<0.001	2.65 (2.64−2.66)	<0.001	3.50 (3.38−3.62)	<0.001	4.98 (4.96−5.00)	<0.001
Burundi	2.58 (2.31−2.85)	<0.001	2.73 (2.65−2.81)	<0.001	1.39 (1.03−1.75)	<0.001	2.63 (2.62−2.63)	<0.001	3.81 (3.43−4.19)	<0.001	2.80 (2.71−2.89)	<0.001
Central African Republic	1.75 (1.61−1.88)	<0.001	0.35 (0.34−0.36)	<0.001	1.58 (1.49−1.67)	<0.001	0.25 (0.24−0.26)	<0.001	1.88 (1.75−2.01)	<0.001	0.44 (0.42−0.47)	<0.001
Chad	4.28 (4.20−4.36)	<0.001	4.69 (4.68−4.70)	<0.001	4.04 (3.93−4.14)	<0.001	3.85 (3.83−3.86)	<0.001	4.54 (4.46−4.62)	<0.001	5.43 (5.41−5.44)	<0.001
Democratic People’s Republic of Korea	0.23 (0.18−0.29)	<0.001	−0.50 (−0.52 to −0.48)	<0.001	−0.06 (−0.09 to −0.03)	<0.001	−0.36 (−0.37 to −0.36)	<0.001	0.44 (0.33−0.54)	<0.001	−0.60 (−0.64 to −0.56)	<0.001
Democratic Republic of the Congo	3.29 (3.16−3.43)	<0.001	1.61 (1.46−1.76)	<0.001	2.83 (2.78−2.88)	<0.001	1.99 (1.93−2.04)	<0.001	3.76 (3.66−3.85)	<0.001	1.33 (1.09−1.57)	<0.001
Eritrea	3.46 (2.80−4.12)	<0.001	2.44 (2.42−2.45)	<0.001	2.85 (2.37−3.34)	<0.001	1.05 (1.04−1.06)	<0.001	4.07 (3.05−5.11)	<0.001	3.27 (3.25−3.30)	<0.001
Ethiopia	4.44 (4.33−4.55)	<0.001	6.03 (5.99−6.07)	<0.001	3.14 (3.04−3.24)	<0.001	2.02 (2.01−2.04)	<0.001	5.66 (5.56−5.75)	<0.001	7.38 (7.35−7.41)	<0.001
Gambia	3.59 (3.54−3.64)	<0.001	1.94 (1.91−1.97)	<0.001	3.11 (3.05−3.17)	<0.001	1.44 (1.43−1.45)	<0.001	3.94 (3.84−4.04)	<0.001	2.21 (2.17−2.24)	<0.001
Guinea-Bissau	3.04 (3.01−3.07)	<0.001	3.12 (3.12−3.13)	<0.001	2.48 (2.40−2.56)	<0.001	1.59 (1.57−1.61)	<0.001	3.62 (3.55−3.69)	<0.001	4.06 (4.05−4.07)	<0.001

Location	Epilepsy (idiopathic and secondary)				Idiopathic epilepsy				Secondary epilepsy			
	**1990–2023**	***P*-value**	**2023–2050**	***P*-value**	**1990–2023**	***P*-value**	**2023–2050**	***P*-value**	**1990–2023**	***P*-value**	**2023–2050**	***P*-value**
Liberia	2.94 (2.45−3.43)	<0.001	1.34 (1.30−1.38)	<0.001	1.78 (1.58−1.99)	<0.001	1.70 (1.68−1.72)	<0.001	3.96 (3.46−4.47)	<0.001	1.08 (1.03−1.13)	<0.001
Madagascar	3.41 (3.37−3.45)	<0.001	3.07 (3.03−3.10)	<0.001	2.41 (2.38−2.43)	<0.001	2.47 (2.45−2.48)	<0.001	4.41 (4.27−4.54)	<0.001	3.43 (3.41−3.46)	<0.001
Malawi	2.99 (2.93−3.04)	<0.001	4.07 (4.04−4.09)	<0.001	2.21 (2.12−2.30)	<0.001	1.75 (1.74−1.76)	<0.001	3.88 (3.78−3.99)	<0.001	5.39 (5.36−5.41)	<0.001
Mali	4.57 (4.46−4.68)	<0.001	5.16 (5.13−5.18)	<0.001	4.40 (4.23−4.56)	<0.001	3.18 (3.15−3.20)	<0.001	4.76 (4.69−4.83)	<0.001	6.24 (6.21−6.28)	<0.001
Mozambique	3.49 (3.38−3.60)	<0.001	2.33 (2.31−2.36)	<0.001	2.97 (2.62−3.33)	<0.001	1.40 (1.39−1.42)	<0.001	4.13 (4.04−4.22)	<0.001	3.06 (3.04−3.08)	<0.001
Niger	4.60 (4.55−4.65)	<0.001	4.67 (4.65−4.69)	<0.001	3.88 (3.85−3.92)	<0.001	3.84 (3.84−3.85)	<0.001	5.37 (5.30−5.44)	<0.001	5.27 (5.23−5.31)	<0.001
Rwanda	2.10 (1.20−3.00)	<0.001	4.73 (4.70−4.76)	<0.001	1.17 (0.32−2.03)	0.007	1.95 (1.94−1.96)	<0.001	3.12 (1.92−4.33)	<0.001	6.24 (6.20−6.27)	<0.001
Sierra Leone	3.12 (2.87−3.38)	<0.001	1.98 (1.95−2.01)	<0.001	2.74 (2.52−2.97)	<0.001	1.30 (1.29−1.32)	<0.001	3.83 (3.67−4.00)	<0.001	2.80 (2.77−2.83)	<0.001
Somalia	4.13 (4.02−4.24)	<0.001	3.81 (3.80−3.82)	<0.001	3.20 (3.04−3.36)	<0.001	2.37 (2.36−2.38)	<0.001	5.13 (5.01−5.24)	<0.001	4.69 (4.68−4.70)	<0.001
South Sudan	2.20 (2.07−2.34)	<0.001	3.99 (3.99−4.00)	<0.001	1.27 (1.19−1.35)	<0.001	2.80 (2.80−2.81)	<0.001	3.66 (3.55−3.76)	<0.001	4.97 (4.95−4.98)	<0.001
Sudan	2.85 (2.78−2.92)	<0.001	1.98 (1.96−2.00)	<0.001	2.52 (2.45−2.59)	<0.001	1.22 (1.21−1.23)	<0.001	3.13 (3.03−3.24)	<0.001	2.49 (2.46−2.51)	<0.001
Syrian Arab Republic	0.48 (0.32−0.64)	<0.001	−0.19 (−0.20 to −0.18)	<0.001	0.56 (0.37−0.74)	<0.001	0.03 (0.02−0.04)	<0.001	0.45 (0.26−0.64)	<0.001	−0.35 (−0.36 to −0.34)	<0.001
Togo	2.38 (2.30−2.45)	<0.001	1.61 (1.59−1.63)	<0.001	1.74 (1.58−1.90)	<0.001	0.98 (0.97−1.00)	<0.001	2.96 (2.87−3.05)	<0.001	2.02 (1.99−2.04)	<0.001
Uganda	4.04 (3.85−4.22)	<0.001	4.21 (4.20−4.22)	<0.001	3.49 (3.33−3.64)	<0.001	2.45 (2.43−2.46)	<0.001	4.70 (4.64−4.76)	<0.001	5.54 (5.52−5.55)	<0.001
Yemen	2.32 (2.23−2.40)	<0.001	1.33 (1.31−1.34)	<0.001	2.47 (2.32−2.62)	<0.001	1.05 (1.03−1.07)	<0.001	2.24 (2.21−2.27)	<0.001	1.46 (1.45−1.48)	<0.001

U-MICs. Upper-middle-income countries; L-MICs. Low-middle-income countries; LICs. Low-income countries

Table S4 Projected number of cases, age-standardized prevalence rate (ASPR), and all age prevalence of epilepsy (idiopathic and secondary), idiopathic epilepsy, and secondary epilepsy in 129 low- and middle-income countries (LMICs) in 2023 and 2050, along with percent changes from 2023 to 2050, both sexes (95% UI)

Location	Number of cases			ASPR			All age prevalence		
	2023 (thousand)	2050 (thousand)	Percent change (%)	2023 (per 100,000)	2050 (per 100,000)	Percent change (%)	2023 (per 100,000)	2050 (per 100,000)	Percent change (%)
**Epilepsy (idiopathic and secondary)**									
**U-MICs**									
Albania	22.01 (17.32−26.71)	21.76 (17.10−26.42)	−1.15 (−31.08 to 28.77)	798.88 (630.58−967.16)	833.68 (661.04−1006.30)	4.36 (−25.73 to 34.38)	780.54 (614.00−947.10)	796.97 (626.25−967.53)	2.11 (−28.35 to 32.48)
Algeria	239.93 (188.64−291.31)	347.94 (275.72−420.03)	45.02 (8.76−82.02)	519.52 (407.98−631.21)	581.52 (462.27−700.54)	11.93 (−19.39 to 43.22)	530.61 (417.20−644.25)	567.15 (449.43−684.67)	6.89 (−23.84 to 37.58)
Argentina	298.46 (236.81−359.95)	368.00 (291.63−444.17)	23.30 (−9.24 to 56.26)	638.65 (507.11−769.85)	689.88 (548.65−830.63)	8.02 (−22.06 to 38.12)	638.62 (506.70−770.17)	683.75 (541.86−825.26)	7.07 (−23.12 to 37.30)
Armenia	21.90 (17.50−26.30)	21.83 (17.48−26.19)	−0.33 (−28.47 to 27.79)	722.83 (578.99−866.81)	785.08 (633.32−937.18)	8.61 (−20.21 to 37.45)	720.71 (576.08−865.40)	776.02 (621.38−931.06)	7.67 (−21.64 to 36.97)
Azerbaijan	81.91 (64.33−99.48)	88.84 (69.90−107.77)	8.46 (−22.98 to 39.94)	763.98 (599.09−928.81)	815.93 (644.81−987.09)	6.80 (−24.23 to 37.82)	763.14 (599.34−926.81)	778.12 (612.23−943.92)	1.96 (−28.46 to 32.38)
Belarus	57.74 (45.93−69.58)	48.49 (38.49−58.50)	−16.02 (−42.65 to 10.84)	602.33 (481.37−723.50)	582.26 (467.48−697.12)	−3.33 (−30.92 to 24.36)	617.87 (491.44−744.52)	599.48 (475.87−723.23)	−2.98 (−31.55 to 25.56)
Belize	4.21 (3.34−5.07)	5.93 (4.70−7.16)	41.12 (5.94−77.02)	963.40 (762.77−1164.18)	1006.03 (800.10−1211.90)	4.43 (−25.33 to 34.12)	958.96 (761.05−1156.91)	1025.60 (813.15−1237.94)	6.95 (−23.23 to 37.15)
Bosnia and Herzegovina	30.64 (24.51−36.76)	26.54 (21.17−31.90)	−13.38 (−39.77 to 13.24)	916.76 (736.75−1096.68)	989.14 (796.45−1181.92)	7.90 (−20.78 to 36.56)	917.31 (733.94−1100.75)	965.48 (770.17−1160.68)	5.25 (−23.85 to 34.32)
Botswana	23.53 (18.75−28.30)	36.78 (29.55−44.03)	56.34 (19.97−93.22)	975.21 (773.76−1176.36)	1127.31 (908.37−1346.94)	15.60 (−14.78 to 46.09)	938.71 (748.21−1129.13)	1125.24 (904.08−1346.96)	19.87 (−11.00 to 50.99)
Brazil	1988.35 (1557.70−2418.91)	2669.24 (2097.72−3241.34)	34.24 (−1.10 to 70.41)	892.46 (699.56−1085.34)	1089.16 (863.64−1314.98)	22.04 (−10.95 to 55.31)	897.87 (703.40−1092.29)	1139.92 (895.84−1384.23)	26.96 (−7.42 to 61.89)
China	6363.53 (5017.27−7711.91)	6479.56 (5127.36−7834.25)	1.82 (−28.06 to 31.68)	443.58 (350.92−536.39)	508.43 (408.38−608.75)	14.62 (−16.05 to 45.37)	444.60 (350.54−538.81)	508.56 (402.43−614.88)	14.38 (−17.33 to 46.23)
Colombia	565.47 (436.92−693.86)	736.48 (567.89−904.96)	30.24 (−6.60 to 67.89)	1041.35 (805.16−1277.20)	1157.87 (898.67−1417.14)	11.19 (−22.31 to 44.81)	1049.57 (810.96−1287.88)	1198.55 (924.18−1472.73)	14.19 (−20.20 to 48.82)
Costa Rica	52.51 (40.42−64.62)	65.95 (50.53−81.38)	25.59 (−11.22 to 63.10)	1050.40 (808.88−1292.15)	1094.09 (841.38−1346.63)	4.16 (−29.03 to 37.40)	1064.23 (819.11−1309.56)	1203.21 (921.84−1484.70)	13.06 (−21.79 to 48.14)
Cuba	60.27 (47.38−73.12)	55.23 (42.71−67.78)	−8.37 (−38.11 to 21.35)	547.31 (432.69−661.47)	597.00 (469.52−724.76)	9.08 (−22.08 to 40.34)	534.35 (420.07−648.23)	585.93 (453.14−719.09)	9.65 (−22.87 to 42.46)
Dominica	0.87 (0.68−1.07)	0.88 (0.68−1.07)	0.04 (−31.73 to 31.80)	1229.25 (954.31−1503.38)	1252.97 (973.11−1533.21)	1.93 (−29.87 to 33.66)	1255.47 (973.17−1536.91)	1296.45 (1003.09−1590.18)	3.26 (−29.08 to 35.63)
Dominican Republic	108.35 (85.81−131.03)	132.34 (104.38−160.35)	22.15 (−10.76 to 55.47)	984.94 (779.28−1191.95)	1067.88 (845.45−1290.62)	8.42 (−22.29 to 39.19)	978.74 (775.16−1183.63)	1096.61 (864.90−1328.70)	12.04 (−19.36 to 43.59)

Location	Number of cases			ASPR			All age prevalence		
	2023<br>(thousand)	2050<br>(thousand)	Percent change<br>(%)	2023<br>(per 100,000)	2050<br>(per 100,000)	Percent change<br>(%)	2023<br>(per 100,000)	2050<br>(per 100,000)	Percent change<br>(%)
Ecuador	215.11<br>(163.94−266.21)	284.83<br>(217.89−351.92)	32.41<br>(−6.12 to 71.77)	1210.73<br>(922.31−1498.70)	1262.76<br>(968.42−1557.41)	4.30<br>(−29.63 to 38.13)	1196.36<br>(911.74−1480.56)	1317.26<br>(1007.66−1627.54)	10.11<br>(−24.89 to 45.23)
El Salvador	57.04<br>(44.55−69.51)	55.63<br>(43.39−67.87)	−2.46<br>(−33.04 to 28.05)	908.67<br>(709.71−1107.43)	955.24<br>(747.77−1162.39)	5.12<br>(−26.42 to 36.71)	916.41<br>(715.78−1116.77)	985.24<br>(768.46−1201.90)	7.51<br>(−24.59 to 39.71)
Equatorial Guinea	18.81<br>(14.67−22.98)	40.04<br>(31.40−48.69)	112.80<br>(62.58−164.10)	1278.97<br>(992.13−1566.75)	1590.99<br>(1246.19−1936.57)	24.40<br>(−10.40 to 59.52)	1181.89<br>(921.23−1443.59)	1553.39<br>(1218.38−1889.23)	31.43<br>(−4.01 to 67.54)
Fiji	7.04<br>(5.48−8.61)	8.44<br>(6.57−10.31)	19.90<br>(−14.39 to 54.49)	742.16<br>(576.08−908.50)	778.35<br>(606.37−950.30)	4.88<br>(−27.27 to 36.96)	741.72<br>(577.20−906.54)	774.54<br>(602.94−946.11)	4.43<br>(−27.57 to 36.45)
Gabon	24.96<br>(19.52−30.40)	36.09<br>(28.39−43.80)	44.61<br>(7.46−82.52)	1401.31<br>(1091.89−1711.17)	1506.88<br>(1186.56−1827.40)	7.53<br>(−24.18 to 39.20)	1332.65<br>(1042.36−1623.22<br>)	1503.57<br>(1182.78−1824.51)	12.83<br>(−19.49 to 45.28)
Georgia	30.95<br>(24.24−37.66)	32.85<br>(25.93−39.76)	6.15<br>(−24.90 to 37.10)	831.31<br>(652.94−1009.72)	955.97<br>(759.92−1151.47)	14.99<br>(−16.72 to 46.81)	852.43<br>(667.71−1037.19)	976.02<br>(770.33−1181.31)	14.50<br>(−17.77 to 46.89)
Grenada	1.26<br>(0.98−1.54)	1.32<br>(1.03−1.62)	4.91<br>(−26.89 to 36.73)	1106.70<br>(864.75−1348.25)	1138.08<br>(890.98−1385.37)	2.84<br>(−28.31 to 33.89)	1142.17<br>(890.87−1392.93)	1216.96<br>(948.38−1485.90)	6.55<br>(−25.55 to 38.63)
Guatemala	210.77<br>(165.07−256.47)	300.16<br>(236.20−364.23)	42.41<br>(5.78−79.84)	1138.49<br>(890.22−1386.61)	1212.07<br>(955.08−1469.51)	6.46<br>(−24.85 to 37.75)	1127.03<br>(882.63−1371.37)	1226.96<br>(965.49−1488.85)	8.87<br>(−22.81 to 40.53)
Indonesia	1305.26<br>(1043.45−1566.66)	1676.10<br>(1345.80−2005.29)	28.41<br>(−3.47 to 60.83)	479.86<br>(382.61−576.93)	591.73<br>(477.03−706.00)	23.31<br>(−7.73 to 54.55)	480.13<br>(383.82−576.28)	558.57<br>(448.50−668.28)	16.34<br>(−13.89 to 46.76)
Iran (Islamic Republic of)	513.42<br>(405.42−621.76)	637.40<br>(505.66−768.87)	24.15<br>(−8.73 to 57.37)	598.14<br>(471.20−725.55)	717.89<br>(573.10−862.54)	20.02<br>(−12.01 to 52.21)	590.86<br>(466.57−715.55)	668.91<br>(530.65−806.87)	13.21<br>(−18.12 to 44.66)
Iraq	367.01<br>(291.80−442.20)	566.63<br>(448.81−684.40)	54.39<br>(16.65−92.66)	676.08<br>(536.23−815.92)	699.30<br>(554.48−844.02)	3.43<br>(−26.26 to 33.09)	732.00<br>(581.99−881.96)	698.48<br>(553.24−843.64)	−4.58<br>(−33.04 to 23.92)
Jamaica	31.39<br>(24.68−38.10)	28.68<br>(22.46−34.92)	−8.63<br>(−37.71 to 20.54)	1116.73<br>(878.27−1355.22)	1138.74<br>(897.06−1381.45)	1.97<br>(−28.36 to 32.27)	1127.63<br>(886.43−1368.84)	1171.31<br>(917.17−1426.32)	3.87<br>(−27.09 to 34.86)
Kazakhstan	153.24<br>(120.11−186.30)	210.27<br>(166.01−254.36)	37.22<br>(1.88−73.39)	802.65<br>(628.19−976.73)	870.25<br>(688.94−1050.94)	8.42<br>(−22.81 to 39.64)	798.57<br>(625.94−970.86)	870.34<br>(687.16−1052.85)	8.99<br>(−22.35 to 40.30)
Libya	40.53<br>(31.89−49.19)	45.36<br>(35.53−55.18)	11.91<br>(−20.22 to 44.18)	534.31<br>(419.40−649.46)	532.26<br>(418.04−646.61)	−0.38<br>(−30.59 to 29.86)	545.15<br>(428.93−661.64)	505.09<br>(395.72−614.51)	−7.35<br>(−36.52 to 21.95)
Malaysia	237.54<br>(187.36−287.53)	312.75<br>(244.84−380.63)	31.66<br>(−3.19 to 67.29)	717.34<br>(564.23−869.87)	756.12<br>(594.71−917.36)	5.41<br>(−25.44 to 36.26)	715.61<br>(564.42−866.19)	746.60<br>(584.49−908.64)	4.33<br>(−26.51 to 35.21)
Maldives	3.52<br>(2.78−4.25)	4.96<br>(3.91−6.00)	40.94<br>(5.25−77.38)	695.73<br>(548.25−843.68)	758.76<br>(603.58−914.29)	9.06<br>(−21.64 to 39.79)	695.64<br>(550.94−840.72)	754.43<br>(595.32−913.79)	8.45<br>(−22.33 to 39.36)
Marshall Islands	0.37<br>(0.29−0.45)	0.47<br>(0.38−0.57)	27.49<br>(−5.64 to 61.18)	590.27<br>(465.07−715.20)	600.86<br>(476.72−725.08)	1.79<br>(−27.93 to 31.54)	606.45<br>(479.93−732.68)	603.95<br>(479.64−728.34)	−0.41<br>(−29.58 to 28.68)
Mauritius	16.88<br>(12.98−20.75)	18.25<br>(13.91−22.58)	8.13<br>(−26.20 to 42.57)	1319.64<br>(1017.14−1620.66)	1531.32<br>(1178.21−1883.47)	16.04<br>(−18.85 to 51.22)	1309.76<br>(1007.31−1610.59<br>)	1513.31<br>(1153.25−1872.33)	15.54<br>(−19.96 to 51.33)
Mexico	1861.28<br>(1444.74−2278.82)	2537.68<br>(1973.58−3102.29)	36.34<br>(−0.65 to 74.18)	1380.61<br>(1071.17−1690.79)	1445.90<br>(1128.04−1763.90)	4.73<br>(−27.33 to 36.72)	1360.98<br>(1056.40−1666.29<br>)	1516.57<br>(1179.45−1853.99)	11.43<br>(−21.82 to 44.82)

Location	Number of cases			ASPR			All age prevalence		
	2023 (thousand)	2050 (thousand)	Percent change (%)	2023 (per 100,000)	2050 (per 100,000)	Percent change (%)	2023 (per 100,000)	2050 (per 100,000)	Percent change (%)
Mongolia	28.66 (22.80−34.51)	49.25 (40.05−58.45)	71.85 (34.09−110.12)	819.54 (650.35−988.68)	1099.32 (895.31−1303.51)	34.14 (2.09−66.52)	809.19 (643.91−974.42)	1088.58 (885.34−1292.04)	34.53 (2.42−66.98)
Montenegro	4.67 (3.72−5.61)	4.31 (3.42−5.19)	−7.72 (−35.39 to 20.07)	750.17 (599.83−900.74)	744.12 (593.47−894.29)	−0.81 (−29.04 to 27.40)	744.34 (593.73−895.16)	728.57 (578.10−878.69)	−2.12 (−30.58 to 26.33)
Namibia	25.96 (20.59−31.32)	46.25 (37.10−55.40)	78.12 (37.87−119.04)	1028.89 (812.16−1244.82)	1234.43 (990.13−1478.94)	19.98 (−11.55 to 51.62)	990.52 (785.49−1194.75)	1229.99 (986.81−1473.37)	24.18 (−7.62 to 56.23)
North Macedonia	17.36 (13.93−20.80)	15.74 (12.57−18.92)	−9.32 (−36.14 to 17.59)	797.50 (641.90−952.83)	807.21 (650.89−963.27)	1.22 (−26.30 to 28.71)	792.40 (635.67−949.00)	781.81 (624.31−939.28)	−1.34 (−29.25 to 26.56)
Paraguay	68.22 (53.58−82.84)	89.90 (70.14−109.68)	31.78 (−3.58 to 67.96)	920.18 (721.41−1118.65)	957.97 (749.15−1167.15)	4.11 (−27.11 to 35.35)	907.67 (712.96−1102.24)	976.11 (761.62−1190.96)	7.54 (−24.21 to 39.43)
Peru	390.49 (302.44−478.38)	578.40 (453.41−703.32)	48.12 (9.64−87.35)	1085.01 (839.87−1329.76)	1139.42 (895.66−1383.15)	5.01 (−26.68 to 36.74)	1062.96 (823.27−1302.20)	1175.95 (921.83−1429.93)	10.63 (−22.08 to 43.31)
Republic of Moldova	25.62 (20.38−30.85)	21.22 (16.69−25.76)	−17.16 (−44.02 to 9.90)	699.12 (558.38−839.70)	711.66 (565.77−857.96)	1.79 (−27.14 to 30.73)	700.99 (557.71−844.08)	707.38 (556.31−858.79)	0.91 (−28.67 to 30.50)
Saint Lucia	2.54 (1.99−3.09)	2.69 (2.11−3.27)	6.04 (−25.44 to 37.54)	1385.36 (1085.10−1685.79)	1493.48 (1175.99−1810.52)	7.80 (−23.59 to 39.21)	1397.57 (1093.73−1701.58)	1456.34 (1140.98−1771.45)	4.21 (−27.05 to 35.44)
Saint Vincent and the Grenadines	1.28 (1.00−1.55)	1.19 (0.93−1.45)	−6.61 (−36.29 to 23.18)	1109.24 (868.99−1349.52)	1164.96 (916.43−1413.82)	5.02 (−26.07 to 36.07)	1123.21 (878.93−1367.47)	1202.33 (940.31−1464.60)	7.04 (−24.76 to 38.87)
Serbia	75.14 (59.48−90.78)	63.82 (50.39−77.27)	−15.06 (−42.31 to 12.42)	844.27 (671.73−1016.67)	843.74 (672.41−1015.20)	−0.06 (−28.73 to 28.64)	872.04 (690.39−1053.60)	882.12 (696.52−1068.00)	1.16 (−28.54 to 30.77)
South Africa	532.53 (421.41−643.63)	726.53 (578.54−874.55)	36.43 (2.33−71.33)	918.75 (725.33−1112.11)	977.00 (780.70−1173.44)	6.34 (−23.55 to 36.21)	899.82 (712.06−1087.53)	985.04 (784.40−1185.73)	9.47 (−20.97 to 39.88)
Suriname	6.90 (5.39−8.41)	8.36 (6.55−10.17)	21.25 (−12.57 to 55.37)	1152.61 (900.56−1405.15)	1235.38 (971.68−1499.00)	7.18 (−24.40 to 38.81)	1151.41 (899.73−1403.61)	1261.07 (988.46−1533.65)	9.52 (−22.59 to 41.75)
Thailand	580.19 (456.83−703.42)	626.73 (489.10−763.81)	8.02 (−23.64 to 39.80)	822.26 (650.03−994.53)	930.78 (740.56−1120.91)	13.20 (−17.85 to 44.38)	809.76 (637.60−981.76)	947.92 (739.76−1155.25)	17.06 (−15.94 to 50.41)
Tonga	0.58 (0.46−0.70)	0.91 (0.72−1.09)	56.56 (18.51−95.16)	512.69 (405.02−620.30)	522.95 (414.42−631.31)	2.00 (−27.68 to 31.68)	512.88 (406.18−619.52)	523.96 (415.73−632.03)	2.16 (−27.35 to 31.66)
Turkey	691.66 (537.44−846.16)	918.64 (711.55−1125.64)	32.82 (−3.79 to 70.30)	806.86 (626.98−987.03)	872.38 (679.61−1065.07)	8.12 (−24.47 to 40.69)	797.55 (619.72−975.71)	848.96 (657.58−1040.27)	6.45 (−26.21 to 39.14)
Turkmenistan	48.14 (37.33−58.95)	73.86 (57.69−89.94)	53.41 (13.65−93.78)	875.39 (676.99−1073.50)	1073.62 (839.68−1306.36)	22.65 (−12.07 to 57.56)	890.03 (690.15−1089.81)	1056.55 (825.30−1286.64)	18.71 (−15.37 to 52.98)
Ukraine	219.46 (175.37−263.46)	163.19 (130.22−196.17)	−25.64 (−50.55 to −0.50)	513.99 (412.50−615.27)	507.20 (408.42−605.93)	−1.32 (−28.80 to 26.14)	512.39 (409.45−615.12)	491.52 (392.23−590.86)	−4.07 (−31.92 to 23.81)
**L-MICs**									
Angola	352.76 (276.31−429.13)	993.29 (804.23−1182.66)	181.58 (124.51−239.66)	1164.91 (908.58−1420.87)	1646.13 (1325.98−1966.74)	41.31 (6.46−76.67)	1042.40 (816.49−1268.09)	1626.63 (1317.02−1936.74)	56.05 (19.99−92.96)

Location	Number of cases			ASPR			All age prevalence		
	2023 (thousand)	2050 (thousand)	Percent change (%)	2023 (per 100,000)	2050 (per 100,000)	Percent change (%)	2023 (per 100,000)	2050 (per 100,000)	Percent change (%)
Bangladesh	990.12 (784.36−1195.49)	1059.15 (849.28−1269.39)	6.97 (−22.62 to 36.48)	587.75 (465.16−710.09)	666.83 (535.45−798.35)	13.45 (−17.02 to 43.95)	601.83 (476.76−726.66)	623.22 (499.73−746.94)	3.55 (−25.55 to 32.61)
Benin	114.81 (91.32−138.25)	275.81 (226.48−324.94)	140.24 (93.43−187.98)	880.99 (697.57−1064.08)	1146.22 (937.48−1354.19)	30.11 (−1.22 to 61.58)	833.47 (662.98−1003.70)	1141.94 (937.71−1345.40)	37.01 (5.44−68.86)
Bhutan	7.98 (6.34−9.63)	10.62 (8.49−12.75)	33.00 (−0.20 to 66.85)	797.92 (631.39−964.43)	896.81 (720.08−1073.50)	12.39 (−17.92 to 42.67)	775.01 (615.16−934.97)	893.24 (714.09−1072.48)	15.26 (−15.54 to 46.17)
Bolivia (Plurinational State of)	110.88 (86.08−135.71)	196.62 (157.24−235.91)	77.32 (35.70−119.47)	895.33 (693.71−1097.13)	1051.36 (840.87−1261.32)	17.43 (−15.04 to 49.93)	857.29 (665.51−1049.27)	1050.16 (839.81−1259.95)	22.50 (−10.54 to 55.63)
Cabo Verde	6.13 (4.83−7.43)	8.69 (6.88−10.51)	41.76 (6.08−78.24)	1091.12 (858.24−1323.97)	1204.23 (957.87−1450.88)	10.37 (−20.57 to 41.30)	1056.39 (832.32−1280.39)	1281.57 (1014.53−1549.00)	21.32 (−11.39 to 54.29)
Cambodia	110.66 (88.49−132.88)	156.84 (127.53−186.11)	41.73 (9.10−75.09)	626.05 (499.08−753.34)	769.09 (626.67−911.37)	22.85 (−7.46 to 53.27)	633.99 (506.96−761.26)	760.38 (618.28−902.30)	19.94 (−9.98 to 49.95)
Cameroon	232.98 (185.22−280.98)	415.81 (338.18−493.52)	78.48 (39.61−117.79)	762.85 (603.43−922.81)	921.53 (746.92−1096.34)	20.80 (−10.08 to 51.77)	733.60 (583.23−884.74)	905.22 (736.23−1074.39)	23.39 (−7.29 to 54.20)
Comoros	7.38 (5.87−8.90)	10.81 (8.75−12.86)	46.40 (12.52−81.05)	908.08 (719.56−1097.12)	1127.51 (913.09−1341.12)	24.16 (−7.10 to 55.55)	943.57 (750.49−1137.29)	1110.03 (898.84−1320.37)	17.64 (−12.53 to 47.93)
Congo	58.12 (45.84−70.42)	98.53 (79.64−117.44)	69.51 (31.19−108.42)	1142.64 (897.26−1388.65)	1493.17 (1207.20−1779.51)	30.68 (−2.07 to 63.70)	1081.44 (852.99−1310.22)	1486.47 (1201.49−1771.79)	37.45 (3.99−71.39)
Côte d'Ivoire	264.62 (210.44−318.90)	533.76 (431.07−636.41)	101.71 (58.40−145.76)	950.05 (751.67−1148.96)	1112.45 (895.94−1328.93)	17.09 (−13.68 to 47.99)	908.24 (722.29−1094.55)	1103.71 (891.37−1315.98)	21.52 (−9.38 to 52.60)
Djibouti	11.02 (8.71−13.33)	14.21 (11.24−17.18)	29.00 (−4.64 to 63.28)	856.10 (671.75−1040.50)	929.48 (736.07−1122.84)	8.57 (−22.51 to 39.72)	877.11 (693.25−1061.02)	910.00 (719.71−1100.30)	3.75 (−26.34 to 33.79)
Egypt	726.08 (577.74−874.57)	1028.70 (821.85−1235.24)	41.68 (7.31−76.82)	630.68 (500.68−760.79)	689.13 (550.56−827.50)	9.27 (−20.74 to 39.25)	677.72 (539.27−816.33)	685.34 (547.54−822.94)	1.12 (−27.56 to 29.85)
Eswatini	10.64 (8.42−12.86)	15.16 (11.98−18.33)	42.39 (6.61−78.91)	919.50 (722.54−1116.24)	956.06 (754.55−1157.28)	3.98 (−26.55 to 34.42)	859.39 (679.82−1038.66)	929.16 (734.43−1123.52)	8.12 (−22.54 to 38.90)
Ghana	284.97 (227.38−342.64)	516.50 (418.45−614.35)	81.25 (41.90−121.20)	871.68 (691.81−1051.65)	1068.64 (864.14−1272.75)	22.59 (−8.47 to 53.80)	837.24 (668.05−1006.69)	1059.33 (858.24−1260.03)	26.53 (−4.57 to 57.86)
Guinea	90.78 (72.00−109.57)	207.55 (170.60−244.53)	128.64 (83.62−174.46)	620.19 (489.25−751.27)	871.43 (713.25−1029.79)	40.51 (7.69−73.66)	658.90 (522.60−795.32)	906.95 (745.49−1068.54)	37.65 (5.85−69.67)
Haiti	99.09 (78.70−119.45)	126.05 (100.76−151.40)	27.20 (−5.29 to 60.13)	773.30 (612.16−934.36)	784.07 (626.86−941.71)	1.39 (−27.67 to 30.42)	766.13 (608.50−923.50)	785.68 (628.09−943.73)	2.55 (−26.43 to 31.50)
Honduras	123.67 (97.05−150.32)	175.61 (138.14−213.06)	42.00 (5.48−79.27)	1194.52 (935.41−1453.92)	1183.67 (931.56−1435.69)	−0.91 (−31.12 to 29.29)	1163.35 (912.94−1414.09)	1193.16 (938.60−1447.61)	2.56 (−28.03 to 33.13)
India	9977.76 (7901.80−12055.19)	14333.23 (11450.65−17227.23)	43.65 (8.69−79.41)	690.08 (545.61−834.68)	925.08 (739.65−1111.37)	34.06 (0.46−68.30)	683.58 (541.35−825.90)	893.08 (713.47−1073.40)	30.65 (−2.49 to 64.37)
Jordan	72.71 (57.67−87.77)	111.65 (88.78−134.46)	53.56 (16.44−91.21)	577.22 (457.02−697.60)	641.20 (510.55−771.45)	11.09 (−19.52 to 41.81)	606.20 (480.82−731.79)	624.95 (496.92−752.61)	3.09 (−26.36 to 32.49)

Location	Number of cases			ASPR			All age prevalence		
	2023 (thousand)	2050 (thousand)	Percent change (%)	2023 (per 100,000)	2050 (per 100,000)	Percent change (%)	2023 (per 100,000)	2050 (per 100,000)	Percent change (%)
Kenya	505.85 (405.07−607.12)	881.32 (713.34−1049.09)	74.22 (35.88−112.96)	922.60 (734.00−1111.77)	1147.08 (926.25−1367.72)	24.33 (−6.91 to 55.83)	926.95 (742.27−1112.52)	1133.86 (917.75−1349.72)	22.32 (−8.12 to 53.03)
Kiribati	0.86 (0.68−1.04)	1.35 (1.05−1.64)	56.54 (16.97−96.56)	628.24 (494.57−761.63)	625.40 (488.19−762.69)	−0.45 (−30.84 to 29.86)	639.62 (506.15−772.77)	636.04 (497.81−774.39)	−0.56 (−30.52 to 29.38)
Kyrgyzstan	50.96 (40.54−61.38)	65.07 (51.05−79.03)	27.69 (−5.88 to 62.06)	714.10 (566.55−861.68)	697.13 (547.01−846.61)	−2.38 (−31.72 to 26.94)	731.35 (581.79−880.92)	692.68 (543.40−841.31)	−5.29 (−34.01 to 23.45)
Lao People's Democratic Republic	47.29 (37.69−56.90)	77.88 (63.87−91.87)	64.70 (29.38−100.62)	616.26 (489.29−743.50)	865.60 (710.97−1020.15)	40.46 (8.27−72.97)	622.61 (496.30−749.25)	848.68 (696.06−1001.20)	36.31 (4.78−68.18)
Lebanon	60.55 (47.21−73.87)	74.67 (58.03−91.30)	23.31 (−11.55 to 58.66)	645.54 (502.37−788.61)	679.39 (529.12−829.66)	5.24 (−26.79 to 37.32)	658.66 (513.50−803.54)	655.02 (509.10−800.97)	−0.55 (−31.66 to 30.54)
Lesotho	15.50 (12.24−18.74)	19.89 (15.60−24.19)	28.34 (−5.77 to 63.23)	781.54 (614.40−948.27)	817.49 (640.62−994.88)	4.60 (−26.42 to 35.59)	746.75 (590.00−902.97)	796.11 (624.51−968.12)	6.61 (−24.36 to 37.68)
Mauritania	44.16 (35.33−52.98)	90.26 (73.74−106.79)	104.42 (62.54−147.07)	906.60 (722.73−1090.52)	1185.91 (967.40−1404.49)	30.81 (−0.41 to 62.27)	968.77 (775.16−1162.45)	1205.94 (985.22−1426.73)	24.48 (−5.66 to 54.79)
Micronesia (Federated States of)	0.72 (0.57−0.87)	0.97 (0.77−1.17)	34.71 (0.11−70.09)	624.24 (490.17−758.67)	645.07 (510.31−780.13)	3.34 (−27.03 to 33.68)	637.05 (501.85−772.57)	646.08 (511.51−780.96)	1.42 (−28.42 to 31.27)
Morocco	222.95 (176.94−269.04)	243.84 (194.27−293.39)	9.37 (−20.87 to 39.64)	587.68 (465.98−709.59)	617.60 (493.68−741.53)	5.09 (−24.37 to 34.52)	593.21 (470.79−715.83)	574.53 (457.75−691.28)	−3.15 (−31.59 to 25.37)
Myanmar	323.62 (256.34−390.64)	478.92 (387.77−569.98)	47.99 (13.68−83.07)	582.95 (460.93−704.52)	778.18 (632.28−923.93)	33.49 (1.21−66.06)	580.69 (459.97−700.95)	754.18 (610.65−897.58)	29.88 (−2.09 to 62.11)
Nepal	324.54 (255.87−393.26)	409.44 (325.32−493.49)	26.16 (−7.01 to 59.67)	1047.33 (823.39−1271.28)	1167.84 (930.85−1404.78)	11.51 (−19.49 to 42.50)	1020.54 (804.61−1236.63)	1194.10 (948.78−1439.25)	17.01 (−14.85 to 49.00)
Nicaragua	63.52 (49.78−77.22)	78.13 (61.19−95.04)	23.01 (−11.00 to 57.41)	932.34 (730.17−1133.95)	953.07 (748.73−1157.02)	2.22 (−28.46 to 32.89)	921.18 (721.99−1119.83)	986.28 (772.43−1199.73)	7.07 (−24.52 to 38.69)
Nigeria	1899.19 (1519.88−2279.22)	5428.80 (4424.38−6434.00)	185.85 (130.19−242.63)	762.05 (606.20−918.13)	1074.57 (872.59−1276.74)	41.01 (8.04−74.64)	766.92 (613.75−920.38)	1107.60 (902.68−1312.68)	44.42 (11.67−77.94)
Pakistan	2091.48 (1650.02−2533.34)	2935.85 (2344.12−3527.50)	40.37 (5.75−75.81)	826.68 (649.46−1003.90)	970.79 (774.36−1167.21)	17.43 (−14.43 to 49.42)	880.07 (694.31−1066.00)	956.36 (763.61−1149.10)	8.67 (−21.68 to 38.99)
Palestine	35.84 (28.30−43.37)	52.06 (41.17−62.94)	45.24 (8.95−82.21)	606.48 (478.16−734.59)	622.26 (491.97−752.57)	2.60 (−27.43 to 32.61)	650.40 (513.56−787.09)	622.51 (492.32−752.71)	−4.29 (−33.22 to 24.72)
Papua New Guinea	54.09 (43.04−65.19)	96.34 (77.33−115.33)	78.12 (38.03−118.83)	508.25 (402.50−614.40)	542.64 (434.23−650.86)	6.77 (−22.95 to 36.40)	509.47 (405.44−614.00)	552.39 (443.38−661.24)	8.42 (−21.10 to 38.00)
Philippines	793.43 (627.27−959.34)	1179.28 (937.48−1421.40)	48.63 (12.25−85.62)	684.42 (539.50−829.14)	772.45 (613.98−931.15)	12.86 (−18.37 to 44.19)	695.80 (550.08−841.29)	772.78 (614.33−931.44)	11.06 (−19.73 to 41.98)
Samoa	1.27 (1.01−1.53)	2.40 (1.92−2.88)	88.39 (45.93−131.53)	582.44 (460.23−704.65)	639.22 (509.32−768.85)	9.75 (−20.73 to 40.20)	570.61 (452.66−688.55)	644.50 (515.29−773.45)	12.95 (−17.55 to 43.54)
Sao Tome and Principe	2.30 (1.83−2.77)	3.51 (2.82−4.21)	52.85 (16.74−89.50)	1094.85 (866.93−1322.40)	1289.33 (1032.84−1545.97)	17.76 (−13.36 to 49.02)	1056.19 (839.47−1272.56)	1283.83 (1028.91−1538.93)	21.55 (−9.88 to 53.17)

Location	Number of cases			ASPR			All age prevalence		
	2023 (thousand)	2050 (thousand)	Percent change (%)	2023 (per 100,000)	2050 (per 100,000)	Percent change (%)	2023 (per 100,000)	2050 (per 100,000)	Percent change (%)
Senegal	149.88 (119.58−180.17)	375.54 (310.54−440.50)	150.57 (103.38−198.70)	822.39 (652.89−991.78)	1292.73 (1066.86−1518.46)	57.19 (23.51−91.66)	875.13 (698.24−1052.02)	1339.17 (1107.37−1570.83)	53.03 (20.27−86.48)
Solomon Islands	4.08 (3.27−4.89)	5.91 (4.73−7.08)	44.73 (10.41−79.70)	551.13 (438.77−662.82)	551.28 (440.79−661.62)	0.03 (−28.44 to 28.43)	567.66 (454.32−680.25)	565.89 (453.64−678.03)	−0.31 (−28.23 to 27.64)
Sri Lanka	219.57 (171.65−267.48)	225.01 (174.77−275.07)	2.48 (−29.00 to 34.02)	994.31 (778.43−1210.16)	1026.93 (806.81−1246.55)	3.28 (−27.61 to 34.11)	994.58 (777.52−1211.60)	1074.53 (834.59−1313.55)	8.04 (−24.28 to 40.50)
Tajikistan	95.31 (74.80−115.70)	121.96 (94.79−149.16)	27.96 (−7.07 to 63.81)	875.85 (684.78−1065.74)	751.54 (583.50−919.84)	−14.19 (−43.03 to 14.85)	908.88 (713.30−1103.29)	759.38 (590.25−928.75)	−16.45 (−44.67 to 11.99)
Timor-Leste	8.90 (7.17−10.64)	15.22 (12.48−17.95)	70.96 (34.82−107.55)	588.67 (472.22−705.07)	684.68 (560.68−808.71)	16.31 (−12.49 to 45.07)	616.87 (496.50−737.20)	688.56 (564.86−812.33)	11.62 (−16.27 to 39.45)
Tunisia	67.03 (52.55−81.46)	77.38 (60.51−94.31)	15.45 (−17.47 to 48.64)	560.93 (439.67−681.82)	585.34 (461.41−709.58)	4.35 (−26.46 to 35.09)	554.69 (434.85−674.16)	569.42 (445.28−693.98)	2.66 (−28.37 to 33.69)
United Republic of Tanzania	596.44 (472.86−720.47)	1908.00 (1579.29−2236.53)	219.90 (161.96−279.02)	896.66 (706.44−1087.03)	1510.03 (1246.86−1773.06)	68.41 (32.90−104.74)	921.24 (730.36−1112.81)	1558.86 (1290.30−1827.27)	69.21 (34.08−105.08)
Uzbekistan	266.56 (209.36−323.60)	329.62 (259.50−399.57)	23.66 (−9.96 to 57.62)	745.56 (583.59−907.03)	780.79 (615.44−945.76)	4.73 (−26.18 to 35.55)	763.46 (599.64−926.83)	770.42 (606.54−933.91)	0.91 (−29.27 to 31.06)
Vanuatu	1.86 (1.48−2.24)	3.07 (2.45−3.69)	64.67 (26.08−103.72)	557.22 (441.53−672.84)	580.15 (462.24−698.10)	4.11 (−25.43 to 33.60)	570.63 (454.29−687.02)	595.02 (475.36−714.72)	4.27 (−24.88 to 33.37)
Viet Nam	500.01 (399.51−600.40)	541.66 (429.16−653.97)	8.33 (−21.65 to 38.42)	502.91 (401.38−604.32)	523.46 (417.31−629.55)	4.09 (−25.02 to 33.24)	495.45 (395.87−594.93)	508.64 (403.00−614.11)	2.66 (−26.50 to 31.80)
Zambia	222.86 (174.18−271.68)	564.12 (458.35−669.80)	153.13 (101.65−205.67)	1078.08 (837.38−1319.35)	1544.16 (1249.67−1838.34)	43.23 (8.26−78.57)	1079.18 (843.47−1315.61)	1569.82 (1275.49−1863.93)	45.46 (10.85−80.57)
Zimbabwe	154.89 (122.69−187.05)	250.90 (198.60−303.02)	61.99 (22.69−101.74)	954.91 (751.77−1157.88)	953.13 (751.95−1153.56)	−0.19 (−29.99 to 29.56)	913.52 (723.63−1103.20)	924.99 (732.18−1117.14)	1.26 (−28.23 to 30.73)
**LICs**									
Afghanistan	256.45 (206.40−306.32)	520.66 (418.98−622.00)	103.02 (59.49−147.42)	547.85 (439.79−655.34)	553.86 (445.02−662.50)	1.10 (−26.74 to 28.93)	641.24 (516.09−765.93)	621.01 (499.74−741.89)	−3.15 (−30.23 to 23.94)
Burkina Faso	158.38 (125.73−191.04)	443.10 (364.27−521.79)	179.76 (126.84−233.80)	628.97 (495.53−762.16)	872.67 (713.31−1031.73)	38.75 (6.04−71.73)	625.79 (496.78−754.82)	891.87 (733.20−1050.25)	42.52 (10.14−75.24)
Burundi	100.57 (80.97−120.16)	207.24 (166.91−247.52)	106.07 (62.15−150.89)	775.64 (619.76−931.27)	789.97 (632.49−947.55)	1.85 (−26.60 to 30.29)	768.90 (619.05−918.65)	785.15 (632.35−937.75)	2.11 (−25.62 to 29.79)
Central African Republic	37.14 (29.38−44.89)	40.83 (32.09−49.55)	9.96 (−21.27 to 41.33)	829.72 (652.66−1006.66)	826.67 (648.91−1004.01)	−0.37 (−30.46 to 29.69)	768.93 (608.22−929.47)	808.01 (635.03−980.44)	5.08 (−25.48 to 35.69)
Chad	111.14 (88.29−133.98)	382.30 (311.68−452.94)	243.99 (177.88−311.31)	574.50 (453.40−695.65)	743.73 (602.17−885.03)	29.46 (−2.70 to 61.89)	581.81 (462.20−701.38)	776.59 (633.12−920.08)	33.48 (1.68−65.58)
Democratic People's Republic of Korea	119.43 (96.03−142.84)	104.11 (83.34−124.89)	−12.83 (−38.85 to 13.39)	466.91 (375.63−558.18)	470.94 (379.68−562.21)	0.86 (−26.66 to 28.35)	459.79 (369.70−549.91)	442.32 (354.06−530.60)	−3.80 (−31.17 to 23.52)

Location	Number of cases			ASPR			All age prevalence		
	**2023 (thousand)**	**2050 (thousand)**	**Percent change (%)**	**2023 (per 100,000)**	**2050 (per 100,000)**	**Percent change (%)**	**2023 (per 100,000)**	**2050 (per 100,000)**	**Percent change (%)**
Democratic Republic of the Congo	771.43 (616.89−925.30)	1214.10 (955.35−1473.45)	57.38 (18.64−96.47)	848.66 (674.89−1021.64)	763.26 (599.49−927.34)	−10.06 (−38.06 to 18.04)	805.47 (644.12−966.13)	718.49 (565.37−871.97)	−10.80 (−38.30 to 16.78)
Eritrea	58.79 (46.87−70.70)	112.22 (92.38−132.02)	90.86 (51.91−130.18)	879.97 (696.40−1063.19)	1280.26 (1050.38−1509.86)	45.49 (12.43−79.04)	896.75 (714.91−1078.32)	1276.71 (1051.09−1502.08)	42.37 (10.37−74.79)
Ethiopia	913.11 (735.56−1090.97)	4423.76 (3720.04−5126.59)	384.47 (305.51−464.49)	738.40 (590.61−886.30)	2104.02 (1766.61−2440.89)	184.94 (135.78−235.04)	745.29 (600.37−890.46)	2155.14 (1812.30−2497.54)	189.17 (139.98−239.25)
Gambia	21.29 (17.13−25.43)	35.67 (29.09−42.27)	67.55 (31.27−104.32)	799.18 (640.59−956.90)	944.18 (767.91−1120.63)	18.14 (−11.36 to 47.74)	870.04 (699.80−1038.99)	970.66 (791.36−1150.18)	11.57 (−16.68 to 39.82)
Guinea-Bissau	17.07 (13.63−20.49)	38.93 (32.28−45.59)	128.11 (84.90−172.08)	763.59 (605.24−921.42)	1128.27 (932.03−1324.55)	47.76 (15.06−80.89)	790.44 (631.17−948.88)	1166.09 (966.91−1365.39)	47.52 (15.63−79.91)
Liberia	46.70 (37.48−55.90)	66.93 (52.61−81.27)	43.34 (7.22−79.99)	893.09 (712.97−1073.02)	855.62 (672.09−1039.41)	−4.20 (−32.90 to 24.45)	859.35 (689.81−1028.68)	829.72 (652.16−1007.51)	−3.45 (−31.90 to 25.07)
Madagascar	258.99 (207.55−310.29)	583.40 (473.38−693.49)	125.26 (79.00−172.44)	807.71 (643.40−971.57)	915.84 (740.00−1091.69)	13.39 (−16.31 to 43.07)	825.71 (661.71−989.25)	943.29 (765.40−1121.29)	14.24 (−14.91 to 43.51)
Malawi	182.50 (145.49−219.60)	534.51 (448.24−620.74)	192.89 (142.25−244.57)	884.66 (700.03−1069.36)	1591.56 (1329.60−1853.49)	79.91 (44.28−116.24)	901.32 (718.58−1084.56)	1618.64 (1357.39−1879.76)	79.59 (44.81−115.09)
Mali	139.67 (112.53−166.82)	542.36 (456.47−628.17)	288.32 (224.48−353.24)	517.80 (414.89−620.66)	957.64 (803.36−1111.72)	84.95 (49.65−120.78)	567.04 (456.88−677.27)	1034.26 (870.48−1197.90)	82.40 (48.07−117.22)
Mozambique	325.33 (257.39−393.12)	604.37 (490.98−717.62)	85.77 (45.51−126.34)	966.72 (757.70−1175.47)	1215.09 (981.24−1448.45)	25.69 (−6.55 to 58.05)	943.53 (746.49−1140.14)	1192.48 (968.74−1415.94)	26.38 (−5.02 to 57.93)
Niger	158.60 (127.46−189.57)	542.83 (445.74−640.09)	242.27 (178.59−307.07)	534.96 (427.40−642.10)	659.82 (539.15−780.60)	23.34 (−6.65 to 53.47)	583.39 (468.84−697.32)	720.95 (592.00−850.13)	23.58 (−5.78 to 53.05)
Rwanda	120.29 (95.93−144.60)	418.32 (353.61−483.06)	247.77 (191.20−305.49)	824.39 (653.58−994.95)	1624.14 (1370.45−1877.89)	97.01 (60.43−134.13)	818.56 (652.81−984.04)	1644.20 (1389.85−1898.66)	100.86 (64.23−138.05)
Sierra Leone	65.25 (51.16−79.39)	110.53 (88.87−132.11)	69.40 (30.35−109.04)	690.81 (539.02−843.21)	835.90 (669.83−1001.38)	21.00 (−11.41 to 53.53)	724.68 (568.24−881.74)	836.28 (672.41−999.55)	15.40 (−15.79 to 46.64)
Somalia	131.20 (105.52−156.93)	359.32 (299.34−419.18)	173.87 (124.95−223.78)	596.51 (475.83−717.44)	856.05 (708.80−1002.99)	43.51 (11.88−75.46)	652.24 (524.59−780.13)	932.52 (776.86−1087.87)	42.97 (12.39−73.87)
South Sudan	74.62 (59.29−89.97)	214.43 (176.29−252.41)	187.36 (133.15−242.56)	588.42 (463.94−712.97)	737.70 (602.67−872.22)	25.37 (−5.69 to 56.54)	614.57 (488.35−740.98)	776.69 (638.52−914.23)	26.38 (−3.90 to 56.76)
Sudan	256.39 (204.28−308.62)	434.07 (354.67−513.23)	69.30 (32.79−106.36)	492.34 (392.10−592.78)	612.49 (499.93−724.70)	24.40 (−6.02 to 54.94)	551.42 (439.35−663.76)	616.25 (503.52−728.63)	11.76 (−16.94 to 40.43)
Syrian Arab Republic	97.61 (77.60−117.66)	92.70 (73.46−111.96)	−5.03 (−33.42 to 23.40)	510.10 (405.14−615.09)	497.89 (395.74−600.22)	−2.39 (−31.06 to 26.25)	531.85 (422.78−641.05)	464.54 (368.10−561.04)	−12.66 (−39.88 to 14.77)
Togo	75.84 (60.43−91.26)	116.44 (94.04−138.91)	53.54 (18.23−89.42)	936.41 (742.95−1129.91)	1005.13 (810.56−1200.27)	7.34 (−21.87 to 36.54)	891.99 (710.70−1073.31)	992.01 (801.13−1183.42)	11.21 (−18.20 to 40.65)
Uganda	493.03 (390.54−595.26)	1499.14 (1239.75−1758.27)	204.06 (148.24−260.83)	1027.57 (807.88−1246.70)	1665.05 (1371.27−1958.53)	62.04 (27.05−97.84)	1037.16 (821.57−1252.20)	1692.24 (1399.44−1984.74)	63.16 (28.80−98.28)

Location	Number of cases			ASPR			All age prevalence		
	2023 (thousand)	2050 (thousand)	Percent change (%)	2023 (per 100,000)	2050 (per 100,000)	Percent change (%)	2023 (per 100,000)	2050 (per 100,000)	Percent change (%)
Yemen	193.96 (155.86−232.01)	276.63 (225.10−328.17)	42.62 (10.21−75.78)	482.10 (386.77−577.33)	521.18 (423.61−618.78)	8.11 (−20.09 to 36.23)	547.85 (440.23−655.31)	527.86 (429.53−626.20)	−3.65 (−30.06 to 23.03)
**Idiopathic epilepsy**									
**U-MICs**									
Albania	12.37 (8.91−15.84)	12.58 (9.06−16.10)	1.68 (−38.08 to 41.41)	435.54 (313.60−557.35)	448.44 (323.39−573.43)	2.96 (−37.00 to 42.84)	438.71 (315.88−561.46)	460.79 (331.80−589.59)	5.03 (−35.39 to 45.52)
Algeria	125.57 (90.71−160.51)	168.27 (120.95−215.49)	34.00 (−11.91 to 80.95)	275.55 (199.01−352.19)	270.43 (194.32−346.34)	−1.86 (−40.85 to 37.15)	277.71 (200.62−354.98)	274.29 (197.15−351.26)	−1.23 (−40.33 to 37.82)
Argentina	124.43 (89.52−159.14)	155.62 (112.12−198.98)	25.06 (−19.23 to 69.99)	265.40 (190.94−339.46)	285.06 (205.31−364.45)	7.41 (−33.49 to 48.29)	266.24 (191.53−340.51)	289.14 (208.32−369.71)	8.60 (−32.48 to 49.83)
Armenia	8.87 (6.38−11.35)	8.67 (6.25−11.09)	−2.27 (−41.30 to 36.76)	291.40 (209.85−373.20)	297.04 (214.35−379.98)	1.94 (−37.83 to 41.69)	291.78 (210.08−373.68)	308.06 (222.23−394.23)	5.58 (−34.95 to 46.15)
Azerbaijan	44.46 (32.01−56.90)	49.06 (35.33−62.77)	10.34 (−31.10 to 51.96)	421.02 (303.11−538.84)	429.32 (309.13−549.55)	1.97 (−37.89 to 41.74)	414.26 (298.21−530.15)	429.70 (309.48−549.81)	3.73 (−36.48 to 43.90)
Belarus	25.04 (18.02−32.06)	21.71 (15.65−27.78)	−13.31 (−50.09 to 23.79)	255.44 (183.70−327.29)	241.23 (173.69−308.82)	−5.56 (−43.98 to 33.03)	267.96 (192.86−343.09)	268.39 (193.42−343.48)	0.16 (−39.28 to 39.56)
Belize	1.72 (1.24−2.19)	2.39 (1.72−3.06)	39.14 (−8.02 to 87.27)	394.95 (284.82−505.04)	398.58 (286.60−510.72)	0.92 (−38.72 to 40.50)	391.42 (282.43−500.22)	412.76 (296.78−528.85)	5.45 (−35.07 to 45.92)
Bosnia and Herzegovina	12.70 (9.14−16.25)	11.47 (8.25−14.68)	−9.70 (−47.14 to 28.15)	374.39 (269.65−478.91)	405.98 (292.58−519.62)	8.44 (−32.61 to 49.65)	380.17 (273.73−486.50)	417.15 (300.23−534.21)	9.72 (−31.63 to 51.27)
Botswana	8.88 (6.40−11.36)	12.17 (8.77−15.59)	37.03 (−9.45 to 84.63)	373.41 (269.32−477.39)	366.58 (264.23−469.53)	−1.83 (−40.86 to 37.11)	354.35 (255.48−453.15)	372.31 (268.40−476.84)	5.07 (−35.29 to 45.46)
Brazil	902.81 (650.78−1154.76)	1097.66 (790.49−1405.71)	21.58 (−22.07 to 65.68)	405.62 (292.39−518.85)	410.97 (296.26−526.16)	1.32 (−38.32 to 40.93)	407.68 (293.87−521.45)	468.76 (337.58−600.32)	14.98 (−27.39 to 57.59)
China	2838.54 (2041.86−3635.24)	2831.70 (2036.78−3627.49)	−0.24 (−39.72 to 39.19)	197.68 (142.15−253.21)	198.70 (142.77−254.76)	0.52 (−39.22 to 40.25)	198.32 (142.66−253.98)	222.25 (159.86−284.71)	12.07 (−29.84 to 54.13)
Colombia	326.22 (235.42−416.82)	418.39 (300.65−536.04)	28.26 (−16.58 to 73.99)	598.51 (431.93−764.67)	626.71 (450.38−803.04)	4.71 (−35.61 to 45.02)	605.49 (436.96−773.67)	680.89 (489.27−872.34)	12.45 (−29.43 to 54.54)
Costa Rica	29.78 (21.44−38.14)	37.74 (27.11−48.38)	26.74 (−18.06 to 72.36)	597.21 (430.04−764.74)	626.86 (450.06−803.68)	4.97 (−35.62 to 45.56)	603.53 (434.44−772.99)	688.58 (494.63−882.66)	14.09 (−28.28 to 56.77)
Cuba	32.57 (23.47−41.62)	34.89 (25.08−44.75)	7.15 (−33.77 to 48.22)	278.64 (200.98−355.83)	320.05 (229.70−410.81)	14.86 (−27.55 to 57.62)	288.72 (208.04−368.96)	370.19 (266.10−474.75)	28.22 (−16.70 to 74.04)
Dominica	0.44 (0.32−0.56)	0.45 (0.32−0.57)	1.39 (−38.38 to 41.10)	613.05 (441.07−784.28)	634.52 (456.71−812.63)	3.50 (−36.72 to 43.70)	630.32 (453.63−806.21)	659.68 (474.65−845.10)	4.66 (−35.77 to 45.12)
Dominican Republic	42.35 (30.59−54.23)	53.84 (38.72−68.99)	27.12 (−17.54 to 72.66)	386.52 (279.12−494.93)	428.20 (308.05−548.46)	10.78 (−30.81 to 52.52)	382.60 (276.32−489.86)	446.14 (320.84−571.68)	16.61 (−26.13 to 59.61)

Location	Number of cases			ASPR			All age prevalence		
	2023 (thousand)	2050 (thousand)	Percent change (%)	2023 (per 100,000)	2050 (per 100,000)	Percent change (%)	2023 (per 100,000)	2050 (per 100,000)	Percent change (%)
Ecuador	135.00 (97.34−172.60)	175.69 (126.45−225.02)	30.14 (−15.03 to 76.27)	755.29 (544.49−965.72)	778.56 (560.25−996.99)	3.08 (−36.99 to 43.11)	750.81 (541.35−959.92)	812.51 (584.80−1040.63)	8.22 (−32.80 to 49.40)
El Salvador	27.28 (19.64−34.90)	26.56 (19.10−34.00)	−2.65 (−41.67 to 36.32)	433.09 (311.82−554.12)	458.06 (329.37−586.50)	5.77 (−34.86 to 46.35)	438.31 (315.49−560.80)	470.31 (338.33−602.18)	7.30 (−33.65 to 48.31)
Equatorial Guinea	10.75 (7.76−13.76)	20.91 (15.08−26.77)	94.48 (34.32−155.76)	704.00 (508.19−900.57)	829.96 (598.26−1062.21)	17.89 (−24.92 to 60.96)	675.49 (487.73−864.11)	811.40 (584.89−1038.50)	20.12 (−23.10 to 63.81)
Fiji	3.77 (2.71−4.83)	4.51 (3.25−5.78)	19.77 (−23.49 to 63.39)	405.89 (292.28−519.91)	413.18 (297.71−528.66)	1.80 (−38.00 to 41.58)	396.98 (285.91−508.52)	414.10 (298.36−529.82)	4.31 (−36.04 to 44.67)
Gabon	12.90 (9.30−16.51)	17.18 (12.37−21.99)	33.12 (−12.60 to 79.91)	725.13 (522.65−928.19)	713.55 (513.75−913.51)	−1.60 (−40.76 to 37.47)	688.92 (496.54−881.74)	715.52 (515.18−916.01)	3.86 (−36.39 to 44.13)
Georgia	15.23 (10.95−19.51)	15.59 (11.22−19.95)	2.40 (−37.62 to 42.34)	408.01 (293.17−522.96)	439.05 (315.96−561.66)	7.61 (−33.48 to 48.67)	419.38 (301.59−537.29)	463.25 (333.38−592.84)	10.46 (−31.10 to 52.21)
Grenada	0.59 (0.43−0.76)	0.64 (0.46−0.81)	7.38 (−33.60 to 48.34)	517.75 (372.40−662.86)	545.46 (392.94−698.16)	5.35 (−35.20 to 45.94)	535.34 (384.99−685.37)	583.84 (420.66−747.33)	9.06 (−32.19 to 50.48)
Guatemala	95.55 (68.67−122.49)	128.97 (92.81−165.21)	34.97 (−11.29 to 82.33)	511.20 (367.68−654.96)	521.22 (375.09−667.75)	1.96 (−38.02 to 41.87)	510.91 (367.21−654.96)	527.17 (379.39−675.31)	3.18 (−37.08 to 43.36)
Indonesia	584.61 (421.56−746.87)	697.60 (501.89−892.49)	19.33 (−23.75 to 62.81)	221.46 (159.67−282.96)	231.82 (166.88−296.45)	4.68 (−35.56 to 44.95)	215.04 (155.07−274.73)	232.48 (167.26−297.43)	8.11 (−32.80 to 49.19)
Iran (Islamic Republic of)	269.74 (194.49−345.32)	311.12 (223.75−398.26)	15.34 (−27.14 to 58.08)	321.49 (231.78−411.65)	325.70 (234.46−416.86)	1.31 (−38.39 to 40.99)	310.42 (223.83−397.41)	326.50 (234.80−417.94)	5.18 (−35.29 to 45.67)
Iraq	146.69 (105.85−187.48)	238.33 (171.55−305.19)	62.47 (9.60−115.97)	281.45 (202.91−359.95)	289.39 (208.27−370.58)	2.82 (−37.20 to 42.75)	292.57 (211.11−373.93)	293.79 (211.47−376.21)	0.42 (−39.02 to 39.84)
Jamaica	13.36 (9.61−17.12)	12.57 (9.04−16.12)	−5.93 (−44.35 to 32.68)	474.03 (340.84−607.12)	482.39 (346.98−618.39)	1.76 (−38.21 to 41.66)	480.12 (345.19−614.94)	513.47 (369.16−658.23)	6.95 (−34.12 to 47.99)
Kazakhstan	79.55 (57.13−101.91)	104.96 (75.60−134.22)	31.94 (−13.65 to 78.49)	421.92 (302.99−540.47)	426.33 (307.09−545.24)	1.04 (−38.64 to 40.69)	414.56 (297.71−531.06)	434.43 (312.94−555.54)	4.79 (−35.70 to 45.30)
Libya	21.17 (15.26−27.11)	24.05 (17.32−30.78)	13.58 (−28.54 to 55.90)	286.45 (206.41−366.73)	273.96 (197.36−350.72)	−4.36 (−42.96 to 34.32)	284.80 (205.24−364.62)	267.82 (192.90−342.82)	−5.96 (−44.20 to 32.46)
Malaysia	113.08 (81.50−144.49)	161.48 (116.42−206.52)	42.80 (−4.97 to 91.57)	352.90 (254.30−450.99)	367.69 (265.17−470.08)	4.19 (−35.96 to 44.38)	340.67 (245.53−435.28)	385.49 (277.93−493.00)	13.16 (−28.72 to 55.19)
Maldives	1.63 (1.17−2.09)	2.55 (1.84−3.27)	56.86 (5.30−109.21)	340.87 (245.28−437.18)	353.04 (254.45−451.81)	3.57 (−36.68 to 43.73)	322.18 (231.93−413.07)	388.88 (280.23−497.63)	20.70 (−22.80 to 64.62)
Marshall Islands	0.16 (0.11−0.20)	0.20 (0.15−0.26)	28.85 (−16.08 to 74.67)	262.63 (188.70−336.28)	261.08 (188.40−333.97)	−0.59 (−39.95 to 38.67)	258.12 (185.43−330.50)	259.80 (187.48−332.33)	0.65 (−38.88 to 40.14)
Mauritius	9.97 (7.20−12.73)	11.25 (8.07−14.41)	12.77 (−29.13 to 54.97)	760.17 (548.16−970.86)	860.18 (617.78−1101.86)	13.16 (−28.81 to 55.44)	774.12 (558.73−988.03)	932.74 (669.43−1195.33)	20.49 (−22.98 to 64.41)
Mexico	969.17 (696.81−1242.64)	1252.83 (900.96−1605.00)	29.27 (−15.98 to 75.39)	715.92 (514.79−917.89)	713.19 (512.95−913.52)	−0.38 (−39.88 to 39.18)	708.66 (509.51−908.63)	748.72 (538.43−959.18)	5.65 (−35.09 to 46.43)

Location	Number of cases			ASPR			All age prevalence		
	2023 (thousand)	2050 (thousand)	Percent change (%)	2023 (per 100,000)	2050 (per 100,000)	Percent change (%)	2023 (per 100,000)	2050 (per 100,000)	Percent change (%)
Mongolia	12.00 (8.63−15.36)	17.08 (12.29−21.89)	42.38 (−5.65 to 91.39)	347.78 (250.28−445.17)	377.84 (271.74−484.06)	8.64 (−32.59 to 50.04)	338.77 (243.71−433.73)	377.58 (271.57−483.77)	11.46 (−30.37 to 53.42)
Montenegro	1.97 (1.42−2.52)	1.89 (1.36−2.42)	−4.09 (−42.85 to 34.75)	314.19 (226.10−402.49)	317.80 (228.39−406.89)	1.15 (−38.64 to 40.92)	314.46 (226.50−402.62)	319.91 (229.97−409.69)	1.73 (−38.15 to 41.52)
Namibia	10.46 (7.54−13.36)	16.51 (11.91−21.12)	57.80 (6.10−110.14)	418.42 (301.76−534.57)	441.07 (318.18−564.31)	5.41 (−34.89 to 45.72)	399.09 (287.79−509.84)	439.02 (316.70−561.70)	10.01 (−31.20 to 51.39)
North Macedonia	7.20 (5.18−9.22)	6.82 (4.91−8.74)	−5.28 (−43.75 to 33.32)	327.71 (235.67−419.38)	332.96 (239.65−426.12)	1.60 (−38.19 to 41.37)	328.79 (236.58−420.77)	338.87 (243.72−434.01)	3.06 (−37.10 to 43.14)
Paraguay	32.39 (23.34−41.41)	45.41 (32.68−58.14)	40.19 (−7.20 to 88.53)	439.08 (316.48−561.21)	478.48 (344.35−612.76)	8.97 (−32.20 to 50.27)	430.95 (310.62−550.92)	493.02 (354.83−631.33)	14.40 (−27.82 to 56.90)
Peru	204.37 (147.47−261.20)	276.05 (198.75−353.21)	35.08 (−10.96 to 82.13)	564.83 (407.57−721.93)	542.00 (390.22−693.58)	−4.04 (−42.64 to 34.61)	556.32 (401.43−711.00)	561.25 (404.09−718.12)	0.89 (−38.60 to 40.36)
Republic of Moldova	10.84 (7.80−13.86)	9.63 (6.93−12.34)	−11.14 (−48.31 to 26.34)	291.09 (209.61−372.46)	306.00 (220.18−392.18)	5.12 (−35.38 to 45.65)	296.51 (213.54−379.34)	320.95 (230.98−411.25)	8.25 (−32.86 to 49.52)
Saint Lucia	1.11 (0.80−1.42)	1.13 (0.81−1.45)	1.82 (−37.91 to 41.43)	605.01 (436.44−773.53)	610.24 (439.73−780.61)	0.86 (−38.60 to 40.32)	611.69 (441.29−782.05)	612.03 (440.64−783.31)	0.06 (−39.28 to 39.37)
Saint Vincent and the Grenadines	0.59 (0.42−0.75)	0.55 (0.39−0.70)	−7.29 (−45.19 to 30.74)	509.75 (367.51−651.67)	522.94 (377.04−669.12)	2.59 (−37.26 to 42.35)	517.56 (373.19−661.58)	549.96 (396.33−703.83)	6.26 (−34.34 to 46.80)
Serbia	32.64 (23.58−41.71)	27.92 (20.11−35.76)	−14.45 (−50.87 to 22.28)	363.04 (262.30−463.73)	358.94 (258.96−458.99)	−1.13 (−40.05 to 37.83)	378.86 (273.67−484.06)	385.97 (277.90−494.26)	1.88 (−37.82 to 41.49)
South Africa	220.84 (158.73−282.90)	282.61 (203.56−361.50)	27.97 (−16.91 to 73.67)	384.26 (276.26−492.14)	372.82 (268.50−477.02)	−2.98 (−41.95 to 36.04)	373.15 (268.20−478.01)	383.17 (275.99−490.13)	2.68 (−37.36 to 42.63)
Suriname	3.21 (2.31−4.12)	3.91 (2.82−4.99)	21.52 (−22.09 to 65.54)	537.76 (386.58−689.49)	570.16 (411.57−728.80)	6.02 (−34.61 to 46.74)	536.62 (385.79−688.06)	589.05 (425.16−753.05)	9.77 (−31.59 to 51.30)
Thailand	296.15 (213.38−378.71)	344.19 (247.70−440.13)	16.22 (−26.27 to 59.07)	405.51 (292.05−518.86)	433.68 (312.45−554.79)	6.95 (−33.84 to 47.70)	413.33 (297.81−528.57)	520.58 (374.64−665.69)	25.95 (−18.46 to 71.12)
Tonga	0.28 (0.20−0.35)	0.42 (0.31−0.54)	53.32 (2.77−104.59)	246.99 (178.53−315.33)	246.29 (177.32−315.14)	−0.28 (−39.44 to 38.86)	244.89 (177.04−312.66)	245.01 (176.40−313.48)	0.05 (−39.17 to 39.25)
Turkey	380.73 (274.14−487.59)	537.36 (387.25−687.40)	41.14 (−6.38 to 89.64)	443.78 (319.54−568.26)	475.03 (341.73−608.22)	7.04 (−33.91 to 47.98)	439.03 (316.11−562.24)	496.61 (357.88−635.26)	13.12 (−28.83 to 55.25)
Turkmenistan	30.08 (21.66−38.50)	44.52 (32.04−56.91)	47.99 (−1.20 to 97.92)	558.83 (402.34−715.13)	639.76 (460.45−817.95)	14.48 (−27.74 to 56.98)	556.09 (400.38−711.79)	636.80 (458.39−814.12)	14.51 (−27.72 to 57.02)
Ukraine	85.79 (61.81−109.69)	62.84 (45.27−80.48)	−26.74 (−61.19 to 8.01)	200.33 (144.29−256.14)	190.52 (137.22−243.92)	−4.90 (−43.36 to 33.65)	200.30 (144.31−256.09)	189.29 (136.35−242.42)	−5.49 (−43.81 to 32.96)
**L-MICs**									
Angola	185.80 (133.80−237.75)	374.00 (269.56−478.80)	101.29 (39.22−164.53)	586.35 (422.58−749.90)	627.71 (452.19−803.91)	7.05 (−33.78 to 47.94)	549.04 (395.37−702.55)	612.47 (441.44−784.09)	11.55 (−30.16 to 53.39)

Location	Number of cases			ASPR			All age prevalence		
	2023 (thousand)	2050 (thousand)	Percent change (%)	2023 (per 100,000)	2050 (per 100,000)	Percent change (%)	2023 (per 100,000)	2050 (per 100,000)	Percent change (%)
Bangladesh	365.32 (263.28−467.10)	366.76 (264.16−469.14)	0.39 (−39.01 to 39.77)	220.72 (159.08−282.20)	220.52 (158.97−281.86)	−0.09 (−39.32 to 39.09)	222.05 (160.03−283.92)	215.81 (155.44−276.05)	−2.81 (−41.67 to 36.02)
Benin	51.79 (37.26−66.27)	88.66 (63.84−113.36)	71.19 (16.58−126.71)	378.18 (271.87−484.18)	371.46 (267.38−475.06)	−1.78 (−40.96 to 37.34)	375.98 (270.50−481.14)	367.08 (264.32−469.35)	−2.37 (−41.38 to 36.62)
Bhutan	2.98 (2.15−3.82)	3.79 (2.73−4.85)	27.05 (−17.67 to 72.58)	302.21 (217.51−386.93)	313.92 (225.85−401.99)	3.87 (−36.46 to 44.22)	289.68 (208.56−370.88)	318.92 (229.52−408.35)	10.09 (−31.36 to 51.73)
Bolivia (Plurinational State of)	60.41 (43.49−77.35)	86.11 (62.04−110.11)	42.55 (−5.32 to 91.33)	484.45 (348.91−620.19)	459.70 (331.18−587.87)	−5.11 (−43.49 to 33.41)	467.03 (336.24−598.03)	459.90 (331.33−588.11)	−1.53 (−40.70 to 37.56)
Cabo Verde	2.59 (1.86−3.31)	3.44 (2.48−4.41)	33.08 (−12.69 to 79.90)	455.05 (327.02−582.78)	479.99 (345.99−614.14)	5.48 (−35.07 to 46.12)	445.68 (320.28−570.78)	507.58 (365.90−649.47)	13.89 (−28.32 to 56.25)
Cambodia	45.60 (32.80−58.42)	58.83 (42.32−75.30)	29.03 (−16.06 to 74.99)	265.55 (191.12−340.20)	282.35 (203.05−361.43)	6.33 (−34.46 to 47.08)	261.23 (187.92−334.67)	285.24 (205.18−365.07)	9.19 (−32.15 to 50.69)
Cameroon	113.14 (81.38−145.14)	160.78 (116.02−205.56)	42.11 (−5.60 to 90.81)	361.26 (259.96−463.02)	361.00 (260.38−461.67)	−0.07 (−39.49 to 39.32)	356.25 (256.24−457.01)	350.02 (252.58−447.50)	−1.75 (−40.94 to 37.45)
Comoros	3.32 (2.38−4.26)	4.13 (2.97−5.28)	24.29 (−20.17 to 69.24)	431.83 (310.15−553.73)	426.34 (306.61−545.66)	−1.27 (−40.70 to 38.06)	424.19 (304.47−544.18)	423.66 (304.77−542.09)	−0.12 (−39.74 to 39.43)
Congo	28.00 (20.18−35.84)	35.63 (25.68−45.59)	27.24 (−17.38 to 72.69)	543.41 (391.99−695.51)	538.77 (388.42−689.35)	−0.85 (−40.02 to 38.30)	520.94 (375.48−666.79)	537.50 (387.47−687.80)	3.18 (−36.87 to 43.10)
Côte d'Ivoire	118.87 (85.66−152.10)	199.66 (143.82−255.42)	67.97 (13.80−122.61)	416.46 (299.95−533.03)	416.39 (299.89−532.70)	−0.02 (−39.39 to 39.31)	407.99 (294.01−522.06)	412.87 (297.38−528.16)	1.19 (−38.42 to 40.80)
Djibouti	5.79 (4.17−7.41)	7.55 (5.43−9.67)	30.46 (−14.82 to 76.71)	484.74 (349.17−620.31)	484.04 (348.11−619.88)	−0.14 (−39.57 to 39.25)	460.88 (332.09−589.65)	483.58 (347.89−619.23)	4.93 (−35.50 to 45.38)
Egypt	284.24 (204.80−363.86)	413.20 (297.30−528.89)	45.37 (−3.24 to 94.79)	257.01 (185.11−329.01)	276.88 (199.22−354.39)	7.73 (−33.31 to 48.84)	265.31 (191.16−339.63)	275.28 (198.07−352.36)	3.76 (−36.49 to 44.02)
Eswatini	4.68 (3.38−5.98)	6.48 (4.67−8.29)	38.42 (−8.31 to 86.23)	398.57 (287.33−509.56)	408.12 (294.03−522.12)	2.40 (−37.43 to 42.14)	377.96 (272.53−483.13)	397.26 (286.18−508.21)	5.11 (−35.20 to 45.45)
Ghana	123.83 (88.94−158.73)	193.28 (139.26−247.19)	56.09 (4.73−108.18)	379.44 (272.68−486.18)	403.73 (290.90−516.31)	6.40 (−34.33 to 47.16)	363.81 (261.32−466.35)	396.43 (285.62−506.99)	8.97 (−32.37 to 50.39)
Guinea	48.19 (34.69−61.70)	79.37 (57.18−101.60)	64.71 (11.35−118.73)	348.04 (250.61−445.54)	348.74 (251.08−446.54)	0.20 (−39.29 to 39.65)	349.78 (251.77−447.82)	346.85 (249.84−443.96)	−0.84 (−40.13 to 38.40)
Haiti	37.03 (26.58−47.42)	45.81 (32.96−58.68)	23.71 (−20.59 to 68.55)	290.36 (208.49−371.86)	284.88 (204.98−364.88)	−1.89 (−41.17 to 37.32)	286.31 (205.51−366.59)	285.56 (205.46−365.77)	−0.26 (−39.76 to 39.27)
Honduras	56.47 (40.63−72.34)	78.76 (56.73−100.76)	39.47 (−7.63 to 87.67)	540.88 (389.26−692.69)	530.25 (381.93−678.48)	−1.96 (−41.10 to 37.12)	531.19 (382.19−680.51)	535.11 (385.44−684.62)	0.74 (−38.86 to 40.30)
India	3623.22 (2607.47−4637.94)	4251.63 (3069.66−5438.20)	17.34 (−25.36 to 60.42)	253.07 (182.12−323.97)	266.26 (192.20−340.63)	5.21 (−35.22 to 45.72)	248.23 (178.64−317.75)	264.91 (191.27−338.84)	6.72 (−33.96 to 47.37)
Jordan	30.23 (21.84−38.64)	42.77 (30.78−54.75)	41.49 (−6.11 to 90.07)	247.57 (178.88−316.49)	239.81 (172.63−306.97)	−3.14 (−41.89 to 35.57)	252.01 (182.11−322.18)	239.38 (172.28−306.46)	−5.01 (−43.37 to 33.42)

Location	Number of cases			ASPR			All age prevalence		
	2023 (thousand)	2050 (thousand)	Percent change (%)	2023 (per 100,000)	2050 (per 100,000)	Percent change (%)	2023 (per 100,000)	2050 (per 100,000)	Percent change (%)
Kenya	217.07 (156.65−277.90)	308.70 (222.17−395.04)	42.22 (−5.60 to 91.00)	407.36 (293.73−521.51)	411.78 (296.31−527.06)	1.08 (−38.56 to 40.73)	397.76 (287.05−509.24)	397.16 (285.83−508.23)	−0.15 (−39.50 to 39.15)
Kiribati	0.38 (0.27−0.49)	0.64 (0.46−0.82)	68.06 (13.81−123.02)	291.72 (210.09−373.05)	303.72 (218.40−389.10)	4.12 (−36.19 to 44.50)	282.90 (203.74−361.72)	302.02 (217.14−386.97)	6.76 (−34.10 to 47.66)
Kyrgyzstan	24.47 (17.61−31.34)	36.06 (25.99−46.06)	47.35 (−1.51 to 97.07)	354.88 (255.62−454.28)	385.09 (277.55−492.00)	8.51 (−32.54 to 49.66)	351.23 (252.79−449.82)	383.87 (276.71−490.38)	9.29 (−31.90 to 50.65)
Lao People's Democratic Republic	20.17 (14.53−25.84)	26.91 (19.39−34.41)	33.37 (−12.37 to 80.18)	267.70 (192.76−342.90)	296.00 (213.23−378.62)	10.57 (−30.92 to 52.25)	265.62 (191.37−340.20)	293.21 (211.27−374.98)	10.38 (−31.03 to 51.98)
Lebanon	33.39 (24.08−42.68)	41.10 (29.50−52.70)	23.11 (−20.99 to 67.84)	362.27 (261.19−463.28)	366.58 (263.22−469.86)	1.19 (−38.57 to 40.85)	363.17 (261.90−464.21)	360.58 (258.77−462.37)	−0.71 (−40.07 to 38.62)
Lesotho	6.59 (4.75−8.42)	8.70 (6.25−11.15)	32.01 (−13.56 to 78.65)	327.02 (235.65−418.09)	354.93 (255.24−455.18)	8.53 (−32.66 to 49.85)	317.41 (228.78−405.58)	348.05 (250.30−446.32)	9.65 (−31.75 to 51.22)
Mauritania	16.53 (11.92−21.14)	25.89 (18.65−33.12)	56.62 (5.11−108.81)	366.56 (264.17−468.82)	353.12 (254.41−451.82)	−3.67 (−42.37 to 35.10)	362.63 (261.45−463.76)	345.86 (249.14−442.57)	−4.63 (−43.08 to 33.93)
Micronesia (Federated States of)	0.32 (0.23−0.41)	0.43 (0.31−0.55)	33.84 (−12.15 to 80.91)	287.82 (207.03−368.89)	288.93 (208.16−370.10)	0.38 (−39.22 to 39.94)	285.00 (205.00−365.22)	287.17 (206.89−367.86)	0.76 (−38.92 to 40.41)
Morocco	96.25 (69.33−123.29)	106.72 (76.90−136.55)	10.88 (−30.70 to 52.63)	257.29 (185.34−329.55)	256.68 (184.94−328.47)	−0.23 (−39.59 to 39.14)	256.10 (184.46−328.05)	251.46 (181.18−321.73)	−1.81 (−40.95 to 37.26)
Myanmar	148.81 (106.93−190.50)	191.39 (138.02−244.62)	28.61 (−16.23 to 74.35)	271.38 (195.00−347.41)	302.16 (217.92−386.13)	11.34 (−30.26 to 53.13)	267.03 (191.87−341.82)	301.40 (217.35−385.22)	12.87 (−29.03 to 54.93)
Nepal	126.09 (90.81−161.40)	159.66 (115.16−204.12)	26.62 (−17.89 to 71.89)	415.82 (299.50−532.10)	446.52 (321.99−570.97)	7.38 (−33.46 to 48.21)	396.50 (285.55−507.54)	465.64 (335.86−595.30)	17.44 (−25.28 to 60.51)
Nicaragua	29.30 (21.07−37.49)	35.59 (25.60−45.54)	21.47 (−22.21 to 65.59)	424.48 (305.37−543.20)	433.14 (311.50−554.42)	2.04 (−37.87 to 41.86)	424.84 (305.59−543.71)	449.19 (323.21−574.81)	5.73 (−34.86 to 46.34)
Nigeria	919.26 (662.56−1176.51)	1964.42 (1413.36−2517.65)	113.70 (48.37−180.40)	385.61 (277.73−493.69)	408.16 (293.56−523.15)	5.85 (−34.85 to 46.52)	371.21 (267.55−475.09)	400.79 (288.36−513.66)	7.97 (−33.09 to 49.21)
Pakistan	864.93 (620.91−1108.84)	1067.27 (770.48−1363.96)	23.39 (−20.62 to 67.86)	365.55 (262.51−468.40)	357.79 (258.28−457.26)	−2.12 (−41.21 to 37.00)	363.96 (261.27−466.59)	347.67 (250.99−444.32)	−4.47 (−43.03 to 34.27)
Palestine	17.11 (12.35−21.86)	24.13 (17.38−30.89)	41.01 (−6.47 to 89.41)	295.16 (212.79−377.07)	289.61 (208.55−370.72)	−1.88 (−40.87 to 37.03)	310.58 (224.05−396.63)	288.58 (207.80−369.41)	−7.08 (−45.00 to 30.98)
Papua New Guinea	21.39 (15.42−27.38)	35.10 (25.26−44.93)	64.14 (10.89−118.04)	208.34 (150.20−266.71)	204.09 (146.96−261.08)	−2.04 (−41.09 to 36.94)	201.43 (145.24−257.90)	201.26 (144.81−257.59)	−0.09 (−39.48 to 39.27)
Philippines	352.54 (253.40−451.66)	487.90 (350.70−624.97)	38.39 (−8.67 to 86.55)	312.56 (224.72−400.36)	320.07 (230.08−410.00)	2.40 (−37.69 to 42.39)	309.16 (222.22−396.09)	319.72 (229.81−409.54)	3.41 (−36.92 to 43.67)
Samoa	0.55 (0.40−0.70)	0.97 (0.70−1.24)	76.06 (20.12−132.76)	255.60 (184.28−326.99)	262.78 (188.99−336.26)	2.81 (−37.19 to 42.71)	246.54 (177.77−315.37)	260.24 (187.22−332.95)	5.56 (−34.91 to 46.01)
Sao Tome and Principe	0.97 (0.70−1.24)	1.29 (0.93−1.65)	33.20 (−12.36 to 79.82)	456.19 (328.69−583.09)	475.90 (343.03−608.75)	4.32 (−35.90 to 44.61)	444.34 (320.26−567.78)	470.67 (339.38−601.91)	5.93 (−34.51 to 46.32)

Location	Number of cases			ASPR			All age prevalence		
	2023 (thousand)	2050 (thousand)	Percent change (%)	2023 (per 100,000)	2050 (per 100,000)	Percent change (%)	2023 (per 100,000)	2050 (per 100,000)	Percent change (%)
Senegal	68.77 (49.54−87.98)	114.26 (82.45−146.06)	66.16 (12.76−120.30)	403.90 (290.78−516.83)	408.62 (294.79−522.41)	1.17 (−38.38 to 40.71)	401.53 (289.28−513.72)	407.46 (294.03−520.85)	1.48 (−38.10 to 41.05)
Solomon Islands	1.60 (1.15−2.04)	2.40 (1.73−3.08)	50.53 (0.72−101.03)	226.02 (162.92−288.55)	229.03 (165.05−293.01)	1.33 (−38.21 to 40.80)	222.21 (160.06−283.75)	230.40 (166.03−294.81)	3.68 (−36.40 to 43.80)
Sri Lanka	105.64 (76.02−135.28)	117.81 (84.83−150.57)	11.52 (−30.18 to 53.39)	470.78 (338.79−602.86)	472.17 (339.86−603.69)	0.29 (−39.17 to 39.72)	478.51 (344.34−612.76)	562.60 (405.12−719.03)	17.57 (−25.24 to 60.74)
Tajikistan	52.54 (37.89−67.07)	71.08 (51.21−90.99)	35.28 (−10.77 to 82.34)	502.12 (362.11−640.91)	441.64 (318.19−565.40)	−12.05 (−48.89 to 25.08)	501.05 (361.27−639.58)	442.58 (318.89−566.55)	−11.67 (−48.56 to 25.51)
Timor-Leste	3.29 (2.38−4.21)	4.89 (3.53−6.26)	48.51 (−0.66 to 98.41)	222.22 (160.34−284.07)	224.29 (161.81−286.79)	0.93 (−38.49 to 40.33)	228.29 (164.82−291.76)	221.36 (159.71−283.07)	−3.04 (−41.72 to 35.65)
Tunisia	35.28 (25.42−45.11)	42.19 (30.36−54.06)	19.58 (−23.67 to 63.28)	295.79 (213.17−378.20)	291.16 (209.54−373.00)	−1.56 (−40.68 to 37.51)	291.96 (210.40−373.31)	310.44 (223.43−397.77)	6.33 (−34.41 to 47.04)
United Republic of Tanzania	296.25 (213.51−379.37)	562.92 (404.97−720.44)	90.02 (30.59−150.49)	467.53 (337.01−598.12)	466.38 (335.58−596.84)	−0.25 (−39.58 to 39.04)	457.57 (329.78−585.96)	459.91 (330.87−588.61)	0.51 (−39.04 to 40.03)
Uzbekistan	150.50 (108.44−192.42)	185.17 (133.25−236.89)	23.04 (−20.90 to 67.49)	433.74 (312.46−554.56)	433.37 (311.84−554.43)	−0.08 (−39.42 to 39.22)	431.05 (310.60−551.11)	432.80 (311.45−553.68)	0.41 (−39.03 to 39.83)
Vanuatu	0.75 (0.54−0.97)	1.21 (0.87−1.55)	60.75 (7.94−114.22)	233.95 (168.11−299.75)	235.21 (169.16−301.26)	0.54 (−39.15 to 40.19)	230.86 (165.93−295.80)	235.00 (168.97−301.01)	1.79 (−38.18 to 41.72)
Viet Nam	203.72 (146.71−260.67)	242.82 (174.84−310.56)	19.20 (−23.90 to 62.65)	207.90 (149.76−265.98)	223.97 (161.27−286.53)	7.73 (−33.23 to 48.76)	201.86 (145.37−258.29)	228.02 (164.18−291.63)	12.96 (−28.95 to 55.03)
Zambia	125.77 (90.68−161.01)	222.00 (160.06−283.96)	76.51 (20.56−133.13)	620.97 (447.96−794.56)	628.22 (452.75−803.63)	1.17 (−38.40 to 40.72)	609.04 (439.14−779.68)	617.79 (445.43−790.20)	1.44 (−38.20 to 41.06)
Zimbabwe	65.23 (46.90−83.49)	105.20 (75.70−134.56)	61.27 (8.54−114.65)	392.22 (281.75−502.26)	392.15 (282.23−501.46)	−0.02 (−39.47 to 39.48)	384.73 (276.61−492.42)	387.83 (279.08−496.07)	0.81 (−38.80 to 40.39)
**LICs**									
Afghanistan	89.05 (64.24−113.74)	181.80 (130.65−232.71)	104.15 (41.41−168.18)	201.96 (145.63−257.88)	202.32 (145.31−259.19)	0.18 (−39.26 to 39.60)	222.67 (160.63−284.40)	216.84 (155.83−277.56)	−2.62 (−41.51 to 36.18)
Burkina Faso	85.82 (62.08−109.56)	173.79 (125.23−222.20)	102.49 (40.46−165.90)	352.97 (254.95−450.70)	358.24 (258.11−458.09)	1.49 (−38.02 to 40.91)	339.11 (245.30−432.88)	349.79 (252.05−447.24)	3.15 (−36.67 to 42.99)
Burundi	42.35 (30.47−54.21)	85.32 (61.29−109.29)	101.48 (39.08−165.07)	334.25 (240.72−427.53)	331.56 (238.19−424.95)	−0.80 (−40.14 to 38.49)	323.75 (232.93−414.49)	323.24 (232.20−414.06)	−0.16 (−39.67 to 39.32)
Central African Republic	17.91 (12.93−22.88)	19.19 (13.85−24.51)	7.11 (−33.48 to 47.68)	383.09 (276.36−489.56)	387.84 (279.86−495.52)	1.24 (−38.19 to 40.66)	370.92 (267.75−473.77)	379.68 (274.02−484.95)	2.36 (−37.26 to 41.87)
Chad	57.40 (41.20−73.59)	158.92 (114.36−203.44)	176.85 (95.54−259.84)	306.99 (220.57−393.47)	326.28 (234.88−417.37)	6.28 (−34.48 to 47.07)	300.50 (215.70−385.25)	322.81 (232.31−413.25)	7.43 (−33.70 to 48.50)
Democratic People's Republic of Korea	48.54 (34.96−62.13)	44.04 (31.69−56.42)	−9.27 (−46.81 to 28.70)	189.44 (136.38−242.47)	188.43 (135.56−241.39)	−0.53 (−39.90 to 38.81)	186.89 (134.58−239.19)	187.11 (134.64−239.70)	0.12 (−39.38 to 39.59)

Location	Number of cases			ASPR			All age prevalence		
	2023 (thousand)	2050 (thousand)	Percent change (%)	2023 (per 100,000)	2050 (per 100,000)	Percent change (%)	2023 (per 100,000)	2050 (per 100,000)	Percent change (%)
Democratic Republic of the Congo	348.66 (251.11−445.57)	595.30 (429.18−762.00)	70.74 (16.24−126.11)	372.05 (267.95−475.48)	357.56 (257.69−457.71)	−3.89 (−42.55 to 34.83)	364.05 (262.19−465.23)	352.29 (253.99−450.95)	−3.23 (−42.00 to 35.56)
Eritrea	26.04 (18.75−33.33)	34.50 (24.85−44.16)	32.49 (−13.12 to 79.15)	411.24 (296.12−526.06)	410.11 (295.28−524.94)	−0.28 (−39.61 to 39.02)	397.19 (285.96−508.28)	392.55 (282.73−502.39)	−1.17 (−40.39 to 37.97)
Ethiopia	350.20 (252.71−448.03)	601.21 (434.18−767.68)	71.68 (17.28−126.96)	298.35 (215.19−381.64)	303.42 (219.07−387.41)	1.70 (−37.82 to 41.21)	285.84 (206.26−365.69)	292.90 (211.52−373.99)	2.47 (−37.22 to 42.08)
Gambia	7.85 (5.65−10.04)	11.56 (8.32−14.81)	47.14 (−1.97 to 97.01)	319.85 (230.24−408.87)	319.47 (230.04−409.20)	−0.12 (−39.50 to 39.23)	320.96 (230.97−410.16)	314.48 (226.40−402.87)	−2.02 (−41.11 to 36.97)
Guinea-Bissau	7.82 (5.65−9.98)	11.98 (8.62−15.33)	53.13 (2.59−104.39)	371.67 (268.09−474.84)	361.72 (260.31−463.09)	−2.68 (−41.55 to 36.11)	362.27 (261.52−462.42)	358.79 (258.33−459.28)	−0.96 (−40.01 to 38.04)
Liberia	18.59 (13.39−23.76)	29.25 (21.05−37.43)	57.35 (5.60−109.75)	346.82 (249.83−443.57)	366.06 (263.53−468.44)	5.55 (−34.93 to 46.03)	342.07 (246.50−437.29)	362.57 (261.01−463.98)	5.99 (−34.57 to 46.52)
Madagascar	106.98 (76.91−136.88)	206.40 (148.37−264.30)	92.93 (32.81−154.09)	344.66 (247.88−440.85)	336.20 (241.81−430.31)	−2.45 (−41.50 to 36.54)	341.08 (245.20−436.40)	333.72 (239.90−427.34)	−2.16 (−41.32 to 36.93)
Malawi	85.74 (61.82−109.72)	136.99 (98.27−175.69)	59.78 (7.08−113.09)	431.74 (311.24−552.14)	430.93 (309.25−552.63)	−0.19 (−39.69 to 39.30)	423.44 (305.32−541.87)	414.85 (297.58−532.04)	−2.03 (−41.20 to 37.12)
Mali	61.80 (44.44−79.17)	143.48 (103.31−183.57)	132.18 (62.52−203.21)	250.47 (180.24−320.66)	273.65 (196.89−350.18)	9.25 (−32.05 to 50.69)	250.90 (180.43−321.43)	273.62 (197.01−350.07)	9.05 (−32.22 to 50.51)
Mozambique	160.54 (115.38−205.62)	233.94 (168.49−299.31)	45.73 (−2.93 to 95.17)	493.82 (354.86−632.65)	490.34 (353.06−627.34)	−0.70 (−40.11 to 38.62)	465.59 (334.64−596.34)	461.59 (332.45−590.57)	−0.86 (−40.23 to 38.42)
Niger	72.99 (52.47−93.37)	202.06 (145.32−258.89)	176.86 (95.42−260.02)	269.57 (193.87−344.89)	268.10 (192.90−343.36)	−0.54 (−39.90 to 38.81)	268.47 (193.01−343.46)	268.37 (193.00−343.85)	−0.04 (−39.55 to 39.43)
Rwanda	57.22 (41.26−73.13)	96.36 (69.23−123.57)	68.39 (14.13−123.55)	398.00 (286.82−508.79)	383.67 (275.72−491.93)	−3.60 (−42.45 to 35.22)	389.41 (280.77−497.66)	378.74 (272.09−485.70)	−2.74 (−41.73 to 36.16)
Sierra Leone	38.74 (27.82−49.71)	54.95 (39.65−70.17)	41.86 (−5.69 to 90.46)	426.78 (306.83−547.37)	425.09 (306.63−542.93)	−0.40 (−39.81 to 38.92)	430.23 (309.03−552.10)	415.78 (300.01−530.93)	−3.36 (−42.17 to 35.58)
Somalia	58.82 (42.29−75.43)	110.70 (79.80−141.50)	88.21 (29.43−148.10)	297.91 (214.42−381.72)	293.01 (211.13−374.58)	−1.65 (−40.82 to 37.47)	292.41 (210.22−374.96)	287.30 (207.09−367.23)	−1.75 (−40.95 to 37.45)
South Sudan	38.54 (27.73−49.39)	81.30 (58.49−103.94)	110.91 (46.57−176.55)	324.71 (233.48−416.05)	301.02 (216.58−385.00)	−7.30 (−45.38 to 30.97)	317.45 (228.35−406.78)	294.46 (211.87−376.47)	−7.24 (−45.31 to 31.01)
Sudan	112.05 (80.57−143.63)	155.33 (112.01−198.44)	38.62 (−8.19 to 86.53)	219.15 (157.67−280.76)	222.23 (160.23−283.95)	1.41 (−38.25 to 41.03)	241.00 (173.27−308.91)	220.52 (159.01−281.73)	−8.50 (−46.14 to 29.55)
Syrian Arab Republic	39.86 (28.67−51.07)	40.19 (28.88−51.52)	0.83 (−38.96 to 40.62)	211.25 (152.04−270.49)	205.21 (147.46−263.25)	−2.86 (−41.98 to 36.20)	217.16 (156.19−278.24)	201.39 (144.75−258.18)	−7.26 (−45.48 to 31.08)
Togo	32.30 (23.18−41.41)	42.05 (30.26−53.86)	30.20 (−15.23 to 76.54)	387.19 (278.00−496.34)	364.82 (262.64−467.25)	−5.78 (−44.28 to 32.86)	379.85 (272.65−487.08)	358.24 (257.83−458.85)	−5.69 (−44.24 to 33.00)
Uganda	257.44 (186.18−328.30)	494.13 (356.41−631.78)	91.94 (32.65−152.29)	551.05 (397.93−703.45)	573.95 (414.00−733.79)	4.16 (−35.84 to 44.23)	541.56 (391.66−690.62)	557.78 (402.31−713.15)	3.00 (−36.72 to 42.73)

Location	Number of cases			ASPR			All age prevalence		
	2023 (thousand)	2050 (thousand)	Percent change (%)	2023 (per 100,000)	2050 (per 100,000)	Percent change (%)	2023 (per 100,000)	2050 (per 100,000)	Percent change (%)
Yemen	66.81 (48.05−85.48)	88.63 (63.98−113.30)	32.66 (−12.87 to 79.20)	173.70 (124.91−222.35)	170.59 (123.14−218.09)	−1.79 (−40.87 to 37.26)	188.70 (135.73−241.43)	169.12 (122.08−216.20)	−10.38 (−47.58 to 27.13)
**Secondary epilepsy**									
**U-MICs**									
Albania	9.64 (8.41−10.88)	9.18 (8.04−10.32)	−4.79 (−22.15 to 12.63)	363.34 (316.98−409.82)	385.24 (337.65−432.87)	6.03 (−12.22 to 24.23)	341.83 (298.12−385.63)	336.18 (294.45−377.94)	−1.65 (−19.29 to 16.02)
Algeria	114.36 (97.93−130.80)	179.67 (154.77−204.54)	57.11 (31.36−83.22)	243.97 (208.97−279.02)	311.09 (267.95−354.20)	27.51 (4.93−50.34)	252.90 (216.58−289.27)	292.86 (252.28−333.41)	15.80 (−5.63 to 37.30)
Argentina	174.04 (147.30−200.81)	212.39 (179.52−245.18)	22.04 (−2.09 to 46.42)	373.25 (316.17−430.39)	404.82 (343.34−466.18)	8.46 (−13.93 to 30.88)	372.38 (315.17−429.66)	394.61 (333.54−455.55)	5.97 (−16.42 to 28.33)
Armenia	13.03 (11.12−14.94)	13.16 (11.23−15.10)	0.99 (−19.80 to 21.77)	431.43 (369.14−493.62)	488.04 (418.97−557.19)	13.12 (−8.34 to 34.67)	428.94 (366.00−491.72)	467.96 (399.15−536.83)	9.10 (−12.53 to 30.79)
Azerbaijan	37.45 (32.32−42.57)	39.78 (34.57−45.00)	6.23 (−13.23 to 25.66)	342.96 (295.98−389.97)	386.61 (335.68−437.54)	12.73 (−7.39 to 32.92)	348.89 (301.13−396.65)	348.42 (302.75−394.11)	−0.13 (−19.01 to 18.79)
Belarus	32.70 (27.90−37.52)	26.78 (22.85−30.72)	−18.10 (−37.03 to 0.93)	346.89 (297.67−396.21)	341.04 (293.78−388.30)	−1.69 (−21.30 to 17.96)	349.91 (298.58−401.44)	331.08 (282.45−379.76)	−5.38 (−25.53 to 14.84)
Belize	2.49 (2.10−2.88)	3.55 (2.99−4.10)	42.48 (15.60−69.93)	568.45 (477.94−659.14)	607.45 (513.51−701.18)	6.86 (−16.03 to 29.72)	567.53 (478.62−656.69)	612.84 (516.38−709.09)	7.98 (−15.04 to 31.09)
Bosnia and Herzegovina	17.94 (15.37−20.52)	15.07 (12.92−17.22)	−15.99 (−34.57 to 2.74)	542.37 (467.10−617.77)	583.16 (503.88−662.30)	7.52 (−12.56 to 27.62)	537.14 (460.21−614.25)	548.33 (469.94−626.47)	2.08 (−18.29 to 22.43)
Botswana	14.65 (12.35−16.94)	24.61 (20.78−28.44)	68.04 (37.80−98.52)	601.80 (504.44−698.97)	760.73 (644.13−877.41)	26.41 (1.41−51.64)	584.36 (492.73−675.98)	752.93 (635.68−870.12)	28.85 (3.77−54.42)
Brazil	1085.53 (906.92−1264.15)	1571.57 (1307.22−1835.62)	44.77 (15.82−74.17)	486.84 (407.17−566.48)	678.19 (567.38−788.82)	39.31 (11.84−67.40)	490.19 (409.53−570.84)	671.15 (558.26−783.91)	36.92 (9.15−65.29)
China	3524.99 (2975.41−4076.67)	3647.86 (3090.58−4206.76)	3.49 (−18.67 to 25.64)	245.91 (208.77−283.18)	309.73 (265.61−353.99)	25.96 (2.68−49.41)	246.28 (207.88−284.82)	286.31 (242.57−330.17)	16.25 (−7.29 to 39.91)
Colombia	239.25 (201.50−277.04)	318.09 (267.24−368.93)	32.95 (7.01−59.51)	442.85 (373.24−512.53)	531.16 (448.28−614.09)	19.94 (−4.28 to 44.37)	444.07 (374.00−514.21)	517.66 (434.91−600.39)	16.57 (−7.67 to 40.94)
Costa Rica	22.73 (18.98−26.48)	28.21 (23.42−33.00)	24.08 (−2.27 to 50.96)	453.19 (378.84−527.41)	467.22 (391.32−542.96)	3.10 (−20.25 to 26.39)	460.70 (384.67−536.57)	514.63 (427.21−602.04)	11.70 (−13.26 to 36.84)
Cuba	27.71 (23.92−31.50)	20.33 (17.63−23.03)	−26.61 (−43.28 to −9.73)	268.66 (231.71−305.64)	276.95 (239.83−313.96)	3.09 (−16.32 to 22.47)	245.64 (212.02−279.26)	215.73 (187.04−244.34)	−12.17 (−30.01 to 5.83)
Dominica	0.44 (0.36−0.51)	0.43 (0.36−0.50)	−1.32 (−25.02 to 22.41)	616.20 (513.24−719.10)	618.45 (516.40−720.58)	0.36 (−23.06 to 23.79)	625.15 (519.54−730.70)	636.77 (528.44−745.08)	1.86 (−22.26 to 25.93)
Dominican Republic	65.99 (55.22−76.80)	78.50 (65.66−91.36)	18.95 (−6.24 to 44.35)	598.43 (500.16−697.02)	639.68 (537.40−742.16)	6.89 (−16.78 to 30.58)	596.15 (498.84−693.77)	650.46 (544.07−757.02)	9.11 (−14.99 to 33.28)

Location	Number of cases			ASPR			All age prevalence		
	2023 (thousand)	2050 (thousand)	Percent change (%)	2023 (per 100,000)	2050 (per 100,000)	Percent change (%)	2023 (per 100,000)	2050 (per 100,000)	Percent change (%)
Ecuador	80.11 (66.60−93.61)	109.14 (91.44−126.91)	36.23 (8.88−64.19)	455.44 (377.82−532.98)	484.20 (408.16−560.42)	6.32 (−17.50 to 30.07)	445.55 (370.40−520.64)	504.75 (422.86−586.91)	13.29 (−11.56 to 38.21)
El Salvador	29.76 (24.91−34.60)	29.08 (24.29−33.86)	−2.29 (−25.10 to 20.48)	475.58 (397.89−553.31)	497.18 (418.40−575.89)	4.54 (−18.64 to 27.71)	478.10 (400.29−555.96)	514.93 (430.13−599.72)	7.70 (−16.26 to 31.76)
Equatorial Guinea	8.06 (6.90−9.22)	19.12 (16.33−21.93)	137.23 (100.25−174.97)	574.98 (483.94−666.17)	761.03 (647.92−874.37)	32.36 (7.32−57.74)	506.40 (433.50−579.48)	741.99 (633.49−850.73)	46.52 (21.04−72.39)
Fiji	3.27 (2.77−3.78)	3.93 (3.32−4.54)	20.05 (−3.93 to 44.26)	336.28 (283.79−388.59)	365.16 (308.66−421.65)	8.59 (−14.25 to 31.47)	344.73 (291.29−398.02)	360.44 (304.59−416.28)	4.56 (−17.78 to 26.93)
Gabon	12.06 (10.22−13.89)	18.92 (16.03−21.81)	56.91 (28.72−85.45)	676.18 (569.25−782.98)	793.34 (672.81−913.89)	17.33 (−6.34 to 41.09)	643.74 (545.83−741.48)	788.05 (667.60−908.51)	22.42 (−1.48 to 46.58)
Georgia	15.72 (13.29−18.15)	17.26 (14.71−19.81)	9.77 (−12.54 to 32.12)	423.31 (359.77−486.76)	516.92 (443.96−589.81)	22.11 (−0.58 to 44.96)	433.05 (366.12−499.90)	512.77 (436.95−588.46)	18.41 (−4.80 to 41.70)
Grenada	0.67 (0.56−0.78)	0.69 (0.57−0.80)	2.73 (−21.05 to 26.44)	588.95 (492.35−685.39)	592.62 (498.04−687.21)	0.62 (−22.27 to 23.47)	606.83 (505.88−707.56)	633.12 (527.72−738.57)	4.33 (−19.64 to 28.35)
Guatemala	115.23 (96.39−133.98)	171.20 (143.38−199.02)	48.58 (19.86−77.73)	627.30 (522.55−731.65)	690.85 (580.00−801.76)	10.13 (−14.06 to 34.30)	616.12 (515.42−716.41)	699.80 (586.10−813.54)	13.58 (−10.91 to 38.16)
Indonesia	720.65 (621.89−819.79)	978.50 (843.92−1112.80)	35.78 (13.06−59.00)	258.39 (222.95−293.97)	359.90 (310.15−409.55)	39.29 (16.09−62.99)	265.09 (228.76−301.55)	326.09 (281.24−370.85)	23.01 (1.42−44.84)
Iran (Islamic Republic of)	243.68 (210.93−276.44)	326.28 (281.92−370.61)	33.90 (11.70−56.60)	276.66 (239.42−313.90)	392.18 (338.65−445.68)	41.76 (18.59−65.39)	280.43 (242.75−318.14)	342.41 (295.85−388.93)	22.10 (0.93−43.52)
Iraq	220.32 (185.95−254.72)	328.30 (277.26−379.20)	49.01 (21.50−76.94)	394.63 (333.32−455.97)	409.92 (346.21−473.44)	3.87 (−18.46 to 26.20)	439.43 (370.87−508.04)	404.69 (341.77−467.44)	−7.90 (−28.93 to 13.31)
Jamaica	18.02 (15.07−20.99)	16.11 (13.42−18.81)	−10.64 (−32.67 to 11.65)	642.70 (537.43−748.09)	656.35 (550.08−763.06)	2.12 (−21.09 to 25.33)	647.51 (541.24−753.90)	657.84 (548.01−768.09)	1.60 (−21.98 to 25.13)
Kazakhstan	73.69 (62.98−84.39)	105.31 (90.41−120.15)	42.92 (18.54−67.85)	380.73 (325.21−436.26)	443.92 (381.86−505.70)	16.59 (−5.15 to 38.40)	384.01 (328.22−439.80)	435.91 (374.22−497.31)	13.51 (−8.00 to 35.12)
Libya	19.36 (16.63−22.08)	21.31 (18.21−24.40)	10.08 (−11.10 to 31.35)	247.86 (212.99−282.73)	258.30 (220.69−295.89)	4.21 (−16.41 to 24.87)	260.35 (223.69−297.01)	237.28 (202.82−271.69)	−8.86 (−28.11 to 10.46)
Malaysia	124.46 (105.85−143.04)	151.27 (128.42−174.11)	21.54 (−1.93 to 45.25)	364.44 (309.92−418.88)	388.42 (329.54−447.28)	6.58 (−15.35 to 28.57)	374.94 (318.89−430.92)	361.11 (306.56−415.65)	−3.69 (−24.50 to 17.11)
Maldives	1.89 (1.61−2.16)	2.40 (2.07−2.73)	27.20 (4.59−50.04)	354.86 (302.97−406.50)	405.72 (349.13−462.48)	14.33 (−7.20 to 35.92)	373.46 (319.01−427.65)	365.55 (315.09−416.16)	−2.12 (−21.91 to 17.74)
Marshall Islands	0.21 (0.18−0.25)	0.27 (0.23−0.31)	26.48 (2.14−51.12)	327.63 (276.38−378.92)	339.78 (288.32−391.12)	3.71 (−18.36 to 25.75)	348.33 (294.50−402.17)	344.15 (292.17−396.01)	−1.20 (−22.63 to 20.26)
Mauritius	6.90 (5.78−8.02)	7.00 (5.83−8.16)	1.44 (−21.92 to 24.80)	559.47 (468.98−649.80)	671.14 (560.43−781.60)	19.96 (−5.35 to 45.53)	535.64 (448.58−622.57)	580.56 (483.82−677.00)	8.39 (−15.78 to 32.65)
Mexico	892.11 (747.93−1036.18)	1284.85 (1072.61−1497.29)	44.02 (15.69−72.79)	664.69 (556.38−772.90)	732.71 (615.09−850.38)	10.23 (−13.70 to 34.27)	652.32 (546.89−757.66)	767.85 (641.01−894.81)	17.71 (−7.34 to 43.03)

Location	Number of cases			ASPR			All age prevalence		
	2023 (thousand)	2050 (thousand)	Percent change (%)	2023 (per 100,000)	2050 (per 100,000)	Percent change (%)	2023 (per 100,000)	2050 (per 100,000)	Percent change (%)
Mongolia	16.66 (14.17−19.15)	32.16 (27.77−36.57)	93.08 (63.13−123.46)	471.76 (400.07−543.51)	721.49 (623.57−819.46)	52.93 (27.70−78.74)	470.42 (400.20−540.68)	711.00 (613.76−808.27)	51.14 (26.14−76.73)
Montenegro	2.70 (2.30−3.09)	2.42 (2.06−2.77)	−10.38 (−29.92 to 9.39)	435.98 (373.72−498.25)	426.32 (365.08−487.39)	−2.21 (−22.19 to 17.71)	429.88 (367.24−492.54)	408.66 (348.14−469.00)	−4.94 (−25.14 to 15.30)
Namibia	15.50 (13.05−17.95)	29.74 (25.20−34.28)	91.84 (58.97−125.33)	610.47 (510.40−710.25)	793.36 (671.95−914.63)	29.96 (4.44−55.73)	591.43 (497.70−684.91)	790.97 (670.11−911.67)	33.74 (8.30−59.66)
North Macedonia	10.16 (8.75−11.58)	8.92 (7.66−10.18)	−12.19 (−30.68 to 6.44)	469.79 (406.23−533.45)	474.24 (411.24−537.16)	0.95 (−18.03 to 19.90)	463.61 (399.08−528.23)	442.95 (380.59−505.27)	−4.46 (−23.78 to 14.89)
Paraguay	35.83 (30.24−41.43)	44.49 (37.46−51.54)	24.18 (−0.63 to 49.40)	481.10 (404.93−557.43)	479.49 (404.80−554.39)	−0.33 (−22.49 to 21.77)	476.72 (402.34−551.32)	483.09 (406.78−559.63)	1.34 (−20.98 to 23.60)
Peru	186.12 (154.97−217.18)	302.35 (254.65−350.11)	62.44 (32.22−93.13)	520.18 (432.30−607.83)	597.42 (505.44−689.57)	14.85 (−9.50 to 39.25)	506.65 (421.85−591.20)	614.70 (517.73−711.81)	21.33 (−3.92 to 46.76)
Republic of Moldova	14.78 (12.58−16.98)	11.59 (9.76−13.43)	−21.58 (−40.84 to −2.15)	408.03 (348.77−467.24)	405.66 (345.59−465.78)	−0.58 (−21.19 to 20.01)	404.49 (344.17−464.74)	386.42 (325.34−447.54)	−4.47 (−25.61 to 16.67)
Saint Lucia	1.43 (1.19−1.67)	1.56 (1.29−1.83)	9.33 (−15.77 to 34.51)	780.35 (648.66−912.26)	883.24 (736.26−1029.91)	13.18 (−11.98 to 38.44)	785.87 (652.43−919.53)	844.31 (700.34−988.14)	7.44 (−17.46 to 32.37)
Saint Vincent and the Grenadines	0.69 (0.57−0.80)	0.65 (0.54−0.75)	−6.02 (−28.66 to 16.70)	599.49 (501.48−697.85)	642.02 (539.39−744.70)	7.09 (−16.53 to 30.76)	605.65 (505.74−705.89)	652.37 (543.98−760.78)	7.71 (−16.55 to 32.06)
Serbia	42.49 (35.91−49.07)	35.90 (30.29−41.51)	−15.52 (−35.71 to 4.86)	481.23 (409.42−552.94)	484.80 (413.45−556.20)	0.74 (−20.19 to 21.69)	493.18 (416.73−569.55)	496.15 (418.62−573.73)	0.60 (−21.39 to 22.58)
South Africa	311.69 (262.69−360.73)	443.92 (374.98−513.05)	42.42 (15.72−69.67)	534.48 (449.07−619.96)	604.18 (512.20−696.42)	13.04 (−10.39 to 36.51)	526.66 (443.86−609.52)	601.87 (508.41−695.60)	14.28 (−9.31 to 37.95)
Suriname	3.68 (3.08−4.29)	4.46 (3.73−5.18)	21.01 (−4.28 to 46.52)	614.85 (513.98−715.66)	665.22 (560.11−770.20)	8.19 (−15.44 to 31.84)	614.78 (513.95−715.55)	672.02 (563.30−780.60)	9.31 (−14.72 to 33.38)
Thailand	284.04 (243.46−324.71)	282.54 (241.41−323.68)	−0.53 (−20.81 to 19.75)	416.75 (357.98−475.67)	497.10 (428.11−566.12)	19.28 (−2.31 to 41.03)	396.43 (339.79−453.19)	427.34 (365.12−489.56)	7.80 (−13.34 to 29.00)
Tonga	0.30 (0.26−0.35)	0.48 (0.41−0.55)	59.53 (32.90−86.55)	265.70 (226.48−304.97)	276.66 (237.10−316.17)	4.12 (−16.76 to 25.00)	267.98 (229.14−306.85)	278.96 (239.33−318.54)	4.09 (−16.54 to 24.69)
Turkey	310.92 (263.30−358.57)	381.27 (324.30−438.24)	22.63 (−1.03 to 46.49)	363.08 (307.44−418.77)	397.36 (337.88−456.86)	9.44 (−12.92 to 31.78)	358.53 (303.61−413.47)	352.35 (299.70−405.00)	−1.72 (−22.87 to 19.47)
Turkmenistan	18.06 (15.67−20.45)	29.34 (25.65−33.03)	62.44 (38.36−86.87)	316.56 (274.65−358.38)	433.87 (379.23−488.41)	37.06 (15.68−58.88)	333.94 (289.76−378.02)	419.75 (366.90−472.53)	25.70 (5.29−46.29)
Ukraine	133.67 (113.56−153.77)	100.34 (84.95−115.68)	−24.93 (−43.77 to −5.95)	313.66 (268.21−359.13)	316.68 (271.20−362.01)	0.96 (−19.43 to 21.33)	312.09 (265.14−359.03)	302.23 (255.89−348.44)	−3.16 (−24.23 to 17.86)
**L-MICs**									
Angola	166.96 (142.51−191.38)	619.29 (534.66−703.86)	270.93 (218.67−324.11)	578.56 (486.01−670.97)	1018.42 (873.79−1162.83)	76.03 (46.66−105.83)	493.36 (421.12−565.54)	1014.17 (875.57−1152.65)	105.56 (74.31−137.38)

Location	Number of cases			ASPR			All age prevalence		
	2023 (thousand)	2050 (thousand)	Percent change (%)	2023 (per 100,000)	2050 (per 100,000)	Percent change (%)	2023 (per 100,000)	2050 (per 100,000)	Percent change (%)
Bangladesh	624.80 (521.08−728.39)	692.39 (585.12−800.25)	10.82 (−13.03 to 34.65)	367.03 (306.09−427.89)	446.31 (376.49−516.49)	21.60 (−3.52 to 46.88)	379.78 (316.73−442.74)	407.41 (344.29−470.88)	7.28 (−16.14 to 30.68)
Benin	63.02 (54.06−71.98)	187.15 (162.64−211.59)	196.98 (156.23−238.54)	502.80 (425.70−579.90)	774.76 (670.09−879.14)	54.09 (28.74−80.00)	457.50 (392.48−522.56)	774.86 (673.39−876.05)	69.37 (43.31−95.82)
Bhutan	5.00 (4.19−5.81)	6.83 (5.76−7.89)	36.56 (10.21−63.49)	495.71 (413.87−577.50)	582.90 (494.23−671.51)	17.59 (−6.64 to 41.91)	485.33 (406.60−564.09)	574.32 (484.58−664.13)	18.34 (−6.13 to 42.94)
Bolivia (Plurinational State of)	50.48 (42.59−58.36)	110.52 (95.21−125.79)	118.94 (85.35−153.12)	410.87 (344.80−476.94)	591.66 (509.69−673.44)	44.00 (18.63−69.71)	390.27 (329.27−451.24)	590.26 (508.48−671.85)	51.24 (25.63−77.46)
Cabo Verde	3.55 (2.97−4.12)	5.25 (4.40−6.10)	48.09 (19.56−77.06)	636.07 (531.22−741.19)	724.24 (611.88−836.74)	13.86 (−10.25 to 37.96)	610.71 (512.04−709.61)	773.99 (648.63−899.53)	26.74 (0.93−53.01)
Cambodia	65.07 (55.69−74.46)	98.01 (85.21−110.81)	50.63 (26.70−75.09)	360.50 (307.96−413.14)	486.74 (423.61−549.94)	35.02 (12.43−57.82)	372.76 (319.04−426.59)	475.14 (413.11−537.23)	27.47 (5.57−49.49)
Cameroon	119.84 (103.85−135.84)	255.03 (222.16−287.96)	112.81 (82.72−143.52)	401.59 (343.47−459.79)	560.53 (486.54−634.66)	39.58 (16.44−63.14)	377.35 (326.99−427.74)	555.21 (483.64−626.89)	47.13 (24.32−70.41)
Comoros	4.06 (3.49−4.64)	6.68 (5.78−7.58)	64.46 (38.50−90.81)	476.25 (409.41−543.40)	701.17 (606.48−795.47)	47.23 (23.31−71.62)	519.38 (446.01−593.11)	686.38 (594.07−778.27)	32.15 (9.70−54.95)
Congo	30.12 (25.66−34.58)	62.90 (53.95−71.85)	108.80 (76.04−142.15)	599.24 (505.27−693.14)	954.39 (818.78−1090.16)	59.27 (32.21−86.83)	560.50 (477.52−643.44)	948.97 (814.02−1083.99)	69.31 (41.26−97.68)
Côte d'Ivoire	145.75 (124.78−166.80)	334.09 (287.25−380.99)	129.23 (94.51−164.62)	533.59 (451.72−615.92)	696.06 (596.05−796.24)	30.45 (6.40−54.74)	500.25 (428.28−572.49)	690.85 (593.99−787.82)	38.10 (14.43−62.33)
Djibouti	5.23 (4.54−5.92)	6.66 (5.81−7.51)	27.38 (6.54−48.45)	371.36 (322.58−420.19)	445.44 (387.96−502.96)	19.95 (−0.20 to 40.22)	416.23 (361.15−471.37)	426.42 (371.82−481.07)	2.45 (−16.12 to 21.01)
Egypt	441.84 (372.94−510.71)	615.49 (524.55−706.35)	39.30 (13.94−65.23)	373.67 (315.57−431.78)	412.25 (351.33−473.10)	10.32 (−12.11 to 32.81)	412.41 (348.10−476.70)	410.06 (349.47−470.58)	−0.57 (−21.88 to 20.85)
Eswatini	5.96 (5.04−6.88)	8.68 (7.31−10.04)	45.50 (18.34−73.07)	520.94 (435.21−606.68)	547.95 (460.51−635.16)	5.19 (−18.23 to 28.56)	481.43 (407.29−555.53)	531.91 (448.25−615.31)	10.48 (−12.56 to 33.63)
Ghana	161.14 (138.44−183.91)	323.21 (279.19−367.16)	100.58 (70.30−131.39)	492.25 (419.12−565.47)	664.90 (573.23−756.43)	35.08 (11.50−59.00)	473.43 (406.73−540.34)	662.91 (572.62−753.04)	40.02 (16.77−63.81)
Guinea	42.59 (37.31−47.87)	128.17 (113.43−142.93)	200.98 (164.72−237.97)	272.14 (238.64−305.72)	522.70 (462.17−583.25)	92.07 (67.02−117.54)	309.11 (270.83−347.50)	560.10 (495.65−624.58)	81.20 (57.14−105.47)
Haiti	62.06 (52.12−72.03)	80.23 (67.80−92.72)	29.28 (3.85−55.09)	482.95 (403.67−562.50)	499.19 (421.89−576.83)	3.36 (−19.56 to 26.26)	479.82 (402.99−556.91)	500.12 (422.63−577.96)	4.23 (−18.47 to 26.92)
Honduras	67.20 (56.42−77.98)	96.85 (81.41−112.29)	44.12 (16.58−72.24)	653.64 (546.15−761.23)	653.41 (549.63−757.21)	−0.03 (−22.85 to 22.82)	632.15 (530.75−733.58)	658.05 (553.16−762.99)	4.10 (−18.93 to 27.08)
India	6354.54 (5294.33−7417.25)	10081.60 (8380.99−11789.04)	58.65 (27.29−90.40)	437.01 (363.48−510.71)	658.83 (547.46−770.74)	50.76 (20.56−81.38)	435.35 (362.71−508.16)	628.17 (522.20−734.55)	44.29 (15.22−73.88)
Jordan	42.48 (35.83−49.13)	68.88 (58.00−79.71)	62.15 (32.43−92.20)	329.64 (278.14−381.11)	401.40 (337.92−464.48)	21.77 (−2.77 to 46.57)	354.19 (298.72−409.61)	385.57 (324.64−446.15)	8.86 (−14.25 to 32.05)

Location	Number of cases			ASPR			All age prevalence		
	2023 (thousand)	2050 (thousand)	Percent change (%)	2023 (per 100,000)	2050 (per 100,000)	Percent change (%)	2023 (per 100,000)	2050 (per 100,000)	Percent change (%)
Kenya	288.79 (248.42−329.22)	572.62 (491.17−654.06)	98.28 (67.23−129.96)	515.24 (440.27−590.26)	735.30 (629.94−840.65)	42.71 (18.06−67.87)	529.19 (455.22−603.29)	736.70 (631.92−841.48)	39.21 (15.37−63.54)
Kiribati	0.48 (0.41−0.55)	0.71 (0.59−0.82)	47.41 (19.65−75.56)	336.52 (284.48−388.57)	321.68 (269.78−373.58)	−4.41 (−26.15 to 17.32)	356.72 (302.40−411.06)	334.02 (280.67−387.42)	−6.36 (−27.66 to 14.89)
Kyrgyzstan	26.48 (22.92−30.04)	29.01 (25.05−32.97)	9.53 (−10.47 to 29.61)	359.22 (310.93−407.41)	312.04 (269.47−354.61)	−13.13 (−30.94 to 4.79)	380.11 (329.00−431.10)	308.81 (266.68−350.93)	−18.76 (−36.09 to −1.32)
Lao People’s Democratic Republic	27.11 (23.16−31.07)	50.97 (44.49−57.46)	88.00 (60.26−116.08)	348.56 (296.53−400.61)	569.60 (497.74−641.53)	63.41 (38.44−88.96)	356.98 (304.92−409.05)	555.47 (484.79−626.23)	55.60 (31.47−80.27)
Lebanon	27.16 (23.13−31.20)	33.56 (28.53−38.60)	23.55 (0.05−47.39)	283.27 (241.18−325.33)	312.82 (265.89−359.80)	10.43 (−11.72 to 32.64)	295.49 (251.60−339.33)	294.44 (250.33−338.60)	−0.35 (−21.33 to 20.61)
Lesotho	8.91 (7.50−10.32)	11.19 (9.35−13.04)	25.63 (−0.01 to 51.80)	454.52 (378.75−530.18)	462.56 (385.38−539.70)	1.77 (−21.94 to 25.43)	429.34 (361.23−497.39)	448.06 (374.21−521.80)	4.36 (−18.92 to 27.73)
Mauritania	27.63 (23.41−31.85)	64.37 (55.09−73.66)	133.01 (96.64−170.07)	540.04 (458.55−621.70)	832.79 (712.99−952.67)	54.21 (27.79−81.06)	606.14 (513.72−698.69)	860.09 (736.08−984.16)	41.90 (16.87−67.52)
Micronesia (Federated States of)	0.40 (0.33−0.46)	0.54 (0.46−0.62)	35.41 (10.04−61.30)	336.41 (283.14−389.78)	356.14 (302.14−410.04)	5.86 (−16.60 to 28.32)	352.05 (296.85−407.35)	358.91 (304.62−413.09)	1.95 (−19.99 to 23.84)
Morocco	126.70 (107.61−145.74)	137.12 (117.38−156.84)	8.22 (−13.38 to 29.79)	330.40 (280.64−380.05)	360.92 (308.74−413.06)	9.24 (−12.48 to 31.00)	337.11 (286.33−387.79)	323.07 (276.56−369.54)	−4.16 (−24.45 to 16.29)
Myanmar	174.80 (149.41−200.14)	287.52 (249.76−325.36)	64.48 (38.80−90.55)	311.57 (265.93−357.11)	476.02 (414.36−537.80)	52.78 (28.61−77.49)	313.66 (268.10−359.13)	452.78 (393.30−512.37)	44.35 (20.85−68.34)
Nepal	198.45 (165.07−231.86)	249.78 (210.16−289.38)	25.86 (0.00−51.93)	631.51 (523.89−739.17)	721.32 (608.86−833.81)	14.22 (−10.37 to 38.85)	624.04 (519.06−729.09)	728.46 (612.91−843.96)	16.73 (−8.14 to 41.72)
Nicaragua	34.23 (28.71−39.73)	42.55 (35.59−49.51)	24.32 (−1.29 to 50.38)	507.86 (424.80−590.75)	519.93 (437.23−602.60)	2.38 (−20.58 to 25.33)	496.34 (416.40−576.12)	537.09 (449.22−624.92)	8.21 (−15.57 to 32.10)
Nigeria	979.93 (857.32−1102.71)	3464.38 (3011.02−3916.35)	253.53 (206.00−301.67)	376.44 (328.47−424.43)	666.41 (579.03−753.59)	77.03 (50.93−103.55)	395.71 (346.20−445.29)	706.81 (614.32−799.03)	78.62 (52.47−105.26)
Pakistan	1226.54 (1029.11−1424.49)	1868.57 (1573.63−2163.54)	52.34 (23.80−81.32)	461.13 (386.95−535.50)	613.01 (516.08−709.94)	32.94 (6.89−59.52)	516.12 (433.04−599.41)	608.69 (512.62−704.78)	17.94 (−6.54 to 42.55)
Palestine	18.73 (15.95−21.52)	27.92 (23.79−32.05)	49.12 (22.90−75.73)	311.32 (265.37−357.53)	332.65 (283.42−381.85)	6.85 (−14.73 to 28.41)	339.82 (289.51−390.46)	333.92 (284.52−383.30)	−1.74 (−22.47 to 18.96)
Papua New Guinea	32.70 (27.62−37.81)	61.24 (52.08−70.40)	87.26 (55.67−119.40)	299.92 (252.29−347.69)	338.55 (287.27−389.78)	12.88 (−10.38 to 36.18)	308.04 (260.20−356.10)	351.14 (298.57−403.64)	13.99 (−8.99 to 37.04)
Philippines	440.89 (373.87−507.67)	691.38 (586.79−796.43)	56.82 (28.86−85.16)	371.86 (314.78−428.78)	452.38 (383.90−521.15)	21.65 (−2.11 to 45.67)	386.64 (327.87−445.20)	453.06 (384.52−521.90)	17.18 (−5.99 to 40.54)
Samoa	0.72 (0.61−0.83)	1.43 (1.22−1.64)	97.77 (65.52−130.59)	326.84 (275.95−377.66)	376.44 (320.33−432.59)	15.18 (−7.88 to 38.33)	324.07 (274.89−373.17)	384.25 (328.07−440.50)	18.57 (−4.31 to 41.62)
Sao Tome and Principe	1.33 (1.13−1.53)	2.22 (1.89−2.56)	67.12 (37.76−96.73)	638.66 (538.24−739.31)	813.43 (689.82−937.22)	27.36 (2.62−52.38)	611.85 (519.21−704.78)	813.16 (689.54−937.02)	32.90 (8.10−58.28)

Location	Number of cases			ASPR			All age prevalence		
	2023 (thousand)	2050 (thousand)	Percent change (%)	2023 (per 100,000)	2050 (per 100,000)	Percent change (%)	2023 (per 100,000)	2050 (per 100,000)	Percent change (%)
Senegal	81.11 (70.04−92.19)	261.28 (228.08−294.44)	222.13 (179.54−265.64)	418.49 (362.11−474.95)	884.10 (772.07−996.05)	111.26 (81.69−141.34)	473.60 (408.96−538.30)	931.71 (813.34−1049.98)	96.73 (68.63−125.30)
Solomon Islands	2.48 (2.12−2.85)	3.50 (3.00−4.00)	41.00 (16.52−66.03)	325.11 (275.85−374.27)	322.25 (275.74−368.60)	−0.88 (−21.60 to 19.93)	345.45 (294.27−396.50)	335.50 (287.61−383.22)	−2.88 (−23.07 to 17.38)
Sri Lanka	113.93 (95.63−132.20)	107.20 (89.93−124.50)	−5.91 (−27.89 to 16.17)	523.53 (439.64−607.30)	554.76 (466.95−642.86)	5.97 (−17.15 to 29.13)	516.07 (433.18−598.84)	511.94 (429.47−594.52)	−0.80 (−23.35 to 21.75)
Tajikistan	42.77 (36.92−48.63)	50.88 (43.58−58.17)	18.97 (−2.70 to 40.93)	373.73 (322.67−424.83)	309.90 (265.31−354.44)	−17.08 (−35.08 to 1.08)	407.83 (352.02−463.71)	316.80 (271.36−362.20)	−22.32 (−39.90 to −4.63)
Timor-Leste	5.61 (4.79−6.43)	10.33 (8.95−11.70)	84.15 (55.89−112.63)	366.45 (311.88−421.00)	460.39 (398.88−521.92)	25.64 (3.35−48.02)	388.58 (331.68−445.44)	467.20 (405.15−529.26)	20.23 (−1.33 to 41.87)
Tunisia	31.75 (27.12−36.35)	35.19 (30.15−40.25)	10.86 (−10.60 to 32.38)	265.14 (226.50−303.62)	294.18 (251.87−336.58)	10.95 (−10.54 to 32.53)	262.72 (224.45−300.85)	258.98 (221.85−296.21)	−1.43 (−21.67 to 18.81)
United Republic of Tanzania	300.19 (259.35−341.10)	1345.08 (1174.32−1516.09)	348.07 (289.78−407.02)	429.12 (369.43−488.91)	1043.65 (911.29−1176.22)	143.20 (109.83−177.19)	463.67 (400.59−526.85)	1098.95 (959.44−1238.67)	137.01 (104.45−170.18)
Uzbekistan	116.06 (100.92−131.18)	144.45 (126.25−162.68)	24.46 (4.24−44.88)	311.83 (271.13−352.47)	347.42 (303.60−391.33)	11.41 (−7.71 to 30.57)	332.41 (289.04−375.72)	337.61 (295.09−380.23)	1.57 (−16.67 to 19.76)
Vanuatu	1.11 (0.94−1.28)	1.86 (1.58−2.13)	67.34 (38.49−96.54)	323.27 (273.42−373.08)	344.94 (293.08−396.84)	6.70 (−15.50 to 28.92)	339.77 (288.36−391.22)	360.03 (306.39−413.70)	5.96 (−15.86 to 27.82)
Viet Nam	296.29 (252.81−339.73)	298.84 (254.32−343.41)	0.86 (−20.08 to 21.75)	295.01 (251.62−338.34)	299.49 (256.04−343.02)	1.52 (−19.21 to 22.22)	293.59 (250.50−336.63)	280.62 (238.82−322.48)	−4.42 (−24.82 to 15.99)
Zambia	97.09 (83.50−110.67)	342.11 (298.28−385.85)	252.37 (205.59−299.93)	457.11 (389.42−524.79)	915.94 (796.92−1034.71)	100.38 (70.83−130.33)	470.14 (404.33−535.93)	952.02 (830.06−1073.73)	102.50 (73.42−132.13)
Zimbabwe	89.66 (75.79−103.56)	145.70 (122.90−168.46)	62.51 (33.06−92.32)	562.70 (470.02−655.62)	560.98 (469.72−652.10)	−0.30 (−23.37 to 22.71)	528.79 (447.02−610.79)	537.16 (453.10−621.07)	1.58 (−20.53 to 23.65)
**LICs**									
Afghanistan	167.40 (142.16−192.58)	338.85 (288.33−389.29)	102.42 (69.14−136.27)	345.89 (294.15−397.46)	351.54 (299.71−403.31)	1.63 (−19.42 to 22.67)	418.58 (355.45−481.53)	404.17 (343.91−464.32)	−3.44 (−24.20 to 17.36)
Burkina Faso	72.56 (63.65−81.48)	269.31 (239.05−299.59)	271.17 (228.04−315.06)	276.00 (240.58−311.46)	514.42 (455.20−573.64)	86.39 (61.60−111.38)	286.69 (251.48−321.94)	542.07 (481.15−603.01)	89.08 (64.85−113.64)
Burundi	58.22 (50.50−65.94)	121.92 (105.62−138.23)	109.40 (78.87−140.59)	441.40 (379.04−503.74)	458.41 (394.29−522.60)	3.86 (−16.35 to 24.01)	445.15 (386.12−504.16)	461.91 (400.16−523.69)	3.77 (−15.36 to 22.93)
Central African Republic	19.22 (16.44−22.01)	21.65 (18.24−25.04)	12.61 (−10.04 to 35.49)	446.63 (376.31−517.10)	438.83 (369.05−508.49)	−1.75 (−23.85 to 20.33)	398.01 (340.47−455.70)	428.33 (361.01−495.49)	7.62 (−14.45 to 29.88)
Chad	53.74 (47.09−60.39)	223.39 (197.32−249.50)	315.70 (265.85−366.13)	267.50 (232.83−302.19)	417.45 (367.29−467.65)	56.05 (33.65−78.85)	281.31 (246.51−316.13)	453.77 (400.82−506.82)	61.31 (39.09−83.88)
Democratic People's Republic of Korea	70.89 (61.07−80.71)	60.07 (51.65−68.47)	−15.26 (−33.34 to 3.00)	277.47 (239.25−315.71)	282.50 (244.12−320.82)	1.82 (−17.62 to 21.23)	272.91 (235.12−310.72)	255.21 (219.42−290.90)	−6.49 (−25.47 to 12.57)

Location	Number of cases			ASPR			All age prevalence		
	**2023 (thousand)**	**2050 (thousand)**	**Percent change (%)**	**2023 (per 100,000)**	**2050 (per 100,000)**	**Percent change (%)**	**2023 (per 100,000)**	**2050 (per 100,000)**	**Percent change (%)**
Democratic Republic of the Congo	422.77 (365.78−479.73)	618.80 (526.17−711.44)	46.37 (20.84−72.19)	476.61 (406.94−546.16)	405.70 (341.80−469.63)	−14.88 (−34.59 to 4.98)	441.42 (381.92−500.90)	366.20 (311.38−421.03)	−17.04 (−35.28 to 1.30)
Eritrea	32.75 (28.12−37.37)	77.71 (67.53−87.87)	137.27 (103.67−171.51)	468.74 (400.28−537.12)	870.15 (755.10−984.92)	85.64 (57.35−114.17)	499.56 (428.95−570.04)	884.16 (768.35−999.69)	76.99 (50.14−104.16)
Ethiopia	562.91 (482.85−642.94)	3822.55 (3285.86−4358.91)	579.07 (482.15−675.31)	440.05 (375.42−504.67)	1800.59 (1547.54−2053.48)	309.18 (250.16−368.86)	459.45 (394.11−524.77)	1862.25 (1600.79−2123.55)	305.32 (246.95−364.30)
Gambia	13.44 (11.47−15.39)	24.12 (20.76−27.47)	79.47 (51.00−108.40)	479.33 (410.34−548.03)	624.71 (537.87−711.43)	30.33 (7.48−53.56)	549.08 (468.83−628.83)	656.19 (564.97−747.32)	19.51 (−2.46 to 41.59)
Guinea-Bissau	9.25 (7.98−10.50)	26.95 (23.66−30.25)	191.54 (153.97−229.90)	391.92 (337.15−446.58)	766.55 (671.72−861.46)	95.59 (68.01−123.52)	428.17 (369.65−486.46)	807.30 (708.58−906.11)	88.55 (62.05−115.38)
Liberia	28.11 (24.09−32.14)	37.68 (31.55−43.85)	34.07 (8.30−60.23)	546.26 (463.14−629.44)	489.55 (408.56−570.97)	−10.38 (−31.61 to 10.82)	517.28 (443.31−591.39)	467.15 (391.15−543.53)	−9.69 (−30.17 to 10.73)
Madagascar	152.01 (130.64−173.41)	377.00 (325.01−429.19)	148.01 (111.64−185.18)	463.05 (395.52−530.72)	579.64 (498.19−661.38)	25.18 (2.51−48.09)	484.63 (416.52−552.85)	609.57 (525.50−693.95)	25.78 (3.62−48.21)
Malawi	96.76 (83.67−109.88)	397.52 (349.97−445.05)	310.84 (260.27−362.16)	452.92 (388.79−517.22)	1160.63 (1020.35−1300.86)	156.25 (122.73−190.49)	477.88 (413.25−542.69)	1203.79 (1059.81−1347.71)	151.90 (119.36−185.05)
Mali	77.87 (68.09−87.65)	398.88 (353.16−444.60)	412.24 (352.18−472.53)	267.33 (234.65−300.00)	684.00 (606.47−761.55)	155.87 (124.84−187.47)	316.15 (276.45−355.84)	760.65 (673.47−847.83)	140.60 (110.75−171.03)
Mozambique	164.79 (142.00−187.50)	370.43 (322.48−418.31)	124.78 (93.07−157.18)	472.90 (402.84−542.82)	724.75 (628.17−821.11)	53.26 (28.55−78.56)	477.94 (411.85−543.81)	730.89 (636.29−825.37)	52.93 (29.22−77.13)
Niger	85.61 (74.99−96.20)	340.77 (300.42−381.20)	298.03 (249.59−347.13)	265.39 (233.53−297.21)	391.72 (346.25−437.24)	47.60 (27.03−68.60)	314.92 (275.83−353.86)	452.59 (399.00−506.28)	43.71 (23.07−64.86)
Rwanda	63.06 (54.67−71.47)	321.96 (284.38−359.49)	410.53 (349.64−471.91)	426.40 (366.76−486.16)	1240.47 (1094.73−1385.96)	190.92 (154.66−227.95)	429.16 (372.04−486.38)	1265.47 (1117.76−1412.96)	194.87 (158.55−231.89)
Sierra Leone	26.51 (23.34−29.68)	55.58 (49.22−61.94)	109.63 (83.18−136.56)	264.03 (232.19−295.84)	410.82 (363.20−458.45)	55.59 (34.20−77.30)	294.45 (259.21−329.65)	420.50 (372.40−468.62)	42.81 (22.95−63.12)
Somalia	72.38 (63.24−81.50)	248.62 (219.54−277.68)	243.48 (201.78−285.87)	298.60 (261.42−335.72)	563.04 (497.67−628.41)	88.56 (63.69−113.74)	359.83 (314.37−405.17)	645.23 (569.77−720.64)	79.31 (55.09−103.78)
South Sudan	36.08 (31.57−40.58)	133.14 (117.79−148.47)	269.05 (225.12−313.76)	263.71 (230.45−296.92)	436.68 (386.09−487.22)	65.59 (42.97−88.57)	297.12 (260.00−334.20)	482.23 (426.65−537.75)	62.30 (40.13−84.80)
Sudan	144.33 (123.72−164.99)	278.74 (242.66−314.78)	93.12 (64.71−121.94)	273.19 (234.43−312.02)	390.26 (339.71−440.74)	42.85 (19.91−66.26)	310.42 (266.08−354.85)	395.73 (344.50−446.90)	27.48 (5.79−49.30)
Syrian Arab Republic	57.76 (48.93−66.59)	52.51 (44.57−60.44)	−9.08 (−29.46 to 11.50)	298.85 (253.10−344.60)	292.67 (248.29−336.97)	−2.07 (−23.34 to 19.23)	314.69 (266.59−362.81)	263.15 (223.36−302.86)	−16.38 (−36.12 to 3.49)
Togo	43.54 (37.24−49.84)	74.39 (63.77−85.05)	70.85 (42.75−99.27)	549.22 (464.95−633.57)	640.31 (547.92−733.02)	16.59 (−6.10 to 39.35)	512.14 (438.04−586.23)	633.77 (543.30−724.57)	23.75 (1.09−46.64)
Uganda	235.59 (204.36−266.96)	1005.01 (883.35−1126.49)	326.58 (273.59−380.19)	476.52 (409.95−543.25)	1091.09 (957.27−1224.74)	128.97 (98.01−160.48)	495.61 (429.90−561.58)	1134.46 (997.12−1271.59)	128.90 (98.67−159.79)

Location	Number of cases			ASPR			All age prevalence		
	**2023 (thousand)**	**2050 (thousand)**	**Percent change (%)**	**2023 (per 100,000)**	**2050 (per 100,000)**	**Percent change (%)**	**2023 (per 100,000)**	**2050 (per 100,000)**	**Percent change (%)**
Yemen	127.16 (107.81−146.53)	188.01 (161.12−214.87)	47.86 (22.32−73.98)	308.40 (261.86−354.98)	350.59 (300.47−400.69)	13.68 (−8.42 to 35.81)	359.15 (304.50−413.88)	358.75 (307.45−410.00)	−0.11 (−20.91 to 20.76)

U-MICs. Upper-middle-income countries; L-MICs. Low-middle-income countries; LICs. Low-income countries

Table S5 Projected age-specific prevalence of idiopathic epilepsy and secondary epilepsy in low- and middle-income countries (LMICs) in 2023 and 2050, along with percent changes from 2023 to 2050, both sexes (95% UI)

Age group	Idiopathic epilepsy			Secondary epilepsy								
				Neglected tropical diseases and malaria			Other infectious diseases			Neonatal disorders		
	2023 (per 100,000)	2050 (per 100,000)	Percent change (%)	2023 (per 100,000)	2050 (per 100,000)	Percent change (%)	2023 (per 100,000)	2050 (per 100,000)	Percent change (%)	2023 (per 100,000)	2050 (per 100,000)	Percent change (%)
<5	283.65<br>(204.50−362.73)	294.18<br>(211.85−376.59)	3.71<br>(−30.90 to 55.42)	4.17<br>(3.95−4.39)	5.95<br>(5.65−6.25)	42.68<br>(32.56−53.32)	8.05<br>(6.82−9.27)	8.85<br>(7.52−10.17)	9.94<br>(−11.27 to 36.15)	432.25<br>(367.01−497.85)	673.01<br>(573.98−772.13)	55.70<br>(25.74−92.10)
5−9	320.74<br>(230.64−411.00)	341.26<br>(245.54−437.19)	6.40<br>(−29.86 to 59.94)	10.87<br>(10.28−11.46)	16.87<br>(15.99−17.75)	55.19<br>(43.88−67.28)	7.92<br>(6.61−9.23)	8.67<br>(7.29−10.05)	9.53<br>(−12.82 to 38.45)	409.45<br>(348.66−470.15)	671.58<br>(574.87−768.65)	64.02<br>(33.60−102.75)
10−14	349.65<br>(251.98−447.21)	376.18<br>(270.76−481.65)	7.59<br>(−29.21 to 64.71)	16.66<br>(15.56−17.76)	25.78<br>(24.27−27.30)	54.75<br>(41.49−69.12)	5.91<br>(4.88−6.93)	6.11<br>(5.09−7.13)	3.44<br>(−19.33 to 32.01)	410.05<br>(348.68−471.12)	671.70<br>(574.87−768.65)	63.81<br>(33.42−102.65)
15−19	349.67<br>(251.56−447.49)	381.41<br>(275.01−487.87)	9.08<br>(−27.39 to 64.36)	24.21<br>(21.66−26.76)	34.79<br>(31.88−37.69)	43.71<br>(25.63−64.78)	4.53<br>(3.72−5.33)	4.57<br>(3.78−5.36)	1.08<br>(−21.38 to 29.97)	393.88<br>(334.68−453.22)	648.34<br>(555.23−741.46)	64.61<br>(33.56−104.12)
20−24	332.61<br>(239.36−426.31)	364.94<br>(263.14−466.96)	9.72<br>(−26.97 to 65.34)	35.85<br>(30.57−41.12)	46.36<br>(40.77−51.94)	29.31<br>(7.17−57.06)	3.42<br>(2.79−4.04)	3.51<br>(2.89−4.12)	2.61<br>(−20.60 to 33.47)	371.97<br>(317.26−426.48)	615.09<br>(526.40−703.70)	65.36<br>(33.97−104.15)
25−29	305.41<br>(220.19−390.34)	332.53<br>(239.92−425.17)	8.88<br>(−26.51 to 63.82)	47.84<br>(39.64−56.03)	56.97<br>(48.70−65.22)	19.07<br>(−4.49 to 49.56)	2.67<br>(2.17−3.16)	2.81<br>(2.30−3.31)	5.24<br>(−18.89 to 36.77)	349.39<br>(297.36−401.74)	582.43<br>(499.15−665.70)	66.70<br>(35.46−105.82)
30−34	272.96<br>(195.68−350.40)	296.19<br>(213.07−379.35)	8.51<br>(−27.97 to 64.34)	53.05<br>(43.32−62.79)	60.09<br>(50.65−69.54)	13.28<br>(−10.53 to 45.73)	2.18<br>(1.78−2.59)	2.38<br>(1.96−2.81)	9.12<br>(−15.85 to 41.57)	321.53<br>(274.53−368.74)	541.08<br>(463.13−619.08)	68.29<br>(37.53−108.17)
35−39	250.00<br>(180.00−320.08)	270.89<br>(195.12−346.44)	8.36<br>(−27.68 to 65.24)	59.43<br>(48.13−70.72)	64.13<br>(53.63−74.60)	7.92<br>(−15.51 to 39.90)	1.99<br>(1.62−2.36)	2.28<br>(1.87−2.68)	14.23<br>(−11.76 to 48.32)	298.29<br>(254.69−342.04)	503.13<br>(431.29−574.74)	68.67<br>(37.57−108.12)
40−44	236.18<br>(170.34−301.85)	252.27<br>(181.66−322.81)	6.81<br>(−28.80 to 60.40)	66.58<br>(53.73−79.47)	68.96<br>(57.05−80.88)	3.58<br>(−20.04 to 34.89)	2.02<br>(1.64−2.39)	2.35<br>(1.93−2.77)	16.57<br>(−9.72 to 50.83)	277.60<br>(237.68−317.66)	463.73<br>(397.45−530.57)	67.05<br>(36.18−105.04)
45−49	222.69<br>(160.76−284.57)	239.81<br>(172.86−306.61)	7.69<br>(−28.13 to 62.50)	71.25<br>(56.99−85.49)	71.61<br>(58.60−84.65)	0.49<br>(−22.87 to 33.43)	2.14<br>(1.74−2.53)	2.53<br>(2.08−2.98)	18.25<br>(−8.52 to 53.00)	256.22<br>(219.19−293.38)	433.47<br>(372.56−494.34)	69.18<br>(38.52−106.56)
50−54	217.41<br>(156.71−278.30)	238.75<br>(171.75−305.95)	9.81<br>(−27.42 to 65.66)	78.68<br>(62.61−94.77)	77.68<br>(63.08−92.28)	−1.27<br>(−25.19 to 31.11)	2.42<br>(1.96−2.88)	2.89<br>(2.37−3.41)	19.38<br>(−8.37 to 55.41)	235.16<br>(201.52−268.84)	405.67<br>(348.53−463.08)	72.51<br>(41.38−113.30)
55−59	239.17<br>(172.26−306.27)	262.14<br>(188.68−335.75)	9.60<br>(−27.71 to 64.90)	101.88<br>(80.84−122.99)	97.85<br>(78.52−117.11)	−3.95<br>(−28.19 to 28.40)	3.24<br>(2.62−3.86)	3.71<br>(3.03−4.39)	14.53<br>(−12.22 to 50.09)	221.59<br>(190.55−252.69)	383.75<br>(329.76−437.53)	73.18<br>(42.15−111.96)
60−64	293.25<br>(211.10−375.00)	309.08<br>(222.66−395.14)	5.40<br>(−30.15 to 58.09)	141.05<br>(111.78−170.59)	129.49<br>(103.68−155.56)	−8.19<br>(−31.99 to 24.00)	5.17<br>(4.18−6.17)	5.20<br>(4.23−6.17)	0.54<br>(−23.22 to 33.04)	200.96<br>(172.85−229.23)	343.07<br>(295.85−390.41)	70.71<br>(39.98−109.14)
65−69	337.41<br>(243.01−431.01)	365.57<br>(262.89−468.90)	8.34<br>(−27.66 to 61.48)	176.94<br>(140.17−213.83)	164.60<br>(131.18−197.75)	−6.98<br>(−30.60 to 25.36)	7.33<br>(5.91−8.77)	7.50<br>(6.09−8.90)	2.23<br>(−22.30 to 34.58)	136.56<br>(117.62−155.36)	243.81<br>(210.14−277.70)	78.53<br>(46.97−117.98)
70−74	378.08<br>(272.10−484.34)	414.18<br>(298.17−529.71)	9.55<br>(−27.78 to 65.13)	221.72<br>(176.38−267.07)	204.64<br>(163.10−246.57)	−7.70<br>(−31.29 to 24.27)	9.62<br>(7.74−11.51)	9.53<br>(7.72−11.33)	−0.99<br>(−24.81 to 30.56)	67.30<br>(57.83−76.78)	121.09<br>(104.37−137.86)	79.92<br>(47.14−119.13)
75−79	431.98<br>(310.62−553.47)	454.80<br>(328.04−581.33)	5.28<br>(−29.93 to 61.22)	273.91<br>(217.95−329.35)	240.16<br>(191.49−288.93)	−12.32<br>(−34.44 to 16.82)	12.04<br>(9.71−14.40)	10.60<br>(8.60−12.61)	−11.97<br>(−33.35 to 15.51)	25.37<br>(21.81−28.94)	45.37<br>(39.09−51.65)	78.85<br>(46.48−118.03)
80−84	490.92<br>(353.08−628.70)	505.70<br>(364.88−646.42)	3.01<br>(−31.45 to 55.32)	315.65<br>(250.80−380.69)	266.81<br>(212.38−321.19)	−15.47<br>(−36.69 to 14.11)	14.27<br>(11.55−17.01)	11.53<br>(9.38−13.70)	−19.17<br>(−38.72 to 5.68)	7.52<br>(6.49−8.55)	13.73<br>(11.89−15.58)	82.66<br>(50.31−122.16)

Age group	Idiopathic epilepsy			Secondary epilepsy								
				Neglected tropical diseases and malaria			Other infectious diseases			Neonatal disorders		
	2023 (per 100,000)	2050 (per 100,000)	Percent change (%)	2023 (per 100,000)	2050 (per 100,000)	Percent change (%)	2023 (per 100,000)	2050 (per 100,000)	Percent change (%)	2023 (per 100,000)	2050 (per 100,000)	Percent change (%)
85−89	559.84 (401.75−717.92)	585.00 (420.66−749.91)	4.49 (−30.40 to 56.93)	344.10 (274.26−414.07)	297.36 (237.18−357.36)	−13.58 (−34.93 to 15.56)	16.54 (13.50−19.60)	13.84 (11.32−16.36)	−16.35 (−35.58 to 8.07)	1.80 (1.57−2.03)	3.41 (2.98−3.83)	89.20 (57.69−126.25)
90−94	669.69 (482.98−858.24)	711.60 (512.94−911.88)	6.26 (−28.96 to 62.28)	401.51 (319.59−483.47)	363.93 (289.29−438.88)	−9.36 (−32.78 to 21.13)	18.87 (15.55−22.18)	17.06 (14.01−20.13)	−9.59 (−30.06 to 16.05)	0.35 (0.31−0.40)	0.73 (0.64−0.82)	106.55 (74.41−144.93)
≥95	906.67 (654.28−1158.97)	951.98 (686.14−1219.23)	5.00 (−31.08 to 58.24)	560.68 (448.81−672.74)	504.93 (404.31−606.58)	−9.94 (−32.30 to 19.10)	20.12 (16.64−23.58)	17.69 (14.63−20.74)	−12.07 (−31.32 to 12.91)	0.06 (0.05−0.07)	0.16 (0.14−0.18)	167.85 (129.86−214.69)

Table S6 Projected number of cases of epilepsy in low- and middle-income countries (LMICs), by sex, 2050 (95% UI)

Cause	Female (thousand)	Male (thousand)
Epilepsy (idiopathic and secondary)	34,348.70 (27,518.74−41,187.26)	37,691.84 (30,340.75−45,044.87)
Idiopathic epilepsy	12,654.80 (9113.98−16,203.26)	13,876.50 (9996.04−17,752.58)
Secondary epilepsy	21,693.90 (18,404.76−24,984.00)	23815.34 (20,344.70−27,292.29)
Malaria	577.35 (547.83−606.91)	548.60 (521.92−575.31)
Cysticercosis	2934.05 (2348.03−3520.76)	2437.52 (1950.88−2924.70)
Cystic echinococcosis	9.68 (6.70−12.66)	8.75 (6.05−11.44)
Food-borne trematodiases	43.11 (23.74−62.37)	66.27 (36.52−95.88)
Meningitis	132.71 (114.08−151.34)	135.00 (116.15−153.86)
Encephalitis	75.93 (57.80−94.02)	70.09 (53.20−86.99)
Tetanus	1.39 (1.19−1.58)	1.56 (1.34−1.77)
Neonatal preterm birth	9373.96 (7678.90−11069.68)	9745.45 (7992.72−11504.21)
Neonatal encephalopathy due to birth asphyxia and trauma	4485.91 (4037.32−4934.50)	6230.91 (5607.82−6854.00)
Neonatal sepsis and other neonatal infections	2450.78 (2205.85−2695.71)	3130.49 (2817.66−3443.31)
Hemolytic disease and other neonatal jaundice	1609.01 (1383.31−1834.46)	1440.72 (1240.45−1640.82)

Table S7 Model performance in predicting epilepsy prevalence compared with GBD 2023 reference data from 2011 to 2023

Cause	Female				Male			
	**RMSEs**	**MAE**	**MAPE**	R^2	**RMSEs**	**MAE**	**MAPE**	R^2
Idiopathic epilepsy	<0.0001	<0.0001	<0.0001%	>0.9999	<0.0001	<0.0001	<0.0001%	>0.9999
Secondary epilepsy								
Malaria	<0.0001	<0.0001	0.0140%	>0.9999	0.0001	<0.0001	0.1118%	>0.9999
Cysticercosis	0.0001	<0.0001	0.0249%	>0.9999	0.0001	<0.0001	0.0387%	>0.9999
Cystic echinococcosis	<0.0001	<0.0001	0.0530%	>0.9999	<0.0001	<0.0001	0.0631%	>0.9999
Food-borne trematodiases	<0.0001	<0.0001	0.4330%	>0.9999	<0.0001	<0.0001	0.0070%	>0.9999
Meningitis	0.0001	<0.0001	0.0191%	>0.9999	0.0001	<0.0001	0.0051%	>0.9999
Encephalitis	0.0001	<0.0001	0.0146%	>0.9999	<0.0001	<0.0001	0.0118%	>0.9999
Tetanus	<0.0001	<0.0001	0.0888%	>0.9999	<0.0001	<0.0001	1.1828%	>0.9999
Neonatal preterm birth	<0.0001	<0.0001	0.0657%	>0.9999	0.0001	<0.0001	0.1003%	>0.9999
Neonatal encephalopathy due to birth asphyxia and trauma	0.0001	<0.0001	0.2015%	>0.9999	<0.0001	<0.0001	0.0206%	>0.9999
Neonatal sepsis and other neonatal infections	<0.0001	<0.0001	0.1769%	>0.9999	<0.0001	<0.0001	0.2295%	>0.9999
Hemolytic disease and other neonatal jaundice	0.0001	<0.0001	0.1754%	>0.9999	<0.0001	<0.0001	2.3090%	>0.9999

RMSE. Root mean squared error; MAE. Mean absolute error; MAPE. Mean absolute percentage error; R^2 . Coefficient of determination

Table S8 Root mean squared errors (RMSEs) between predicted epilepsy prevalence and GBD 2023 reference data of the six original models and the proposed model

Training interval	Validation interval	Model 1	Model 2	Model 3	Model 4	Model 5	Model 6	Proposed model
1990–2006	2007–2023	0.0240	0.0109	0.0116	0.0108	0.0109	0.0107	0.0002
1990–2010	2011–2023	0.0240	0.0084	0.0096	0.0084	0.0083	0.0082	<0.0001
1990–2014	2015–2023	0.0233	0.0068	0.0083	0.0069	0.0068	0.0065	<0.0001

Models 1–6 correspond to models 1–6 described in the **Additional file 1: Methods**. The proposed model refers to the hybrid DNN-Transformer model integrating predictions from Models 1–6.

Table S9 Contributions of population growth, aging, and prevalence change to projected percentage changes in the number of cases with epilepsy (idiopathic and secondary), idiopathic epilepsy, and secondary epilepsy from 2023 to 2050 in low- and middle-income countries (LMICs), by income level and for the top 10 countries, both sexes

Location	Population growth (%)	Aging (%)	Prevalence change (%)	Total percentage change (%)
Epilepsy (idiopathic and secondary)				
LMICs	21.40	–0.51	37.79	58.68
U-MICs	1.59	2.23	16.25	20.07
L-MICs	27.00	–2.70	39.53	63.83
LICs	76.22	–3.55	95.79	168.46
Ethiopia	67.54	–2.77	319.70	384.47
Mali	112.90	–6.06	181.48	288.32
Rwanda	73.13	–0.02	174.65	247.77
Chad	157.71	–2.13	88.41	243.99
Niger	176.96	–3.38	68.69	242.27
United Republic of Tanzania	89.05	–2.60	133.45	219.90
Uganda	86.36	–3.88	121.58	204.06
Malawi	63.09	–5.62	135.41	192.89
South Sudan	127.38	–2.07	62.05	187.36
Nigeria	97.92	–0.38	88.30	185.85
Idiopathic epilepsy				
LMICs	21.40	2.78	9.39	33.56
U-MICs	1.59	6.86	8.56	17.01
L-MICs	27.00	–0.41	11.44	38.04
LICs	76.22	–1.79	1.69	76.12
Niger	176.96	0.50	–0.60	176.86
Chad	157.71	0.84	18.30	176.85
Mali	112.90	–1.02	20.29	132.18
Nigeria	97.92	3.27	12.51	113.70
South Sudan	127.38	1.16	–17.63	110.91
Afghanistan	109.64	–5.71	0.23	104.15
Burkina Faso	96.30	2.66	3.53	102.49
Burundi	101.80	1.51	–1.82	101.48
Angola	80.45	6.98	13.87	101.29

Location	Population growth (%)	Aging (%)	Prevalence change (%)	Total percentage change (%)
Equatorial Guinea	61.91	2.28	30.29	94.48
Secondary epilepsy				
LMICs	21.40	–3.06	59.89	78.23
U-MICs	1.59	–1.91	23.13	22.81
L-MICs	27.00	–4.28	58.97	81.69
LICs	76.22	–4.94	170.23	241.50
Ethiopia	67.54	–5.80	517.33	579.07
Mali	112.90	–10.06	309.39	412.24
Rwanda	73.13	–1.05	338.44	410.53
United Republic of Tanzania	89.05	–6.70	265.72	348.07
Uganda	86.36	–5.87	246.09	326.58
Chad	157.71	–5.31	163.30	315.70
Malawi	63.09	–7.77	255.51	310.84
Niger	176.96	–6.68	127.75	298.03
Burkina Faso	96.30	–5.17	180.04	271.17
Angola	80.45	13.20	177.28	270.93

U-MICs. Upper-middle-income countries; L-MICs. Low-middle-income countries; LICs. Low-income countries

Figure legends

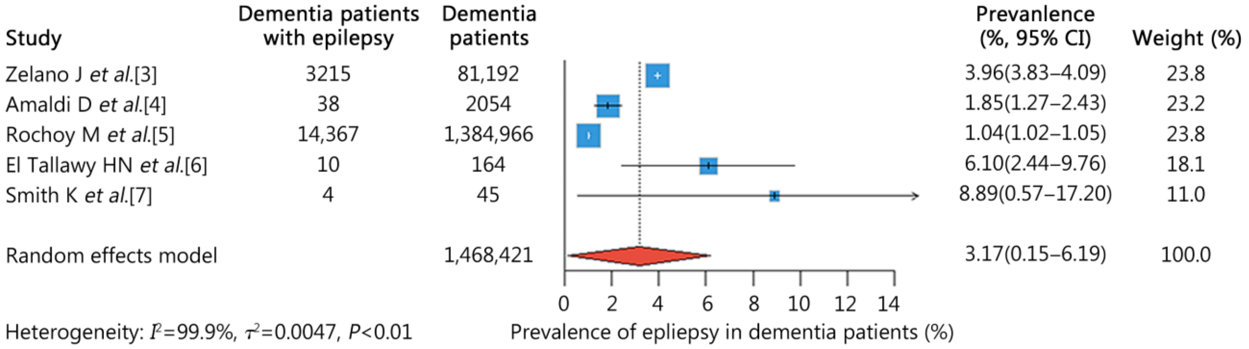

Fig. S1 Forest plot of the prevalence of epilepsy in dementia patients

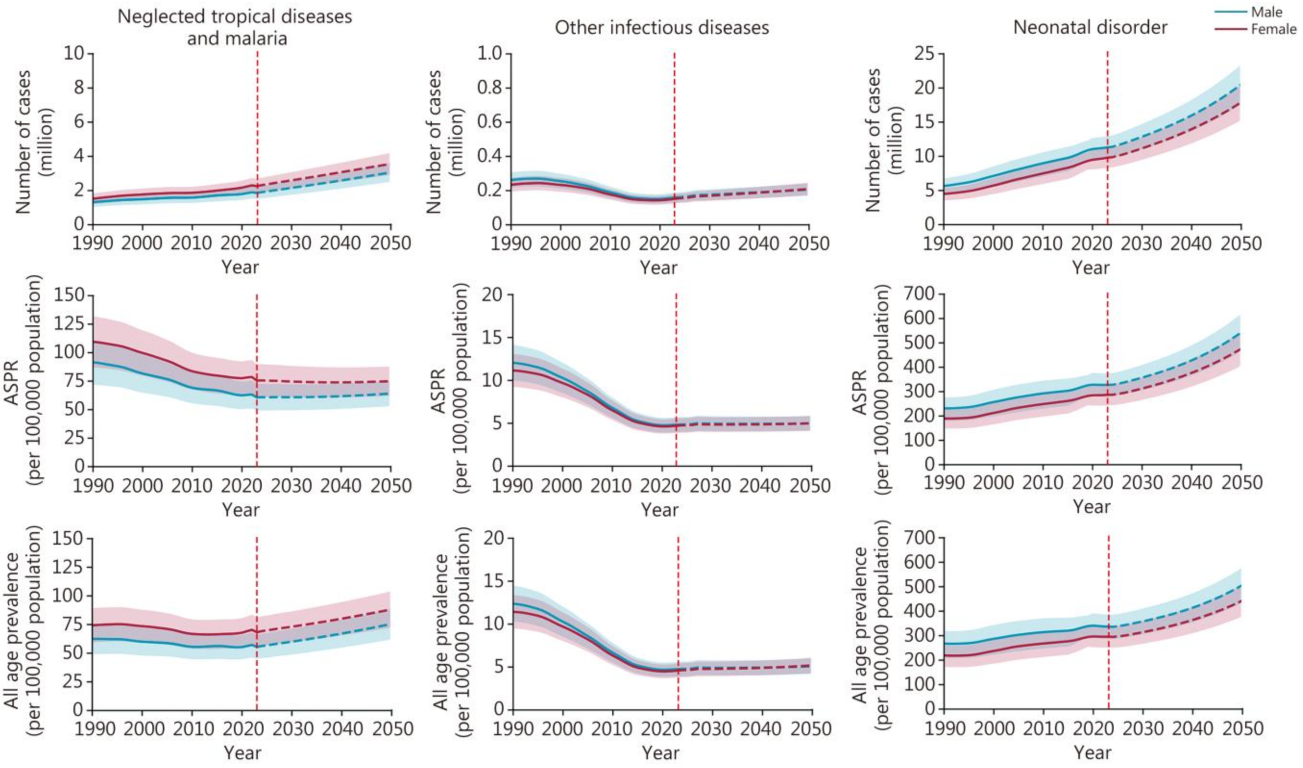

Fig. S2 Projected trends in the number of cases, ASPR, and all-age prevalence of secondary epilepsy caused by neglected tropical diseases and malaria, other infectious diseases, and neonatal disorders in low- and middle-income countries (LMICs) from 1990 to 2050, by sex. The vertical dashed line indicates the transition from the observed values (1990−2023) to the projected values (2024−2050)

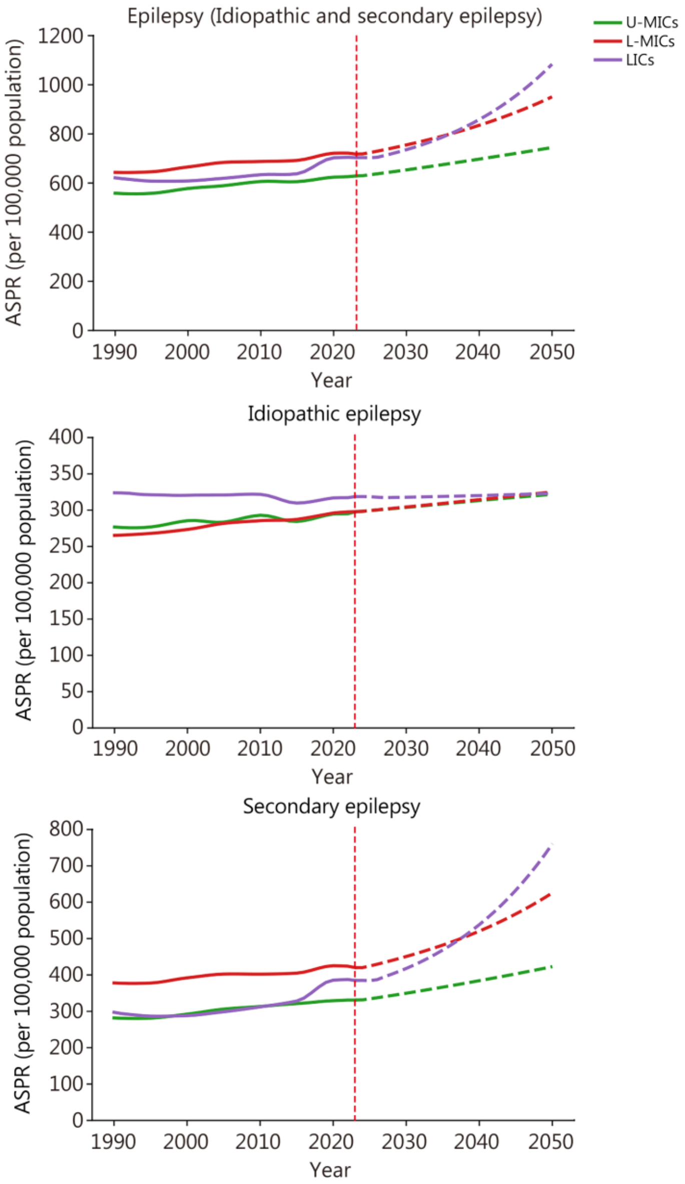

Fig. S3 Projected trends in ASPR of epilepsy (idiopathic and secondary), idiopathic epilepsy, and secondary epilepsy in low- and middle-income countries (LMICs), by income level, both sexes, 1990−2050. The vertical dashed line indicates the transition from the observed values (1990−2023) to the projected values (2024−2050)

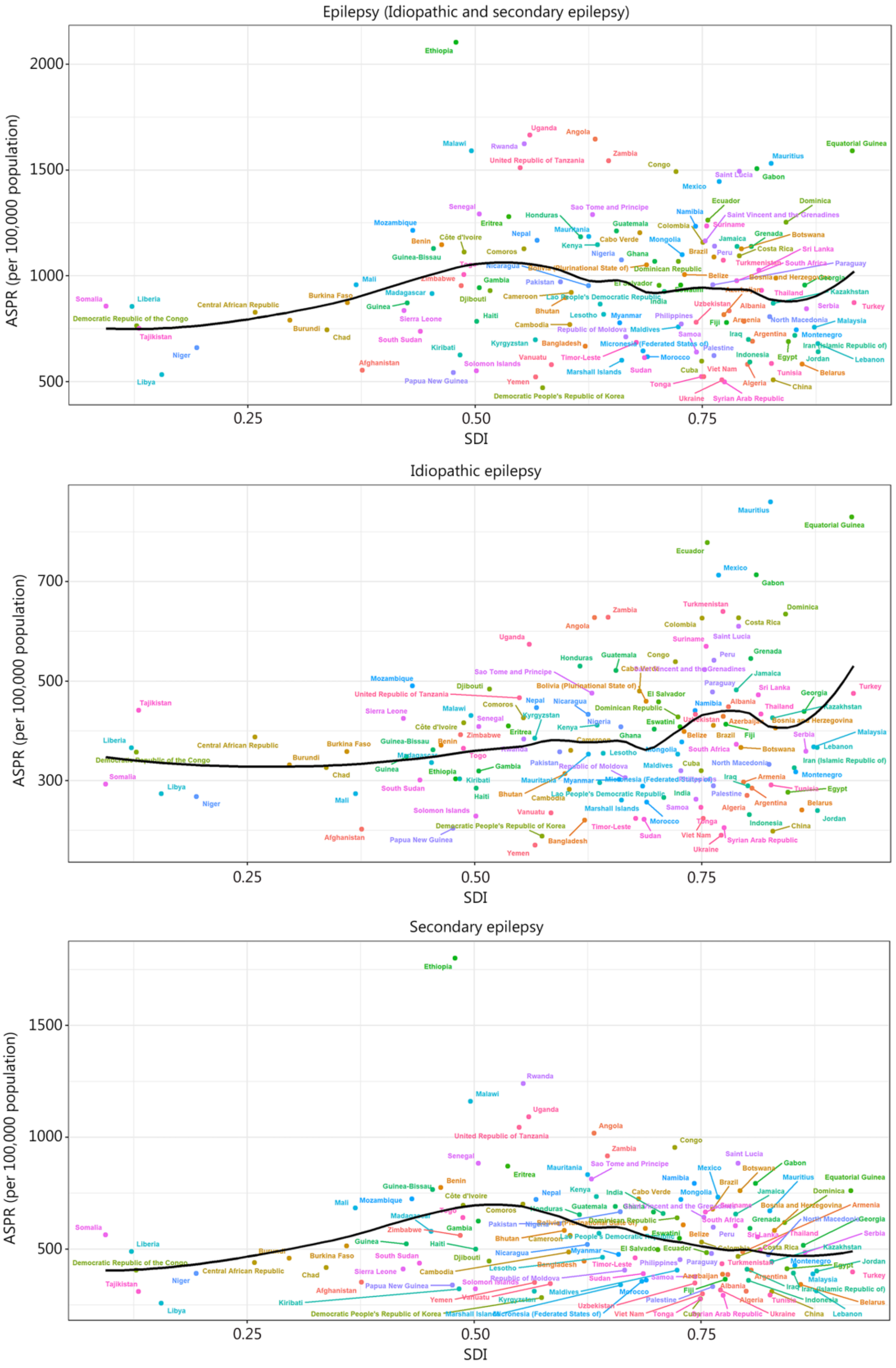

Fig. S4 Projected ASPR for epilepsy (idiopathic and secondary), idiopathic epilepsy, and secondary epilepsy in 129 low- and middle-income countries (LMICs) by SDI, both sexes, 2050. The black line represents the expected values based on the relationship between SDI and ASPR across all locations, estimated using locally estimated scatterplot smoothing (LOESS). SDI. Socio-demographic Index

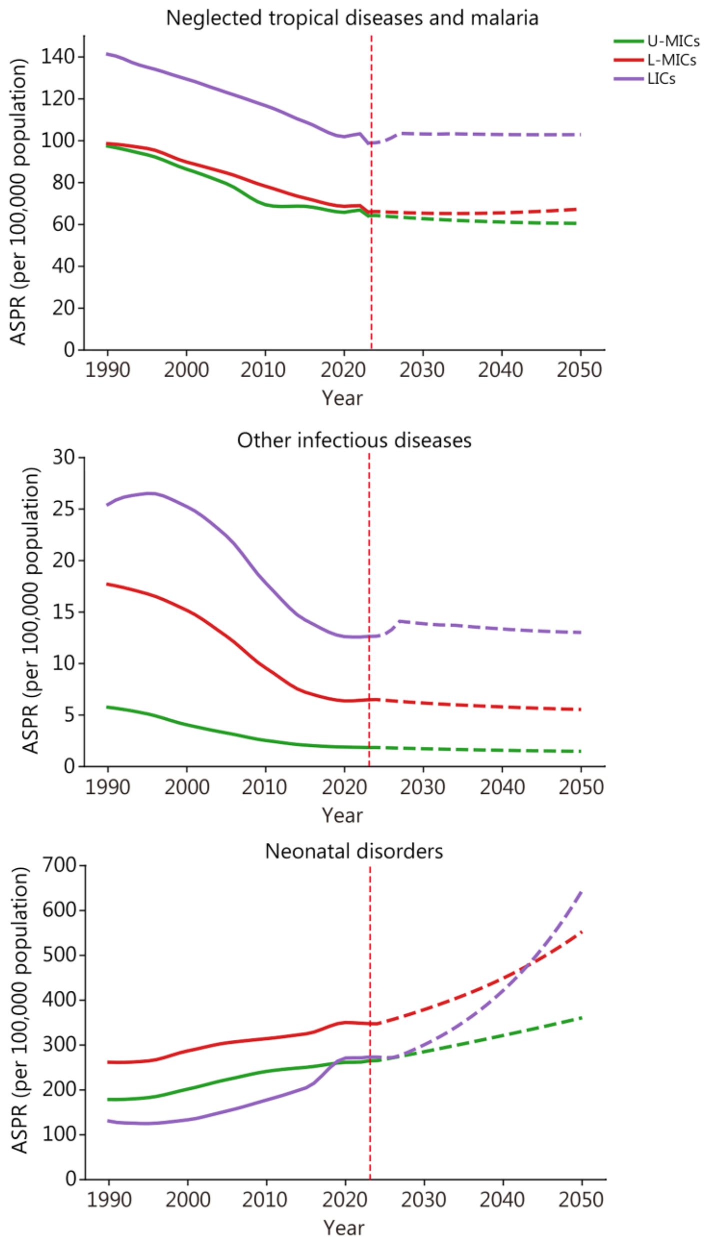

Fig. S5 Projected trends in ASPR of secondary epilepsy caused by neglected tropical diseases and malaria, other infectious diseases, and neonatal disorders in low- and middle-income countries (LMICs), by income level, both sexes, 1990−2050. The vertical dashed line indicates the transition from the observed values (1990−2023) to the projected values (2024−2050)

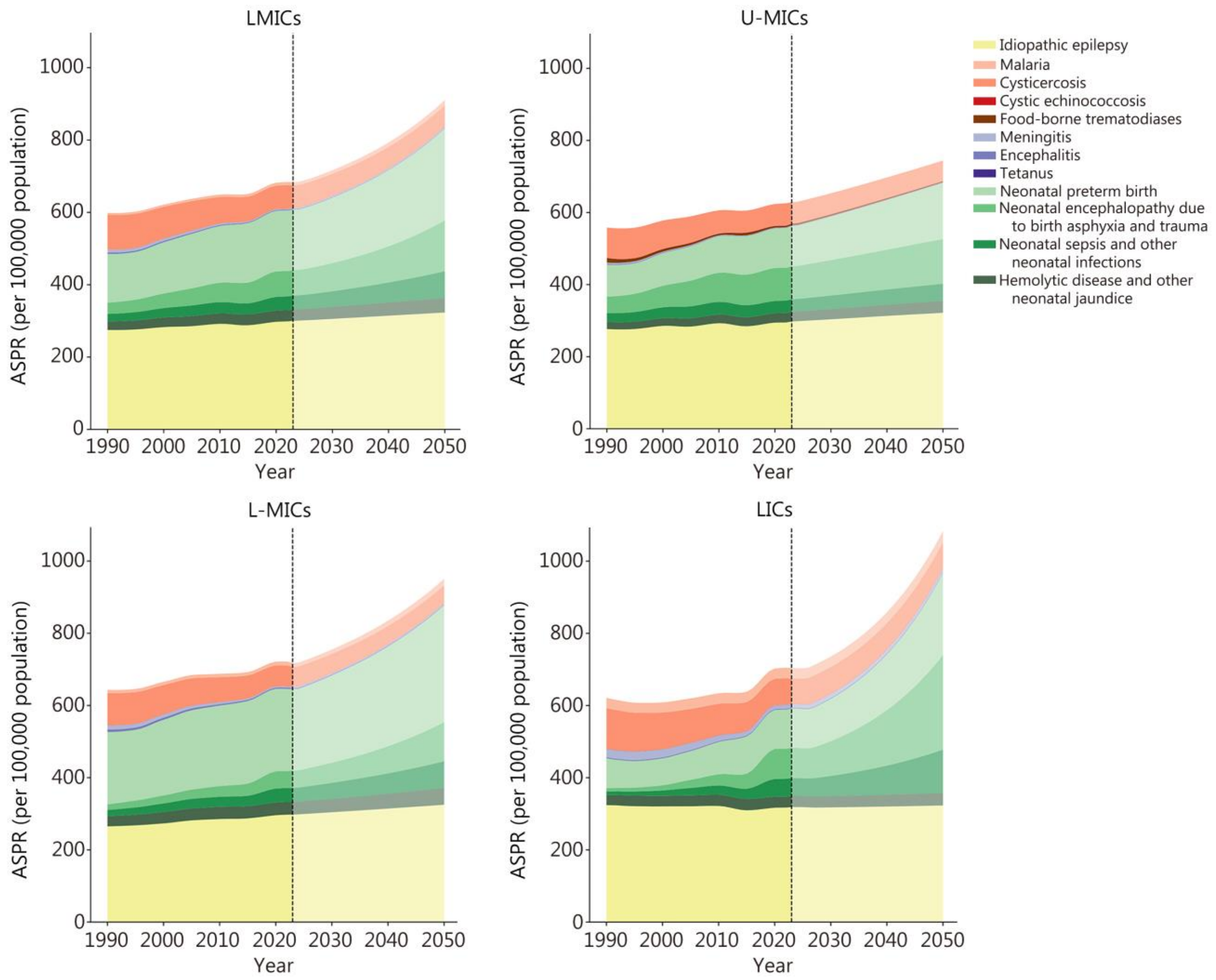

Fig. S6 Projected ASPR of idiopathic epilepsy and different causes of secondary epilepsy in low- and middle-income countries (LMICs), both sexes, 1990−2050. The vertical dashed line indicates the transition from the observed values (1990−2023) to the projected values (2024−2050). U-MICs. Upper-middle-income countries; L-MICs. Low-middle-income countries; LICs. Low-income countries

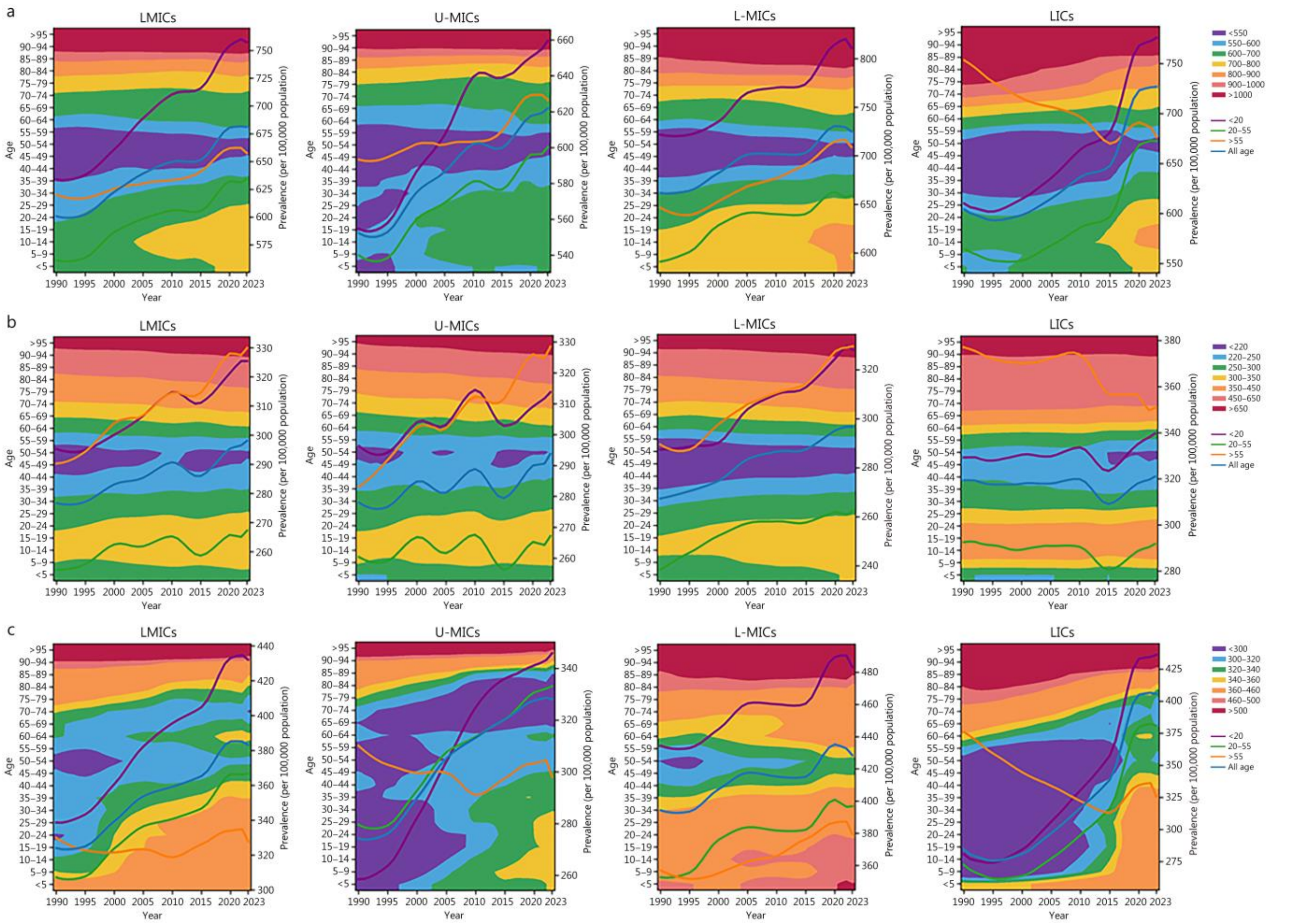

Fig. S7 Age-specific prevalence from 1990 to 2023 of epilepsy (idiopathic and secondary) (**a**), idiopathic epilepsy (**b**), and secondary epilepsy (**c**) in low- and middle-income countries (LMICs), both sexes. Age-specific analyses from 1990 to 2023 revealed distinct temporal trends across income groups. In LICs, the prevalence of epilepsy (idiopathic and secondary) increased markedly over time, particularly among younger age groups, whereas more gradual increases were observed in L-MICs and U-MICs. U-MICs. Upper-middle-income countries; L-MICs. Low-middle-income countries; LICs. Low-income countries

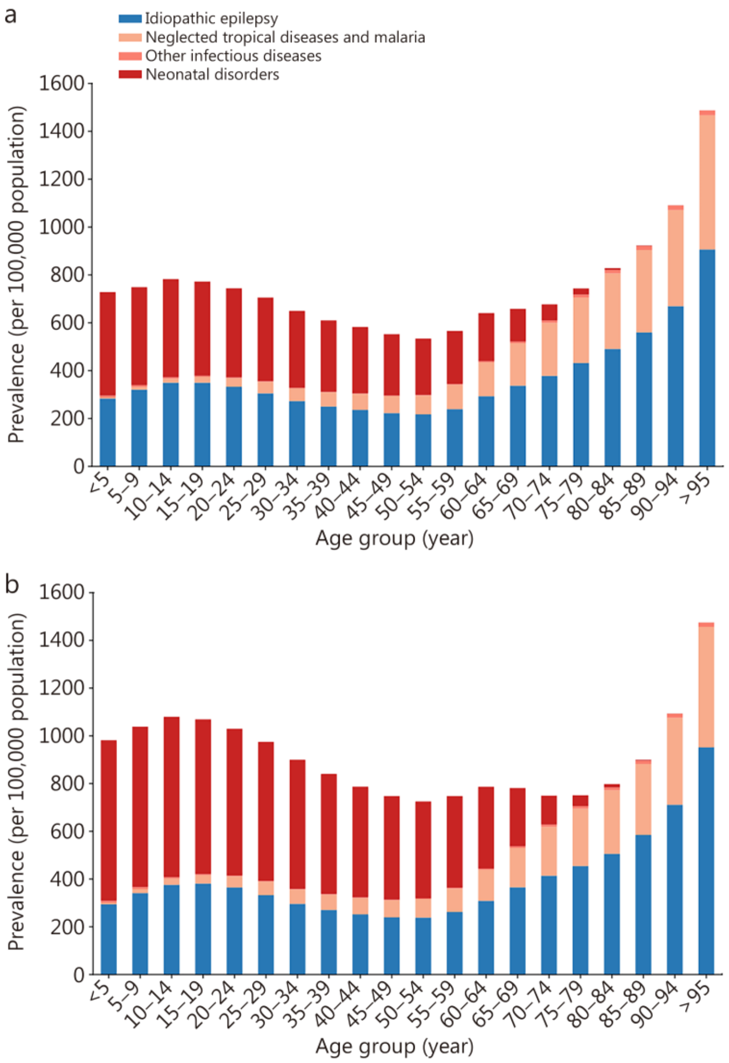

Fig. S8 Projected age-specific prevalence of idiopathic epilepsy and secondary epilepsy in low- and middle-income countries (LMICs), both sexes, 2023 (**a**) and 2050 (**b**)

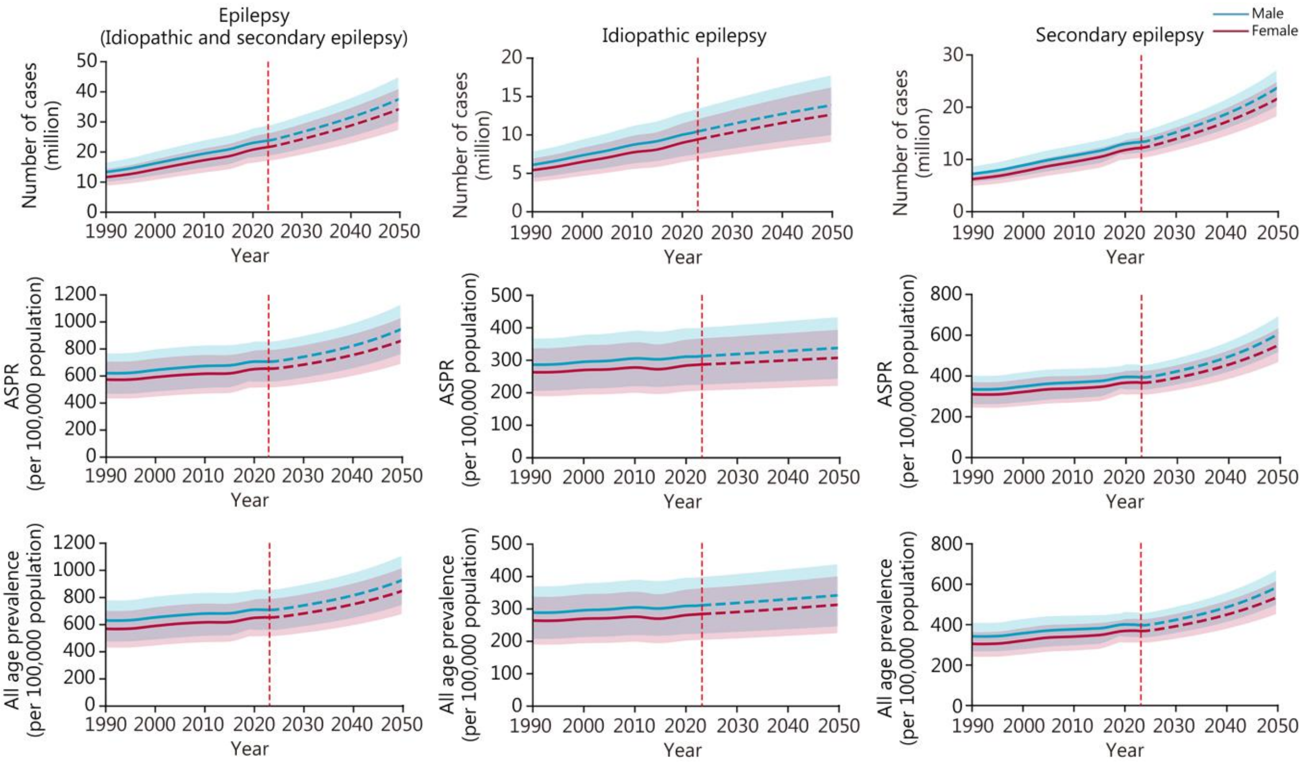

Fig. S9 Projected trends in number of cases, ASPR, and all age prevalence of epilepsy (idiopathic and secondary), idiopathic epilepsy, and secondary epilepsy in low- and middle-income countries (LMICs) from 1990 to 2050, by sex. The vertical dashed line indicates the transition from the observed values (1990−2023) to the projected values (2024−2050).

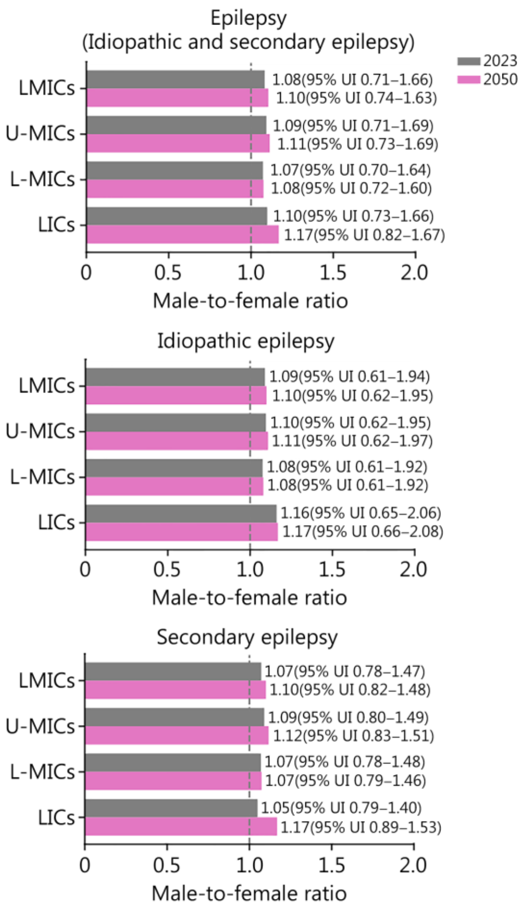

Fig. S10 Projected male-to-female ratio of ASPR of epilepsy (idiopathic and secondary), idiopathic epilepsy, and secondary epilepsy in low- and middle-income countries (LMICs), 2023 and 2050. Values are presented as male-to-female ratios with approximate 95% uncertainty intervals (UIs). Ratios were calculated from the male and female ASPRs, and the approximate 95% UIs were estimated by propagating the reported sex-specific uncertainty intervals. U-MICs. Upper-middle-income countries; L-MICs. Low-middle-income countries; LICs. Low-income countries

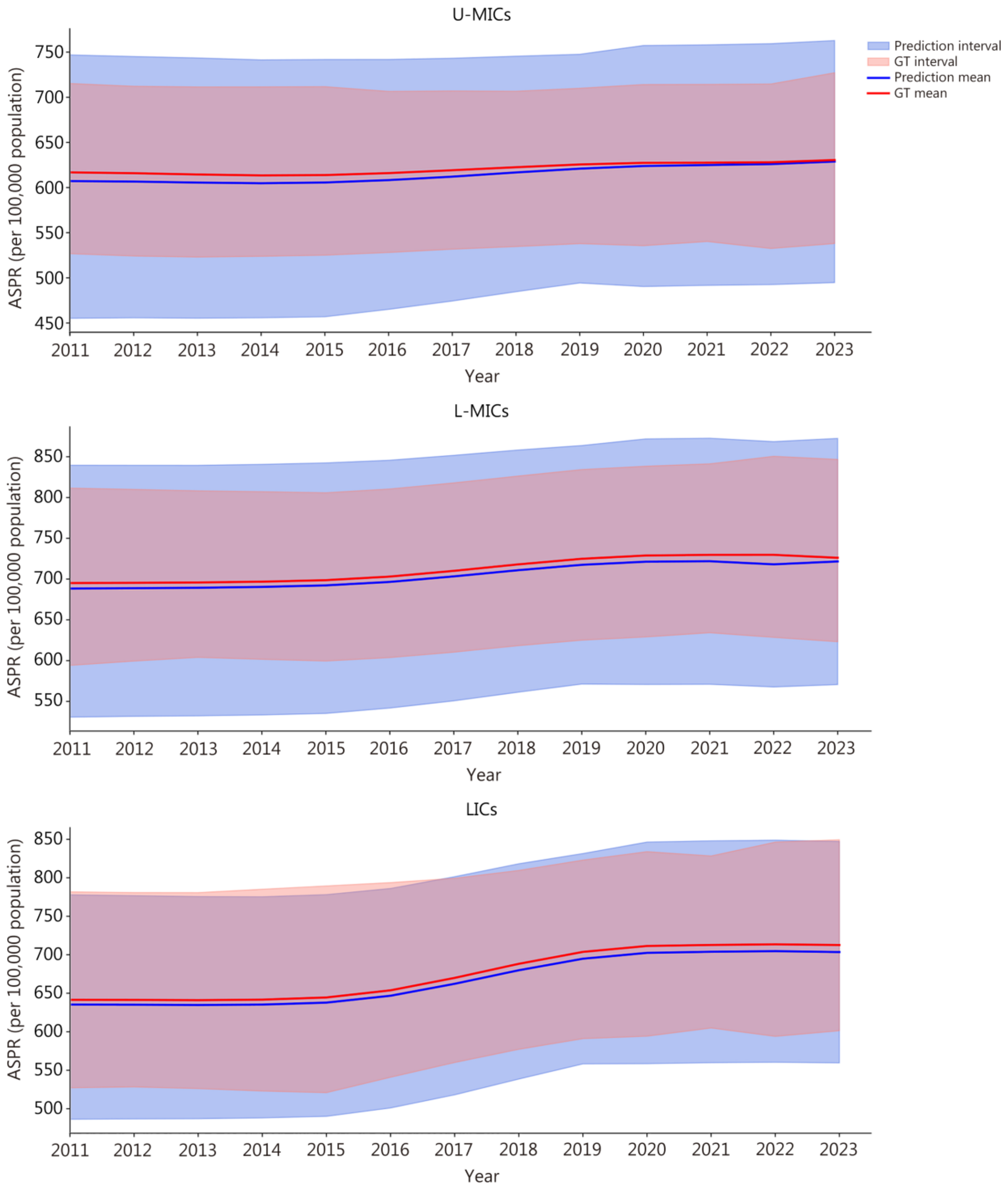

Fig. S11 Predicted and observed 95% UI for ASPR of epilepsy (idiopathic and secondary) in low- and middle-income countries (LMICs), both sexes, 2011−2023. Shaded areas denote uncertainty intervals (prediction in blue; observed in red). Consistent overlap between intervals (coverage ratio >90%) suggests good model calibration. U-MICs. Upper-middle-income countries; L-MICs. Low-middle-income countries; LICs. Low-income countries

References

1. Nichols E, Steinmetz JD, Vollset SE, Fukutaki K, Chalek J, Abd-Allah F, *et al*. Estimation of the global prevalence of dementia in 2019 and forecasted prevalence in 2050: an analysis for the Global Burden of Disease Study 2019. Lancet Public Health. 2022;7(2):e105-25.
2. World Bank. World Bank country classifications by income level for 2024−2025. https://blogs.worldbank.org/en/opendata/world-bank-country-classifications-by-income-level-for-2024−2025. Accessed 5 April, 2025.
3. Zelano J, Brigo F, Garcia-Patek S. Increased risk of epilepsy in patients registered in the Swedish Dementia Registry. Eur J Neurol. 2019;27(1):129-35. doi: 10.1111/ene.14043.
4. Arnaldi D, Donniaquio A, Mattioli P, Massa F, Grazzini M, Meli R, et al. Epilepsy in Neurodegenerative Dementias: a clinical, epidemiological, and EEG Study. J Alzheimers Dis. 2020;74(3):865-74. doi: 10.3233/JAD-191315.
5. Rochoy M, Bordet R, Gautier S, Chazard E. Factors associated with the onset of Alzheimer's disease: Data mining in the French nationwide discharge summary database between 2008 and 2014. PLoS One. 2019;14(7):e0220174. doi: 10.1371/journal.pone.0220174.
6. El Tallawy HN, Farghly WMA, Shehata GA, Rageh TA, Hakeem NA, Abo-Elfetoh N, et al. Prevalence of dementia in Al Kharga District, New Valley Governorate, Egypt. Neuroepidemiology. 2012;38(3):130-7. doi: 10.1159/000335655.
7. Smith K, Flicker L, Dwyer A, Atkinson D, Almeida OP, Lautenschlager NT, et al. Factors associated with dementia in Aboriginal Australians. Aust N Z J Psychiatry. 2010;44(10):888-93. doi: 10.3109/00048674.2010.491816.
